# Double crossed? Structural and computational studies of an unusually crosslinked haem in *Methylococcus capsulatus* cytochrome P460†

**DOI:** 10.1039/d5sc04213e

**Published:** 2025-08-08

**Authors:** Hans E. Pfalzgraf, Aditya G. Rao, Kakali Sen, Hannah R. Adams, Marcus Edwards, You Lu, Chin Yong, Sofia Jaho, Takehiko Tosha, Hiroshi Sugimoto, Sam Horrell, James Beilsten-Edmands, Robin L. Owen, Colin R. Andrew, Jonathan A. R. Worrall, Ivo Tews, Adrian J. Mulholland, Michael A. Hough, Thomas W. Keal

**Affiliations:** a Diamond Light Source Ltd, Harwell Science and Innovation Campus Didcot OX11 0DE UK michael.hough@diamond.ac.uk; b Research Complex at Harwell, Rutherford Appleton Laboratory Didcot OX11 0FA UK; c Centre for Computational Chemistry, School of Chemistry, University of Bristol Bristol BS8 1TS UK adrian.mulholland@bristol.ac.uk; d STFC Scientific Computing, Daresbury Laboratory Keckwick Lane, Daresbury Warrington WA4 4AD UK thomas.keal@stfc.ac.uk; e School of Life Sciences, University of Essex Wivenhoe Park Colchester Essex CO4 3SQ UK; f Graduate School of Science, University of Hyogo Hyogo Japan; g RIKEN SPring-8 Center 1-1-1 Kouto Sayo Hyogo 679-5148 Japan; h Department of Chemistry and Biochemistry, Eastern Oregon University La Grande Oregon USA; i Biological Sciences, Institute for Life Sciences, University of Southampton Southampton SO17 1BJ UK

## Abstract

Cytochromes P460 oxidise hydroxylamine within the nitrogen cycle and contain as their active site an unusual catalytic *c*-type haem where the porphyrin is crosslinked to the protein *via* a lysine residue in addition to the canonical cross links from cysteine residues. Understanding how enzymes containing P460 haem oxidise hydroxylamine into either nitrous oxide or nitric oxide has implications for climate change. Interestingly the P460-containing hydroxylamine oxidoreductase utilises a tyrosine crosslink to haem and performs similar chemistry. Previous crystal structures of cytochrome P460 from *Nitrosomonas europaea* (NeP460) clearly show the existence of a single crosslink between the NZ atom of lysine and the haem porphyrin, with mutagenesis studies indicating roles for the crosslink in positioning a proton transfer residue and/or influencing the distortion of the haem. Here we describe the evidence for a novel double crosslink between lysine and haem in the cytochrome P460 from *Methylococcus capsulatus* (Bath). In order to understand the complexities of this enzyme system we applied high resolution structural biology approaches at synchrotron and XFEL sources paired with crystal spectroscopies. Linked to this, we carried out QM/MM simulations that enabled the prediction of electronic absorption spectra providing a crucial validation to linking simulations and experimental structures. Our work demonstrates the feasibility of a double crosslink in McP460 and provides an opportunity to investigate how simulations can interact with experimental structures.

## Abstract 2

Combined high resolution structural biology approaches and multiscale computational modelling provides evidence for an unusual double crosslink between lysine and haem in a cytochrome P460 enzyme.

## Graphical abstract

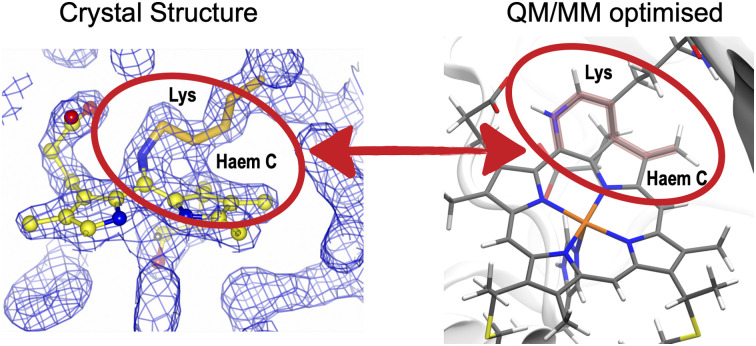

## Introduction

**Scheme 1:**
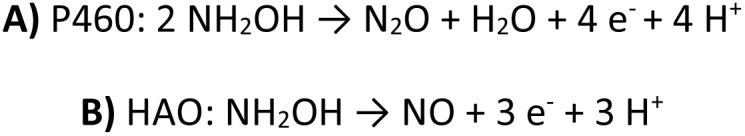
Reaction scheme for oxidation of hydroxylamine by (A) cytochrome P460 and (B) hydroxylamine oxidoreductase.^10^

The oxidation of hydroxylamine is a key part of the process of nitrification, whereby fixed nitrogen is converted to biologically available forms such as nitrate, in a range of bacteria. In this process, the reactive intermediate hydroxylamine is formed through the oxidation of ammonia by ammonia monooxygenases before being converted to a series of nitrogen oxides, including the damaging greenhouse gas nitrous oxide. Two enzymes carry out the challenging hydroxylamine oxidation reaction; both have unusual crosslinked haem active sites, which share a characteristic absorption maximum of 460 nm in the ferrous state.^1^ Hydroxylamine oxidoreductases (HAO) use a P460 haem with a crosslink between a tyrosine residue and the haem.^2,3^ In contrast, cytochromes P460 have a crosslink between a lysine and the haem.^4^ The P460 haem is thus characterised by a protein to porphyrin crosslink, in addition to the canonical crosslinks of two cysteine residues that occur in all *c*-type haems. HAO and cytochrome P460 differ in their reaction mechanism and product, despite acting on the same substrate (Scheme 1). Cytochromes P460 consist of a homodimer with one haem P460 per monomer. In contrast, HAO is a homotrimeric protein with each monomer containing seven electron transfer haems and a single P460 haem. For this reason, cytochromes P460 are more tractable for experimental and computational study. The properties and structures of several catalytically active cytochrome P460s have been characterised, particularly those from the ammonia oxidising *Nitrosomonas europaea* (NeP460)^5^ and methane oxidising *Methylococcus capsulatus* (Bath) bacteria (McP460).^6^ The structures reveal rather different active site pockets, but both are solvent filled, and highly polar with residues positioned suitably for electron and proton transfer. McP460 lacks the C-terminal helix seen in NeP460 but harbours three arginine residues in the active site. Crystal structures have been determined for NeP460 (ref. 5) and its crosslink-deficient mutant,^7^ for McP460,^6^ and for an inactive cytochrome P460 from *Nitrosomonas* sp. AL212 (ref. 8) (NsALP460) and its mutants including an active variant.^9^

All published crystal structures of these enzymes have been determined at 100 K using synchrotron radiation. While these structures are informative, electronic state changes around the haem group as a result of radiation damage are highly likely.^10–12^ Spectroscopic data from crystals have revealed that reduction typically takes place at doses far lower than those required for structure determination using conventional methods.^11^ Moreover, cryo-cooling of protein crystals can suppress functionally relevant dynamics and/or lead to the observation of structural artefacts.

Extensive work by Lancaster and co-workers on cytochromes P460 identified the role of the active site structure and the crosslink in positioning the haem and crucial residues involved in proton transfer, in influencing the haem geometry, and in the unusual spectroscopic properties of cytochromes P460. Site-directed mutagenesis of NsALP460 to introduce a glutamate residue (Glu131), mimicking the active site pocket of NeP460 (Glu97), led to gain of hydroxylamine oxidation activity.^9^ This activity can be removed by knocking out the crosslinking lysine,^13^ leading to a mutant enzyme with standard spectroscopic properties for *c*-type haem enzymes. This identified the glutamate in that position as important for proton transfer in the catalytic mechanism and the proposal that one role of the protein crosslink to haem was to correctly position this glutamate for proton transfer.

Molecular simulation studies of cytochrome P460s have been limited, mostly involving cluster calculations using density functional theory (DFT) on the catalytic P460 unit of HAO and omitting the role of the protein environment.^14,15^ A recent study by Siegbahn used DFT cluster calculations to study product formation in hydroxylamine oxidation by cytochrome P460 and HAO and to propose reasons for product formation favouring either NO or N_2_O.^16^ Furthermore, absorption spectra of the crosslinked haem moiety and inner-sphere ligands in NeP460 have been simulated with time-dependent density functional theory (TD-DFT).^8,16^ The spectroscopic calculations revealed that the experimentally observed spectral red shift in NeP460 can be attributed to geometric distortions of haem, in particular, the deviation of the macrocycle from planarity.^8^

In this work, we describe a structure of native McP460 obtained at room temperature using serial femtosecond crystallography (SFX) at an X-ray free electron laser (XFEL) to exclude effects of radiation damage to the sample that could affect the structure or electronic state. It is critical to have a true ground state structure that has not been modified by the data collection technique. This could not be achieved using synchrotron radiation and required the unique properties of an XFEL.

**Scheme 2:**
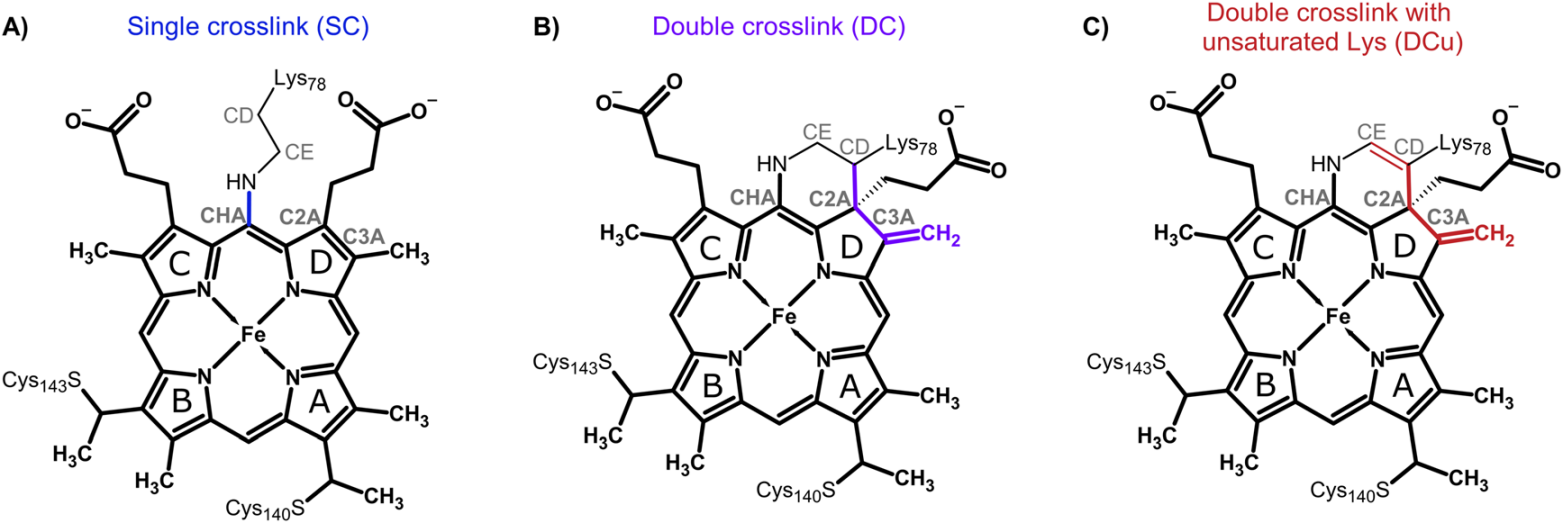
Schematic representation of the haem unit showing the different types of crosslinks considered for McP460: (A) single crosslink between Lys78 and the haem (SC); (B) double crosslink between Lys78 and the haem (DC); and (C) double crosslink with unsaturated lysine (DCu). Atom names are indicated in grey. Pyrrole ring names are labelled with black letters. Haem atoms are shown in bold. The bonds that undergo modification due to the crosslinks are coloured blue: SC, violet: DC, and red: DCu.

**Scheme 3:**
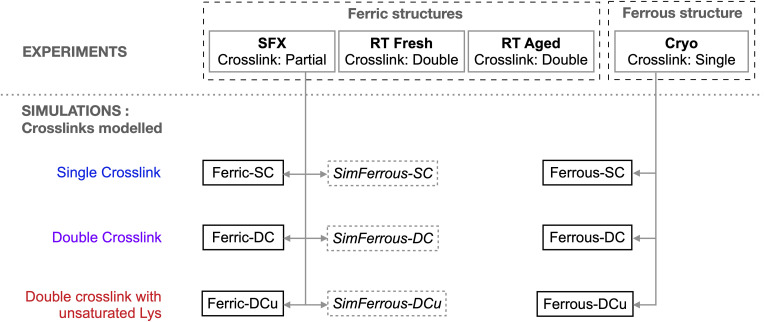
A workflow of the structures determined experimentally and those that were used as the basis for simulations. The ferric SFX structure was simulated in its Fe(iii) state for the single crosslink, double crosslink and double crosslink with unsaturated lysine side chain models, labelled Ferric-SC/DC/DCu respectively. The ferrous cryo structure was also used as the basis for simulation of all crosslink alternatives in the Fe(ii) state, labelled Ferrous-SC/DC/DCu. Finally, for validation purposes ferrous models were also generated from the ferric SFX structure, labelled SimFerrous-SC/DC/DCu.

We also describe structures validated by absorption spectroscopy carried out on crystals to explicitly identify the oxidation state and/or ligand state of the crystals. This enables a structure of the Fe(ii) (ferrous) enzyme and the iron-bound water ligand of the Fe(iii) (ferric) structure to be assigned. Crucially, we use these data to carry out hybrid quantum mechanical/molecular mechanical (QM/MM) molecular simulations of cytochrome P460, calculate spectra, and compare to experimental results. Our data suggest the presence of an unusual double crosslink between the haem and active site lysine residue, in contrast to the single crosslink to lysine that is present in NeP460. Possible chemical structures of the crosslinks observed in the experimental data are shown in Scheme 2: (A) a single covalent link between Lys78-NZ and haem-CHA (which we refer to as the single crosslink or SC model), (B) an additional covalent link between Lys78-CD and haem-C2A (double crosslink, DC) and (C) a double crosslinked species with a double bond in the Lysine sidechain between Lys78-CD and Lys78-CE (double crosslink with unsaturated Lys sidechain, DCu). These three alternatives were modelled into the active site of the crystal structures and structural and spectral determination were carried out *via* QM/MM simulations to identify the nature of the crosslink species present in the oxidised and reduced X-ray structures. Scheme 3 shows which experimental structures were used as the basis for simulations of the different oxidation states of Fe and crosslink types. This study provides an exemplar of close interplay between molecular simulation and experiment, used here to understand the structure of the covalently linked Lys-haem unit in this challenging enzyme.

## Methods

### Activity assays and N_2_O and NO_2_^−^ quantification

Recombinant McP460 was expressed and purified as described previously.^6^ To validate the recombinant protein, activity assays were carried out as previously described.^3^ Production of N_2_O was determined by gas chromatography-mass spectrometry using a Shimidzu GC-2014 equipped with an Porapak-Q-column (80/100 mesh 1 m × 2.3 mm I.D). All reactions were prepared in 10 mL septum-sealed headspace vials. For anaerobic reactions, samples were prepared in a glovebox. Turnover conditions were achieved with 5 μM cyt P460, 6 μM PMS, 2 mM hexaammineruthenium(iii) chloride ([Ru(NH_3_)_6_]Cl_3_), and 0 μM, 100 μM, 250 μM, 500 μM, or 1000 μM NH_2_OH in 500 μL of buffer total. Reactions were initiated by the addition of NH_2_OH, after which the vial was quickly capped and inverted three times. The headspace was sampled after reactions were allowed to proceed overnight. N_2_O production was quantified by integrating the peak corresponding to N_2_O (retention time = 2.13 min). Nitrite production was quantified using a Griess diazotization assay. Samples were prepared aerobically with 5 μM cyt P460, 6 μM PMS, 2 mM ruthenium hexachloride, and 0, 100 μM, 250 μM, 500 μM, or 1000 μM NH_2_OH in 500 μL of buffer total.

### Crystallographic data collection and processing

#### Serial femtosecond crystallography

Microcrystals were grown in batch using a modification of the crystallisation conditions previously used for growing large single crystals, namely 3.8–4.0 M ammonium sulphate, 0.1 M Tris pH 8 in a 1 : 1 ratio of protein : crystallisation condition and 1 μL of microseeds. The final protein concentration in the batches ranged from 20 to 30 mg mL^−1^. Crystals grew in 1–2 days at 18 °C to dimensions of 44 × 41 × 18 (±9 × 11 × 6, *n* = 20) (Fig. S1†). To remove the precipitate, batches were centrifuged at 2300*g* for 1 min, leading to sedimentation of the crystals as a dark green band surrounded by precipitate. The precipitate was carefully removed with a pipette and the crystals were resuspended in 3.8 M ammonium sulphate, 0.1 M Tris pH 8. This process was repeated 1–2 times until most of the precipitate had been removed, as judged by inspection under a standard microscope. To generate the microseeds, crystals grown by vapour diffusion as previously described^6^ were disrupted using seed beads (Hampton Research). Crystals were transported at ambient temperature. A slurry of batch micro crystals (300 μL total) was loaded onto two silicon fixed target chips with 9–10 μm apertures^17^ for SFX data collection at the SACLA XFEL, BL2 (Harima Campus, Japan) using a Rayonix MX300-HS detector. The experimental set up has been previously described in detail.^18^ The X-ray pulse length was 10 fs and the repetition rate was 30 Hz. The pulse energy was 360 microjoules and the photon energy 11 keV (FWHM 25.6 eV). The beam size was 1.65 μm horizontally and 1.29 μm vertically. Data were collected at 28 °C. SFX data were processed using xia2.ssx^19^ with a total of 29 559 crystals included in the final dataset. A resolution cut-off was imposed at the point where the correlation coefficient fell monotonically below 0.3. CCP4i2 (ref. 20) was used to generate a free R set and REFMAC5 (ref. 21) and Coot 0.9 (ref. 22) were used to iteratively refine and build the structures based on the previously published 100 K structure.^6^ The program qFit 3.2.2 (ref. 23) was used to suggest alternate conformations based on a composite omit map generated with Phenix.^24^ The resulting structures are denoted SFX in Scheme 3.

#### Room-temperature synchrotron crystallography

*In situ* room-temperature crystallography was used to obtain the structure of McP460 with a single conformation of the crosslinking Lys78 (double-crosslinked). A Mosquito® LCP liquid-handling robot (SPT Labtech) was used to set *In Situ*-1™ crystallisation plates (MiTeGen). It dispensed 100 nL of McP460 at 7 mg mL^−1^ (based on a molecular weight of 15.6 kDa and an extinction coefficient of 78.5 mM^−1^ cm^−1^ at 419 nm) in 20 mM Tris–HCl pH 8.0, to which was added a total of 100 nL of precipitant solution (2.05 M ammonium sulphate, 100 mM Tris–HCl pH 8.0) including crystal seed suspension made by manually crushing the batch crystals leftover from SFX experiments with a Crystal Crusher (Hampton Research) 200 times. The resulting drops were equilibrated against 35 μL of precipitant solution in the reservoir. Crystals appeared within 1 day. X-ray diffraction data at 20 °C were collected directly from crystals within the crystallisation plate on beamline VMXi (Diamond Light Source).^25–27^ The flux of the attenuated 10 × 10 μm beam was 9.93 × 10^11^ ph s^−1^ at 16 keV. Wedges of 60° rotation were recorded using a Eiger2 X 4M detector (Dectris). The “fresh” X-ray diffraction dataset was collected from 45 wedges 2 d after starting the crystallisation (Fig. S2A†). The “aged” dataset was collected from 35 wedges on other crystals (Fig. S2B†) 51 d after starting the crystallisation, by which time the crystals had stopped growing. The data were processed with multi-xia2 dials^28^ in ISPyB by specifying the cell parameters and space group. CCP4i2 (ref. 20) was used to generate a free R set and REFMAC5 (ref. 21) and Coot 0.9 (ref. 22) were used to iteratively refine and build the structures based on the SFX structure. JLigand^29^ was used through CCP4cloud^30^ to prepare the restraints for haem DCu. The resulting structures are denoted RT fresh and RT aged in Scheme 3.

#### Anaerobic cryocrystallography

Obtaining a reduced (ferrous) crystal structure required extensive optimisation of an anaerobic procedure together with cryo-trapping of the reduced form to avoid reoxidation. The crystal was obtained in an anaerobic chamber (Belle Technology) by sitting-drop vapour diffusion in XRL™ plates with Micro-Bridges® (Hampton Research) sealed by ClearVue™ sheets (Molecular Dimensions). The reservoir contained 1 mL of precipitant solution (1.9 M ammonium sulphate, 100 mM Tris–HCl pH 8.0). The drop contained 1 μL of this precipitant solution, 1 μL of seed stock in the same precipitant solution and 2 μL of McP460 at 7 mg mL^−1^. The large fragments in the seed stock had been removed by centrifugation at 100*g* for 2 min. Crystals appeared within 3 d and were harvested at 4 d inside the anaerobic chamber. To do so, 3.4 μL of cryoprotectant were added to the drop (precipitant solution containing 1.7 M sodium malonate) and after 7 min, 0.6 μL of precipitant solution containing 1 M sodium dithionite (SDT) was added (for a final SDT concentration of 0.1 M in the drop). This caused a visible change in crystal colour from brownish green to green (Fig. S3†). After 10 min, the crystal was harvested in a mounted 80 μm clear CryoLoop (Hampton) and cryocooled from within the anaerobic chamber. X-ray diffraction data were collected at 100 K on beamline I24 (Diamond Light Source) using a CdTe Eiger2 9M detector (Dectris). A 50 × 50 μm beam of X-rays at 20 keV delivered 0.20 MGy of radiation dose for the first dataset and a further 0.30 MGy for the second dataset.^31^ The data were processed with xia2 dials. CCP4i2 (ref. 20) was used to generate a free R set and use REFMAC5 (ref. 21) and Coot 0.9 (ref. 22) were used to iteratively refine and build the structures based on the previously published 100 K structure.^6^ The resolution cut-off of 1.33 Å was chosen using PAIREF^32^ as including data from 1.35 Å to 1.33 Å decreased the *R*_free_ value. The resulting structures are denoted ferrous cryo in Scheme 3.

#### Single-crystal spectroscopy analysis

UV/visible absorption spectroscopy from protein crystals maintained at 100 K in a nitrogen cryostream was performed at Diamond beamline I24 using an off-axis microspectrophotometer. A fibre coupled xenon light source (Thorlabs), a Shamrock 303i spectrometer (Andor Technology) and a Newton EM CCD detector were used. Data were collected over the wavelength range 203–751 nm with a focal spot of 50 × 50 μm for the white light incident on the crystal. The ferrous crystal was maintained at 100 K in a nitrogen cryostream. Each spectrum was an accumulation of 10 exposures of 9.95 ms. Spectroscopy data were measured prior to and following X-ray data collection, with the crystal was returned to its original orientation following diffraction data collection in order to ensure that orientation-dependent changes to the visible spectrum were avoided. The ferric crystal was harvested using a nylon loop mounted onto a reusable B3S-R base, which was inserted into a MicroRT™ tube (MiTeGen). This plastic sleeve was filled with enough mother liquor to have the crystal near but not immersed in it. Each spectrum was an accumulation of 50 exposures. No diffraction data were collected. Smoothing was performed in Origin by LOESS of span 7%.

#### Solution UV-visible spectroscopy

McP460 at 7 mg mL^−1^ in 20 mM Tris pH 8.0 was added as a 10 μL aliquot to 1 mL of each pH 8.0 solution. Absorption spectra were recorded from 190 nm to 840 nm (1 nm resolution) in a reduced volume polystyrene cuvette of path length 1 cm on a Nanodrop 2000c (Thermo Fisher). Smoothing was performed in Origin by LOESS of span 7%.

### Molecular simulation of cytochrome P460

#### Determination of P460 haem partial charges

P460 has a unique haem C unit with an additional covalent bond between the NZ atom of Lys78 and CHA of haem. Partial charges were assigned following the established conventions for CHARMM forcefields.^33^ A cluster model (Fig. S4†) consisting of the haem cofactor (without the propionate and methyl sidechains) with the sidechains of Lys78, covalently linked Cys140, Cys143 and coordinated His144 was built from the published 6HIU^6^ structure for charge evaluation. DFT geometry optimisations were carried out using the NWChem^34^ program with the B3LYP^35,36^-D3 (ref. 37) functional and 6-31G*^38,39^ basis set. The CB–CA bonds for the amino acid residues were terminated by hydrogen atoms and the CB positions were kept fixed to their crystallographic coordinates during the DFT optimisations. Optimisations were performed in both oxidised (ferric) and reduced (ferrous) forms of Fe treated in three different spin states: doublet (*M* = 2), quartet (*M* = 4) and sextet (*M* = 6) for the ferric form and singlet (*M* = 1), triplet (*M* = 3) and quintet (*M* = 5) for the ferrous form, respectively. The optimisations were followed by an electrostatic potential (ESP) charge calculation with constraints to ensure that chemical groups had an overall neutral charge and similar groups had equivalent charges, in line with CHARMM conventions (a detailed description is provided in the ESI, Section S.1†). The charges used correspond to the lowest energy spin state for the optimised cluster and are provided in the ESI (Table S1†).

#### Modelling the crosslinks

**Fig. 1:**
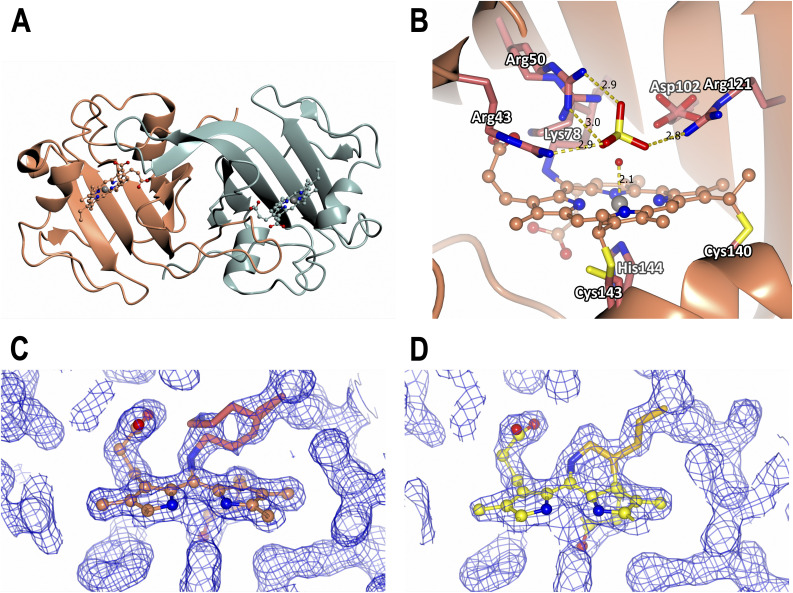
Room temperature crystal structures of McP460. The polypeptide is represented in ribbons, the haem cofactors in ball and stick and the amino acid sidechains as sticks coloured by atom type. (A) Ferric SFX whole protein with chain A in sea green and chain B in brown, (B) ferric SFX active site with bound sulphate from the crystallization condition. Bond lengths are indicated in Å. (C) Ferric SFX crosslink electron density, (D) RT fresh crosslink electron density. Dashed lines indicate hydrogen bonds. The blue meshes represent 2Fo–Fc electron density maps contoured at 0.44 and 0.30 electrons per Å^3^, respectively.

The damage-free, high resolution ferric SFX structure was used as a starting structure for generating the alternative crosslink species. The nature of the crosslink in the ferric SFX structure is ambiguous, with main conformation Lys78-CD–haem C2A distances of 1.94 Å and 2.11 Å for chain A and B, respectively. Single crosslink (SC) and double crosslink (DC) geometries were created from this structure (Fig. 1) and then partial optimisation of the haem unit around the crosslink was performed to relax them. DFT cluster calculations were performed with all haem atoms fixed except those involved in the crosslink and their two immediate neighbouring atoms (see Fig. S5†). Both oxidised and reduced forms of Fe were calculated for each of the spin states used in the charge determination step. The resulting optimised geometries from all spin states were similar for both the oxidised ferric and reduced ferrous state. The structural parameters around the SC and DC haem units were consistent across all spin states and oxidation states of Fe and are provided in the ESI (Section S.2†). The resulting optimised DC and SC haem C centres in the lowest spin state (*M* = 2 for ferric and *M* = 1 for ferrous) were aligned to the crystal haem C unit of the ferric SFX structure. The alignment showed good agreement at the CA–CB bond junctions in Lys78, Cys140, Cys143 and His144 (Fig. S6†). The haem C unit from the ferric SFX structure was then replaced by the optimised SC and DC haem units, thus providing the starting structures for the SC and DC models.

#### Simulation setup

The generated Ferric-SC and Ferric-DC structures were prepared for simulations by removing all double occupancy mainchain and sidechain atoms and partial water molecules, keeping those with greater fractional occupancy or lower *B*-factors. Protonation states of the titratable residues of McP460 crystal structure were set at pH 8 to be consistent with the pH of crystallisation using the propKa^40,41^ module of the PDB2PQR^42^ suite of programs, followed by visual inspection of sidechain environments; all ionisable residues were in their standard protonation states, with histidine residues singly protonated at ND. PDB2PQR was also used to add missing hydrogen atoms to the protein structures. Water molecules in the crystallographic structure were retained. The partial charges for the Ferric-DC model were adjusted in line with the CHARMM forcefield conventions for charge determination and are provided in the ESI (Table S1†). The same protocol was repeated to set up the ferrous models, starting from the ferrous cryo structure.

The above generated structures were subjected to minimisation to optimise hydrogen atom positions, while maintaining heavy atoms at their crystallographic coordinates. Once minimised, the structures were used for QM/MM simulations. As in previous work,^43^ our QM/MM model is a finite cluster representation and does not consider long range periodicity, but we do not expect this to have a significant influence on the local chemical environment around the haem–lysine crosslink.

#### QM/MM calculations

##### QM/MM partitioning for single crosslink models

The haem C unit was included in the QM region along with the covalently linked Lys78, Cys140 and Cys143, the coordinated His144 and water 275, which is coordinated to the Fe atom. The methyl and propionate sidechains of the haem unit were not included in the QM region^44^ (Fig. S7A†). All the residues were truncated at the CA–CB bond and capped with hydrogen atoms. Atoms within 4 Å of the QM region were relaxed (active region) during QM/MM geometry optimisations, while the remaining atoms were frozen. The size of the unconstrained region was restricted to a small 4 Å window to allow the geometry around the QM region to relax, thus avoiding extensive relaxation of the outer residues of the protein during optimisation as the haem C residue is surface exposed.

##### QM/MM partitioning for double crosslink models

To model the DC crosslink the same residues were considered as in the SC structure, with the addition of the terminal –CH_2_ of pyrrole D ring of the haem C which is involved in the formation of this double crosslink (Fig. S7B†).

QM/MM calculations were performed with the Python-based version of the ChemShell computational chemistry environment^45^ using NWChem and DL_POLY^46^ for DFT and molecular mechanics calculations, respectively. The biomolecular QM/MM workflow in ChemShell^47^ was used to perform the calculations, including DL_FIELD^48^ to import forcefield parameters in CHARMM (version 36)^49,50^ format. The DL-FIND^51^ library was used for geometry optimisations. Electrostatic embedding with charge shift correction was used to represent the surrounding MM environment. The level of theory used for the QM/MM calculations is B3LYP-D3 and the def2-SVP basis set was used for all QM atoms except Fe, which was treated with the def2-TZVP basis set.^52,53^ Optimisations were carried out in three spin states: *M* = 2, 4 and 6, for the ferric state of Fe. For the Ferrous-SC systems, the system was optimised in spin states *M* = 1, 3 and 5. Additionally, as this is a dimeric protein, calculations were performed on both chains, considering one haem in the QM region at a time.

#### Molecular dynamics setup

After preparation of the SC and DC structures for Ferric-SC/DC and Ferrous-SC, they were solvated with a 20 Å layer of TIP3P^54^ water in all three directions using a pre-equilibrated cubic water box available *via* the solvate plugin module in VMD.^55^ The ferric state was electroneutral, so no additional ions were added, while for the ferrous state two sodium counterions were added to achieve electroneutrality during simulations using the autoionisation feature in VMD. All-atom molecular dynamics (MD) simulations were performed on these systems using NAMD^56^ with the CHARMM36 forcefield.^49,50^ These simulations employed Langevin dynamics with periodic boundary conditions at 300 K. Long-range electrostatics were treated by the particle mesh Ewald method. The cutoff for nonbonded parameters was set at 12 Å with a smooth switching function turned on from 10 Å. In NPT simulations, the pressure was maintained with the Langevin piston method. All the systems were initially subjected to 5000 steps of conjugate gradient minimisation to eliminate any unphysical contacts. Then, the water and ions were equilibrated in an *NVT* ensemble, keeping the protein fixed for 2 ns. This was followed by 100 000 steps of minimisation of the protein H-atoms to keep the protein structure close to the crystal geometry. Next, the solvent was allowed to further relax by equilibrating the systems for 175 ns in an NPT ensemble, keeping all atoms of the protein fixed. In addition, for Ferric-SC/DC systems, after minimisation, the systems were allowed to relax for 175 ns in an NPT ensemble keeping the backbone harmonically restrained with a force constant of 25 kcal mol^−1^ Å^−2^ while allowing the sidechains and solvent to relax.

#### Spectral calculations

The QM/MM optimised crystal structures from chain A were used to compute the absorption spectra of the single and double crosslink models. Spectral calculations in ChemShell were carried out using ORCA version 5.0.3 (ref. 57) which provides a wide range of quantum chemical methods for calculating excitation energies. Excitation energies were computed for different spin states of the ferric and ferrous forms using simplified time-dependent density functional theory (sTD-DFT) and time-dependent density functional theory (TD-DFT). The semi-empirical method ZINDO/S^58^ is an alternative cheap and effective method (parameterised for lower spin states) known to perform well for transition metal-containing organic biomolecules. We also used ZINDO/S to compute excitation energies for the lower spin states. The sTD-DFT^59^ and TD-DFT^60^ calculations were carried out with the range-separated hybrid functional CAM-B3LYP^61^ with a def2-SVP basis set on all atoms other than Fe, which was treated using def2-TZVP (as for ground state optimisations). Oscillator strengths were extracted in the dipole length representation. The resulting spectra were produced by convolution of the first 30 transitions for the ferrous form and 50 transitions for the ferric form using a Gaussian function with a broadening of 0.15 eV. The electronic transitions were analysed in terms of natural transition orbitals (NTO) generated using ORCA.

## Results

### Serial femtosecond crystallography structure of cytochrome P460

McP460 was recombinantly expressed and purified. To confirm its activity, assays were carried out to assess the protein's ability to oxidise hydroxylamine, and product formation. The previously reported activity of NeP460 at 4.5 μMDCPIP per μMCytP460 per min is around 3.8 times higher than that of McP460 (ESI Fig. S8†). The activity of McP460, 1.19 μMDCPIP per μMCytP460 per min, is more similar to that of the NsALP460 A131E mutant at 2.1 μMDCPIP per μMCytP460 per min.^62^ This suggests that while McP460 can oxidise hydroxylamine, it is not as efficient as NeP460. Production of nitrous oxide/nitrite by the wt protein was confirmed though GC-MS and Griess assays. Under anaerobic conditions only nitrous oxide was detected, whilst under aerobic conditions both nitrous oxide and nitrite were produced (ESI Fig. S8†).

Microcrystals of McP460 were generated and used for serial crystallography. We first sought to obtain structures of ferric McP460 free of any artefacts rising from radiation damage, cryoprotectants or temperature-dependent effects. Such a structure provides a good starting point for molecular simulations. The most effective means to achieve this was to use serial femtosecond crystallography (SFX) at an XFEL where the very short (10 fs) pulse duration allows diffraction data to be measured from room temperature microcrystals without sufficient time being available for radiation chemistry to occur (the principle of diffraction before destruction). Using room temperature microcrystals also avoids artefacts from cryoprotection or flash cooling. Accordingly, SFX data were measured at the SACLA XFEL and produced a structure to 1.28 Å resolution (Table 1).

**Table 1:** Experimental data statistics for the crystal structures of McP460 reported here

Dataset	Ferric SFX	RT fresh	RT aged	Ferrous cryo
Resolution (Å)	1.28–29.34 (1.28–1.326)	1.66–58.4 (1.66–1.719)	1.77–58.31 (1.77–1.833)	1.33–58.35 (1.33–1.378)
Space group	*P*2_1_2_1_2_1_	*P*2_1_2_1_2_1_	*P*2_1_2_1_2_1_	*P*2_1_2_1_2_1_
Unit cell (Å)	46.778, 81.002, 85.139	46.657, 80.627, 84.695	46.701, 80.468, 84.612	46.380, 80.441, 84.780
Merged crystals	29 559	45	35	1
Temperature (K)	301.3	293.5	293.5	100
Unique reflections	84 024 (8280)	38 507 (3807)	31 820 (3124)	73 436 (7171)
Completeness (%)	99.96 (99.98)	99.72 (97.35)	99.31 (93.63)	98.52 (85.86)
Multiplicity	173.5 (180.6)	80.5 (33.7)	66.5 (37.5)	6.4 (4.4)
CC_1/2_	0.999 (0.317)	0.999 (0.428)	0.982 (0.285)	0.998 (0.254)
*R*_pim_				0.05157 (1.06)
*I*/*σI*	28.24 (0.81)	18.92 (0.51)	15.88 (0.54)	8.89 (0.44)
*R*_work_	0.1304 (0.3605)	0.1425 (0.3760)	0.1451 (0.3727)	0.1558 (0.3694)
*R*_free_	0.1600 (0.3817)	0.1826 (0.3857)	0.1964 (0.3464)	0.1998 (0.3831)
RMSD bond length (Å)	0.013	0.015	0.014	0.013
RMSD bond angles (°)	2.07	2.17	2.21	1.92
Ramachandran favoured (%)	98.94	98.92	99.28	98.59
Dose (MGy)	—	0.57	0.52	0.34
PDB code	9HS9	9HS4	9HS6	9HRK

As for the previous structure from a single crystal at 100 K, cytochrome P460 forms a homodimer with each monomer adopting a largely beta sheet fold connected by several loops. Superposition of the SFX structure with the 100 K structure (PDB 6HIU) yielded an RMSD value of 0.30 Å over 286 residues. The region of the backbone that differs most between cryogenic conditions and room temperature is the loop from Thr85 to Ser91, which harbours the Phe89 residue that is in contact with the haem from the other monomer. Indeed, this loop is shifted by approximately 0.5 Å in chain A and 0.4 Å in chain B (Fig. S9A and B†) based on analysis by Gesamt.^63^ At room temperature, it is therefore closer to the haem of the other chain. Unlike the previously published 100 K structure where a cryoprotectant molecule is bound to Arg43 (Fig. S9C†), the SFX structure shows a sulphate molecule from the crystallisation condition on that site (Fig. 1B). This may explain the small differences in the conformations of the nearby Arg50, which could affect its interactions with the propionate of the haem (Fig. S9C†). In the 100 K structure (PDB 6HIU) a single conformer of the Arg50 side chain is present positioned to interact with the heme propionate. In the SFX structure two different conformers of Arg50 are present, both rotated relative to 6HIU and only one being in position to interact with the propionate. The haem groups and haem pockets in the SFX structure of ferric P460 reveal six coordinate Fe(iii) geometry with a His at the proximal face, with an Fe–His (N) bond length of 2.1 Å (Table S2†). An indicator of radiation damage in the 100 K structure is the iron–water distance of over 2.3 Å compared to the 2.1 Å distance in the damage-free SFX structure.^64^ This shorter iron–water distance in the SFX structure could explain the why the sidechain of its Asp102 is 0.2 Å closer to the iron centre (based on the distance to the CG carbon) than in the 100 K structure.

**Fig. 2:**
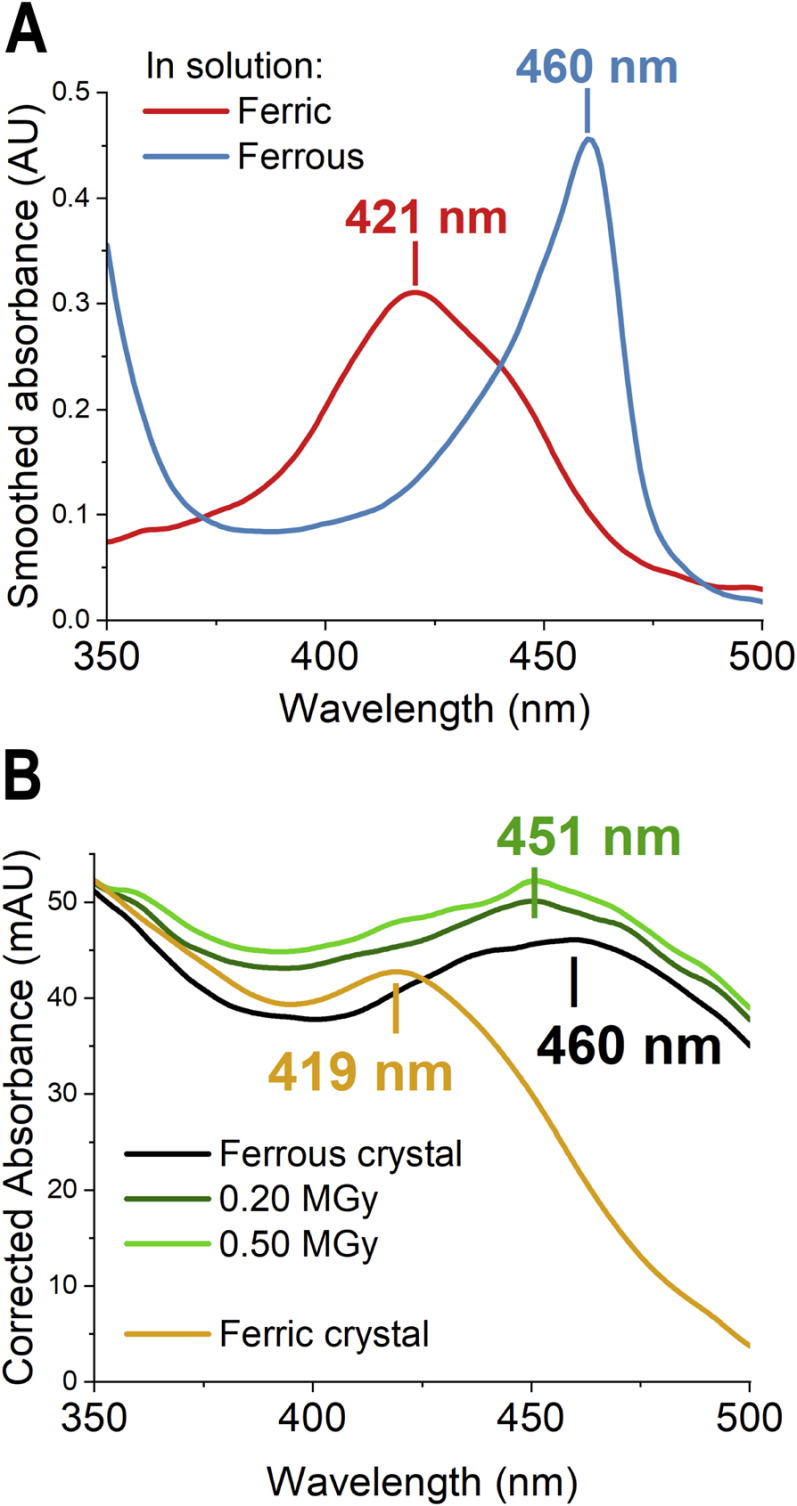
Experimental UV-vis spectra of McP460 at pH 8.0 (A) in solution (smoothing of 2.5% only due to the sharp 460 nm peak) and (B) in crystals. A clear shift is evident upon change of oxidation state.

Intriguingly, and in contrast to the previous 100 K structure of NeP460, the SFX structure of McP460 to very high resolution contained electron density features suggestive of a second crosslink between Lys78 and the porphyrin. Indeed, the model of the main conformation of Lys78 causes an overlap between its CD carbon and the C2A carbon of the haem. At 1.4 Å in chain A and 1.3 Å in chain B, this overlap is 0.2 Å more severe than in the published 100 K structure, which already suggested a potential second crosslink, although it was not noted at the time. The chemical nature of this new modification remained unclear due to the multiple conformations of the Lys78 sidechain. Intriguingly, a spectral shift occurs in the UV-vis spectrum of McP460 on exposure to ammonium sulphate in solution (Fig. S10†). Single-crystal spectra consistently show spectra consistent with ferric protein and we do not observe any evidence of ammonia binding in crystals (Fig. 2B).

### Fresh and aged crystal structures (double-crosslink only)

To ascertain whether degradation of crystals over time was responsible for the variability in observed crosslinking, we collected diffraction data from crystals *in situ* 2 d after setting the crystallisation plate, and again 7 weeks later using other crystals from the same plate. Pleasingly, the fresh crystals yielded a structure with a clearly resolved conformation of Lys78. The aged crystals gave a structure that was essentially identical, albeit at a lower resolution. This suggests that the additional Lys78 conformation is not caused by degradation over time. We could only speculate that it is due to a combination of the batch of protein and the method of crystallisation.

**Fig. 3:**
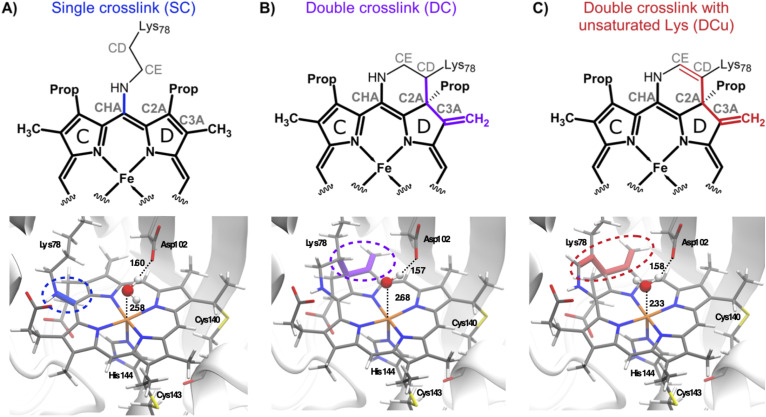
QM/MM optimised geometries of (A) single crosslink, SC, (B) double crosslink, DC and (C) double crosslink with unsaturated Lys side chain, DCu. The haem C unit is shown with sticks and the different crosslinks are shown in the same colour as in Scheme 1. The 6th site coordinated water is shown in ball and stick. The active site residue Asp102 which interacts with the coordinated water is shown in sticks. Distances are given in Å.

This structure collected on fresh crystals clearly shows a second crosslink between Lys78 and the porphyrin: a covalent bond between the CD carbon of Lys78 and the C2A carbon of the porphyrin (Fig. 1D). Since there are now four carbons bonded to C2A, they must all do so through single bonds to satisfy its valence. This means the C2A–C3A bond has been changed from double to single (Fig. 3). The geometry around C3A remained clearly planar in the structure, so we assign it an exocyclic double bond, as evidenced by a shorter bond length to the terminal carbon on this tetrapyrrole ring D compared to all others (1.46 Å *vs.* 1.52 Å when using restraints without this double-bond; 1.33 Å when using double-bond restraints).

When examining the geometry around the CD of Lys78 in the electron density, it was unclear whether it had four covalently bonded atoms (with a hydrogen pointing towards the iron-bound water) or three bonded atoms thanks to an additional double bond on the lysine. We therefore reduced model bias by calculating a composite omit map (Fig. S11†). This shows a mostly planar arrangement of atoms around the Lys78 CD, supporting a double bond on the lysine sidechain. This also provides the reactivity needed to form a second crosslink, especially if near to a heteroatom. We therefore tentatively built a double bond between the CD and CE carbons of Lys78 in the RT fresh and RT aged models.

Electron density near the haem-linked sulphur atom of Cys143 was assigned to a minor alternate conformation of the residue. Since this other sulphur position is close to bonding distance from the haem carbon CAC (1.8 Å), it was also modelled as linked (Fig. S12†). Because of the nearly planar geometry around CAC from this new bond, there could remain a C–C double bond to the terminal carbon CBC. This can unfortunately not be distinguished within the mainly single bond from the major conformation of Cys143. We do not expect the UV-vis spectrum to be affected as this double bond is not in plane of the haem and therefore would not be conjugated with its π-system.

The iron in the RT fresh structure is likely to have remained in the ferric state, as X-rays seem unable to reduce it in McP460 based on single-crystal UV-vis spectroscopy (Fig. S13†). The RT fresh structure is very similar to the damage-free SFX structure (RMSD 0.14 Å across 286 residues). Fewer alternate conformations in the RT fresh structure were modelled, due to its lower resolution. Most differences are in the alternate conformations (such as Gln46, Fig. S9C†), which were not used in simulations of the SFX structure. The iron–water distances are similar in both structures (2.2 Å), Table S2.† The RT aged structure is essentially identical, apart from fewer waters and alternate conformations due to a lower resolution. The conformations of Gln46 in the active site are slightly different, but it is a very mobile residue, evidenced by its high temperature factors. Both structures contain two conformers of Arg50 in a similar manner to the SFX structure.

### Cytochrome P460 in the ferrous state (structure reduced *in crystallo*)

**Fig. 4:**
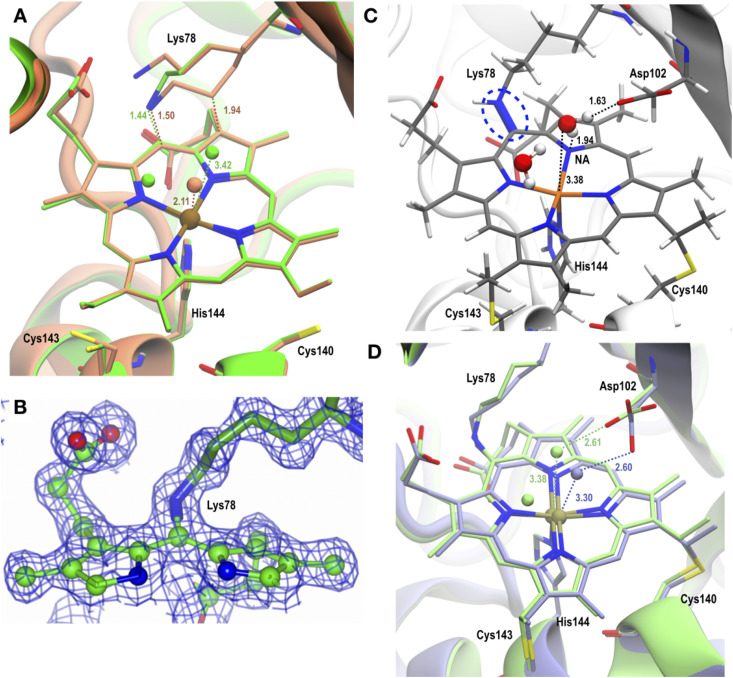
(A) Ferrous cryo structure (green) overlapped with ferric SFX (brown) structure; the crosslink with Lys78 and water(s) near Fe are shown. The active site residues and H-atoms are omitted to give a clear view around the haem unit and crosslink(s). (B) Electron density map as blue mesh (2Fo–Fc contoured at 0.44 electrons per Å^3^ in chain B) showing SC character of the ferrous cryo structure. (C) QM/MM optimised Ferrous-SC showing SC (highlighted as blue dashed lines), active site residue Asp102 and the water molecules near Fe. (D) QM/MM optimised Ferrous-SC (light green) overlapped with QM/MM optimised SimFerrous-SC (light blue); the water(s) near Fe and active site residue Asp are shown. The H-atoms are not shown to enable a clear view around the haem unit and SC. All distances are in Å.

We next sought to understand the nature of the haem structure in the ferrous form of the enzyme. Initial attempts to reduce the protein in the crystalline state using chemical reagents or by exposure to X-rays were unsuccessful as assessed by electron density (checking for movement of the iron coordinated water molecule) and crystal spectroscopy (checking for optical spectra changes that would indicate reduction). Preparation of crystals in an anaerobic glove box followed by incubation with sodium dithionite and cryotrapping resulted in successful generation of one crystal in the ferrous form as assessed by single-crystal spectroscopy of cryo-trapped crystals after reduction, Fig. 4 and S14.† The difficulty of obtaining a stable reduced form rendered it infeasible to obtain a room temperature or SFX structure of the ferrous protein. The optical absorption spectrum revealed a large spectral shift relative to the as-isolated ferric form consistent with successful reduction. Crystallographic data were measured from this reduced crystal, yielding a structure at 1.33 Å resolution. A superposition of the ferrous structure with the previous ferric structure (PDB 6HIU in Fig. S14†) and with the SFX structure of the intact ferric state (Fig. 4A) revealed structural changes including the iron atom being five-coordinate in the ferrous form (and therefore moved towards the distal side of the plane of the haem, see Tables 2 and S2†) and the loss of the iron-coordinated water molecule present in the ferric form. Instead, a water is 3.1 Å from the nitrogen of either pyrrole ring A or D, with an additional water 3.1 Å from the nitrogen of pyrrole ring C. Residue Lys78 only shows the single crosslink between its sidechain nitrogen and the γ*-meso* carbon of the haem (CHA carbon), not the crosslink between its CD carbon and the C2A carbon of the haem (Scheme 1B and C). Consequently, the terminal carbon of the pyrrole ring D is single-bonded (1.51 Å).

**Table 2:** Haem C site parameters for McP460 structures, including: distance between proximal His143 N and Fe (Fe–HisN), distance between the coordinated water to Fe (Fe–water), Fe to porphyrin N of pyrrole ring D (Fe–PyrNA), distance between Lys78 and CHA of haem (LysN–CHA), distance between CD of Lys78 and C2A of haem in DC systems (LysCD–C2A), distance between haem C3A and CMA (C3A–CMA) that undergoes exocyclic modification in DC systems, and Fe out of plane distance (Fe-OOP)

	Fe–HisN (Å)	Fe–water (Å)	Fe–PyrNA (Å)	LysN–CHA (Å)	LysCD–C2A (Å)	C3A–CMA (Å)	Fe-OOP (Å)
**Experiments**							
Ferric SFX	2.14	2.11	2.03	1.46	1.94	1.43	0.06
Ferric RT fresh	2.11	2.15	2.06	1.40	1.58	1.33	0.11
Ferrous cryo	2.10	3.33	2.10	1.44	—	1.53	0.35

**QM/MM simulations**							
Ferric-SC (*M* = 6)	2.09	2.58	2.08	1.35	—	1.47	0.24
Ferric-DC (*M* = 6)	2.11	2.68	2.22	1.34	1.57	1.35	0.20
Ferric-DCu (*M* = 2)	1.93	2.33	2.10	1.38	1.54	1.35	0.12
Ferrous-SC (*M* = 5)	2.11	3.38	2.14	1.35	—	1.47	0.40
Ferrous-DC (*M* = 5)	2.11	3.26	2.15	1.37	1.57	1.35	0.30
Ferrous-DCu (*M* = 5)	2.13	3.21	2.14	1.38	1.54	1.35	0.25
SimFerrous-SC (*M* = 5)	2.16	3.30	2.08	1.41	—	1.47	0.29
SimFerrous-DC (*M* = 5)	2.15	2.61	2.11	1.37	1.56	1.35	0.21
SimFerrous-DCu (*M* = 5)	2.16	2.5	2.12	1.39	1.53	1.35	0.19

The single-crystal UV-vis spectrum of the anaerobically reduced crystal showed a 460 nm absorbance peak (Fig. 2B), which matches the value from solution experiments. However, after collection of the X-ray diffraction data (0.20 MGy dose), the peak shifted to 451 nm. The structure presented here came from a second diffraction dataset from the same crystal (additional 0.30 MGy of dose), so that the UV-vis spectra before and after its collection both had a peak at 451 nm. The electron density for the 0.20 MGy structure (data not shown) was indistinguishable from that of the subsequent 0.50 MGy ferrous cryo structure presented herein.

### Computational studies on ferric models: energies and geometrical parameters

P460 is a homodimeric protein and the parameters around haem from the experimental ferric SFX structure indicate a very similar haem C geometry in both chains. As each simulation contains only one chain's haem unit in the QM region, the two chains, chain A and chain B, are independent systems in our QM/MM simulations. The lowest energy spin state was sextet (*M* = 6) for the chain A haem for both the single crosslink (Ferric-SC) and double crosslink (Ferric-DC) models. The energies are provided in the ESI (Table S3A†) and the optimised structures are shown in Fig. 3. The results for chain B are also provided in Table S3A.† The two chains, however, on many occasions optimised to different spin states: in chain B the lowest energy spin states for Ferric-SC and DC systems were *M* = 4 and *M* = 2, respectively. The relative energy of chain B spin states for Ferric-SC and DC with respect to the lowest *M* = 6 state of chain A are −2.5 and −1.1 kcal mol^−1^, respectively. These differences are comparable to or smaller than variations in QM/MM energies between the spin states of individual chains, hence for the subsequent spectral calculations, only chain A was considered.

We also considered the possibility of the DCu crosslink variant, with a double bond between CD and CE atoms of lysine, as tentatively assigned for double-crosslinked species from the obtained crystal structures (RT fresh). The charges were adjusted to accommodate this double bond (Section S.1 and Table S2†). The systems were then optimised at the QM/MM level from the corresponding DC predecessors. The DCu system optimised to the doublet state (*M* = 2) for chain A. Chain B, by contrast, optimised to the *M* = 4 state. The energy of this system was −2.1 kcal mol^−1^ lower than *M* = 2 of chain A. The energies are provided in the ESI (Table S3A†) and the structure in Fig. 3C.

Geometrical parameters around the haem for chain A are given in Table 2. The Fe out-of-plane (Fe-OOP) is measured as the distance between the centre of mass of the four N atoms of the porphyrin ring to that of Fe. The haem site QM/MM optimised geometries are very sensitive to the spin states (see ESI, Table S4A†). The average distance for Fe–His in the QM/MM optimised structures is 2.04 ± 0.10 Å, within the observed distance of 2.11 ± 0.01 Å. The Fe–water distance is longer than that observed experimentally; 2.53 ± 0.18 Å compared to 2.08 ± 0.03 Å from experimental structures. The measured Fe-OOP distance depends on the spin state of the optimised geometry. For the lowest energy spin state for the DCu crosslink type (*M* = 2), the Fe-OOP distance is in good agreement with the experimental value, while higher energy spin states showed less agreement. For the DC and DCu systems, the Fe–PyrNA distance is lengthened by ∼0.1 Å in *M* = 6 state compared to the *M* = 2 state. This Fe–PyrNA distance belongs to the pyrrole ring D harbouring C2A atom to which the second crosslink with CB-Lys78 is formed. Such elongation is also observed, though to a lesser extent, in the crystal structures (ESI Table S1†).

There are subtle differences in the geometric parameters around the haem C in the two chains. The values for chain B are provided in ESI Table S4A.† For all type of crosslinks considered, the Fe–HisN bond lengths are longer in chain B than A, and both chains show similar lengthening of the Fe–PyrNA distance, but the main difference is observed in Fe-OOP distances. In chain B, there is less variation in Fe-OOP distance with increase in spin multiplicity, and in all cases it is comparable to the Fe-OOP distance measured from the crystal structures (ESI Table S1†).

Beyond the coordination sphere of Fe in ferric SFX, there are alternative crystallographic conformations of active site residue Asp102, oriented perpendicular to each other; one conformation was modelled for chain A and the other for chain B (ESI Fig. S15†). Asp102 forms an H-bond with the water coordinated on the 6th site of Fe in chain A, while in chain B, Asp102 forms an H-bond with Gln100. Different conformations in chain A and B cause a different pattern of H-bonds around the active site. To test this, we removed the environmental effect by calculating the QM energies in gas phase for the DC systems. Without the environment, the energies for the two chains match and they both optimise to the same lowest energy spin state, *M* = 4 (ESI Table S5†). The other spins states, *M* = 2 and 6 are 2.8 and 5.7 kcal mol^−1^ less stable than the *M* = 4 state, respectively.

The difference in the conformation of Asp102 in the active site is also reflected in the water distribution around Fe during MD (see analysis in ESI, Section S.3 and Fig. S16†). The conformation of Asp102 in chain A facilitates water in the 6th coordination site of Fe. This results in shorter Fe–water distances of 2.7 ± 0.2 Å and 2.8 ± 0.2 Å for SC and DC systems, respectively, negligible interaction with pyrrole N, and strong H-bond interaction with Asp102. In chain B, the water does not occupy the 6th coordination site of Fe; the distance is 3.2 ± 0.2 Å and 3.3 ± 0.2 Å, for SC and DC, respectively. As in chain A, it has negligible interaction with pyrrole, however, it also has ∼50% less interaction with Asp 102. This difference in water occupation near Fe, is not seen in the second set of MD simulations where the sidechains were allowed to relax. The results reveal restoration of similar distribution of water around the active site in both chains (ESI Fig. S17†) for both Ferric-SC and DC systems. In the SC system, the Fe–water distances are 2.9 ± 0.2 Å and 2.8 ± 0.3 Å for chain A and B, respectively and for DC systems they are 2.6 ± 0.2 Å and 2.6 ± 0.2 Å for chain A and B, respectively. The water has negligible interaction with pyrrole N atoms for both chains. The interaction with Asp102 is similar in the two chains but quite different between SC and DC systems. In the SC system, water predominantly interacts with Asp102, whereas in the double-crosslink system, it interacts with both Asp102 and Arg50.

### Computational studies on experimental ferrous structures

The experimental ferrous cryo structure showed a CD Lys–C2A haem distance of ∼3.7 Å, indicative of a single crosslink structure. QM/MM optimisation of the Ferrous-SC model resulted in a high spin, quintet (*M* = 5) optimised state for both chains. The QM/MM optimised structure for chain A is shown in Fig. 4C and the haem C site parameters are provided in Table S4B.† Results from chain B are provided in the ESI (Table S4B†). In contrast to the single crosslinked model in the ferric state, the geometrical parameters of Ferrous-SC are in good agreement with the experimental structure: the active site water, (which occupied the 6th coordinate site of Fe in the Ferric-SC structure) is >3 Å away from Fe and the Fe-OOP distance is in accordance with the X-ray structure (Tables S2 and S4B†).

In this ferrous cryo structure, both chains have equivalent conformations of Asp102. MD simulations on Ferrous-SC were analysed for the distribution of water near Fe as for the Ferric-SC system. The last 150 ns of the 175 ns production trajectory reveals that the average distance of Fe–water is 3.3 ± 0.1 Å and 3.4 ± 0.1 Å for chain A and B, respectively. The water in chain A had a greater propensity to form H-bonds with the N atom of pyrrole ring D than in chain B. The same pattern among the chains is reflected in the interaction of the water with active site Asp102 residue (ESI Fig. S18† left panels A(I) and B(I)).

A long CD–C2A distance of ∼3.7 Å in the ferrous cryo structure suggests an SC structure. However, for comparison, DC and DCu models were also generated and simulated. A DC system structure was generated *in silico* by first aligning the gas phase optimised DC model (as used in Ferric-DC) onto the crystal structure, and then replacing the crystal haem unit with the DC haem unit, followed by QM/MM reoptimisation. The optimised geometry is given in Fig. S19† (I, middle panel) and energies are in Table S3B.† This Ferrous-DC structure optimised to a quintet state and the geometric parameters around haem are similar to its SC counterpart (Table 2 and ESI Table S4B†). The DCu system was also created from the DC structure and optimised (Fig. S19† (I, right panel) and Table S3B† for energies and Table S4B† for geometric parameters).

### Comparison with *in silico* reduced systems

The SC form of the ferric SFX structure was reduced *in silico* by adding an extra electron to the Ferric-SC system to create a new system denoted SimFerrous-SC. QM/MM calculations on the protein model and solvation followed by MD were carried out with the same protocol as for the Ferric-SC system. QM/MM optimisation gave a quintet (*M* = 5) lowest energy state for chain A. The optimised geometry of this SimFerrous-SC is overlaid on Ferrous-SC in Fig. 4D and the geometric parameters are provided in Table 2 for chain A. There is similarity between the two structures in Fe–water distance, Fe-OOP distance and overall structure of the haem. There is a longer Fe–water distance and prominent out of plane movement of the Fe, similar to the ferrous cryo structure. The energies and geometries for all spin states and for both chains are provided in ESI Tables S3C and S4C.†

Analysis of MD investigated the same properties, namely water molecules within 3.5 Å from Fe and interactions with haem and active site residues. The residence time of water near Fe shows a difference in distribution between chain A and B, as in the Ferric-SC system. It is also distinctly different from the Ferrous-SC structure. Chain A shows a bimodal distribution of water molecules in this SimFerrous-SC system, one at 2.6 ± 0.1 Å and the other at 2.9 ± 0.1 Å. In this SimFerrous-SC system, the water in chain A H-bonds with the N-atom of pyrrole ring D for more than 50% of time, which is not seen in chain B. Also, the interaction with Asp102 is more pronounced in chain A than B (See Fig. S18† let panels A(II) and B(II)). We also simulated the other crosslinks, DC (SimFerrous-DC) and DCu (SimFerrous-DCu), for this *in silico* reduced structure. The optimised geometries are given in Fig. S19(II),† and the haem parameters in Table 2 for chain A. The lowest energy spin state was also a quintet, as for Ferrous-DC and Ferrous-DCu structures. Haem parameters are given in Table S4C.†

### Computational spectroscopy of ferric and ferrous models

**Fig. 5:**
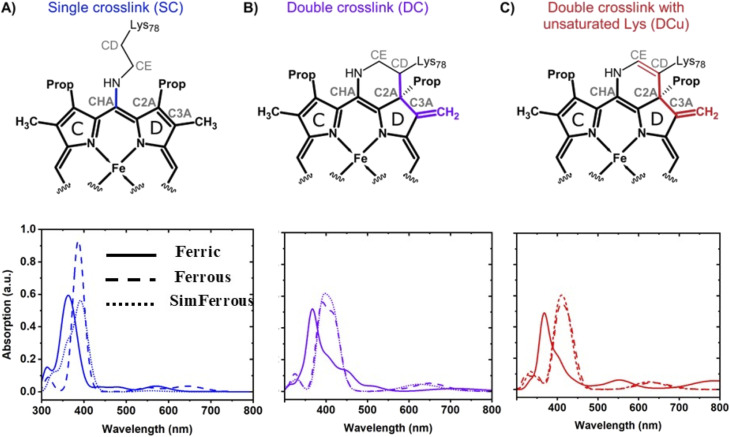
Computed absorption spectra for the three crosslink models using CAM-B3LYP (TD-DFT). Ferrous and SimFerrous forms for all models are in a quintet spin state whereas the ferric forms of DC and DCu are in a doublet spin state and SC is in sextet spin state. The computed absorption spectra for the individual models are given below the structures of each proposed model.

Computation of electronic absorption spectra is a useful way to help distinguish species present in a crystal or in solution. To shed light on the nature of the crosslink and the species present, we computed absorption spectra for the crosslinked models in both ferrous and ferric forms. As standard computational methods cannot reliably predict absolute values of peaks and/or lineshapes in the spectra of haem proteins, we focus only on the observed experimental spectral shift between the oxidised and reduced forms. The absorption spectra of the ferric SFX and ferrous cryo structures computed using TD-DFT QM/MM are given in Fig. 5. For comparison, the experimental absorption maxima of the Soret band for McP460 in the ferrous and ferric forms observed in solution are 460 nm (2.69 eV) and 419 nm (2.96 eV) respectively, Fig. 2.

The computed absorption maxima comprising the Soret band using CAM-B3LYP (TD-DFT) for all the crosslink models in various spin states are given in the ESI (see Table S6†). The table lists excitation energies along with oscillator strengths indicating the intensity of the transition. We highlight here that although several excited states may comprise the Soret band, for the purposes of comparing the spectral shifts with experiment, we focused on states with the highest oscillator strengths. The CAM-B3LYP functional was chosen because it successfully described the spectra of haem systems in previous studies.^65^ The lowest energy spin state for chain A for all the ferrous models is a quintet (*M* = 5). The quintet Ferrous-SC and SimFerrous-SC show peak maxima at 387 nm and 392 nm, respectively (see Table S6 in ESI† for excitation energies). The Ferrous-DC and SimFerrous-DC forms show similar absorption maxima, at 392 and 398 nm respectively. On introduction of the double bond in the DC models, we observe a red shift in the computed bands. The Ferrous-DCu and SimFerrous-DCu, show peak maxima at 411 nm. These peaks correspond to π–π* transitions within the haem moiety (see Fig. S20 in the ESI† for orbitals for DCu). All forms show a systematic overestimation of the excitation energies compared to the experimental absorption maximum of 460 nm observed for the ferrous form in solution. Such overestimation by TD-DFT is a known problem.^66^

For the ferric form, the lowest energy spin state for Ferric-DC and Ferric-SC is a sextet, while for Ferric-DCu it is a doublet (*M* = 2). The absorption maximum computed for Ferric-SC is 363 nm, while the corresponding value for Ferric-DC (*M* = 6) is 378 nm. The doublet Ferric-DCu exhibits a maximum at 368 nm.

The experimental observed spectral shift between the ferric and ferrous forms in solution is 0.27 eV. Spectral shifts in the same direction are seen between the ferric and ferrous forms for each of the alternative crosslink models, as shown in Fig. 5. The computed shift from Ferric-DCu is 0.35 eV for both the SimFerrous-DCu and Ferrous-DCu models, in the right direction. For the DC models, the sextet ferric form shows a spectral shift of 0.11 eV for ferrous and 0.16 eV for SimFerrous models (see Fig. S21 in the ESI†). As discussed earlier, the energy difference between the sextet and the doublet for Ferric-DC is ∼1 kcal mol^−1^ (Table S3A in ESI†), making both spin states accessible. Comparing the doublet Ferric-DC (maximum at 367 nm) with the ferrous and SimFerrous forms gives the right direction of the shift, computed as 0.21 eV for the ferrous form and 0.25 eV for the SimFerrous form (Fig. 5). For the Ferrous-SC and SimFerrous-SC models, we calculate a spectral shift of 0.21 eV and 0.25 eV respectively for the sextet ferric form. Changing the spin state for the ferrous and ferric forms has a smaller effect on the computed spectral shifts (Fig. S22–S24†).

Noting the experimental evidence above for a double crosslink in the ferric crystal and single crosslink in the ferrous form, the spectral shift was also calculated for the corresponding computational models. For the lowest energy spin states in Ferric-DCu and Ferrous-SC, the calculated TD-DFT absorption maxima are 368 nm (3.37 eV) and 387 nm (3.20 eV) respectively, a calculated shift of 0.17 eV, in reasonable agreement with the experimentally observed shift.

To analyse the origin of the Soret peak, the electronic transitions computed for the DCu form were analysed in terms of natural transition orbitals (NTOs) (Fig. S20 in the ESI†). The NTOs represent the electronic transitions in terms of holes and particles, giving a compact picture for the excitations involved. The computed NTOs for the Soret peak observed for the Ferric-DCu form indicate a localised excitation involving all the four rings of the haem. We observe localisation of the densities on the double bond in Lys78, providing evidence that the double bond is involved in the electronic transitions.

For comparison, excitation energies were also computed using simplified TD-DFT for various spin states with CAM-B3LYP, and with ZINDO/S for the lowest spin states (for sTD-DFT see Fig. S25–S29 and Table S7† and for ZINDO/S Fig. S30 and Table S8†). The quintet ferrous and SimFerrous-SC forms have sTD-DFT computed maxima at 415 nm and 425 nm, respectively. The maxima for quintet Ferrous-DC and SimFerrous-DC are at 457 nm and 450 nm respectively, while the corresponding values for Ferrous-DCu and SimFerrous-DCu are 457 nm and 454 nm respectively. For the Ferric-DC (*M* = 6) and Ferric-DCu (*M* = 2) forms, the corresponding values are 404 nm and 392 nm respectively. Comparing Ferrous-DCu and SimFerrous-DCu with that of Ferric-DCu, the predicted shifts are 0.44 eV and 0.43 eV, respectively, in the same direction but larger than observed experimentally. The corresponding values for the DC models are 0.54 eV and 0.50 eV respectively. However, the spectral shift calculated using sTD-DFT between Ferric-DCu and Ferrous-SC, in line with experimental evidence, is 0.17 eV and consistent with the shift computed using TD-DFT, providing further confidence in our calculations.

## Discussion

We combined multiple crystallographic and spectroscopic methods with state-of-the-art computational molecular simulations to elucidate the interaction between haem and protein in cytochrome P460 in the ferric and ferrous oxidation states. These results reveal the presence of a novel double crosslink between the active site lysine and the haem.

### New crosslink observed experimentally in ferric structures and comparison with simulations

Our previously published structure of McP460 at lower resolution (PDB 6HIU) showed a close contact between haem and the δ-carbon (CD) of the crosslinking lysine. We suspected this to be a sign of a more complex modification than the single crosslink between the lysine nitrogen (NZ) and γ-*meso* carbon (CHA) of the haem found in NeP460. The electron density for the lysine in 6HIU was difficult to assign to a particular chemical description, probably due to multiple conformations. To ascertain whether the additional conformation was the result of radiation damage, to which metalloproteins are particularly susceptible, we here obtained a high-resolution structure of the true ground state of the protein free of experimental artefacts by serial femtosecond crystallography (SFX) at 1.28 Å resolution. By using an X-ray free-electron laser (XFEL) with 10 fs X-ray pulses, we avoid electronic state changes from exposure to the X-ray beam, because the X-ray pulse is too short for radiation-induced chemistry to occur. This structure is one of the highest resolutions yet achieved at room temperature using SFX, and the high resolution provides confidence of an additional bond between the lysine and the haem. Intriguingly, even after ruling out effects of radiation damage, the structure still exhibited multiple conformations of the lysine, making the chemical nature of its crosslink unclear. In contrast, a room-temperature synchrotron structure from fresh crystals revealed a single conformation of the lysine with two crosslinks to the haem. This was not altered by crystal ageing, suggesting that it is not crystal size or age that determines the crosslinking, but rather that this is an inherent property of the crystalline protein. It is unclear what caused the variation in crosslinking between the SFX and previously published McP460 structure, both with multiple conformations of Lys78, *versus* the RT fresh and aged structures that exhibit only a double crosslink.

We suggest that the formation of the second crosslink also shifts the adjacent tetrapyrrole double bond to become exocyclic, based on its length and angle. We speculate that the crosslinking lysine sidechain is oxidised to form a double bond on it, which provides the reactivity to form a second crosslink in an interesting case of C–H activation. Based on the electron density in an omit map of the RT fresh structure, we suggest that the final structure still harbours a double bond on the lysine. These modifications to the haem and lysine are likely to be self-catalysed, because they are observed in recombinant McP460 expressed in *E. coli*. Notably, the single crosslink in recombinantly expressed NeP460 is also present again suggesting self-catalysed reactions.

QM/MM optimisations of the Ferric-SC/DC/DCu models revealed that all of the chemical structure alternatives are stable. Differences between the crystal models and QM/MM optimised structures are minimal (Table 2). The Fe-OOP distance, an indicator of the oxidation state of Fe, for the DCu structure closely matches the ferric SFX structure. Geometrical changes between spin states were also investigated by simulations. There are notable structural changes, *e.g.* the Fe–water and Fe–pyrrole N distances increase with increasing multiplicity, particularly for DC and DCu systems (almost 0.1 Å increase in the Fe–N distance of the pyrrole D ring in both chains). Elongation of one Fe–N distance is also observed in RT fresh and aged experimental structures, albeit to a lesser extent (less than 0.1 Å). Although the lowest energy spin state for DCu is *M* = 2, it is energetically very close to *M* = 6 (energy difference = 2.7 kcal mol^−1^). The active site structures of the DC and DCu are very similar for all the spin states (Table 2 and ESI Table S4A†).

The differences between the two chains of this dimeric protein in QM/MM optimisations are probably related to alternative conformations of the active site residue Asp102. This alteration of Asp102 conformation perturbs the water distribution around the haem Fe, as shown by MD with the protein held at its crystallographic positions (Fig. S16†). When the sidechains were relaxed, allowing this Asp102 to adapt to other conformations, the distribution and interaction of water near Fe becomes equivalent in the two chains (Fig. S17†). Optimisation of the DC system without the environment gave the same lowest energy spin state, *M* = 4 (ESI Table S5†). In transition metal complexes, multiple spin states may be involved in reactions, and can interconvert through spin crossovers, known as multiple-state reactivity.^67^ The calculated lowest energy spin states for SC/DC/DCu differ between the two chains, but the energy differences between the spin states are small (∼4.0 kcal mol^−1^, ESI Table S3A† last column), indicating the possibility of multi-state reactivity.

The conformation of Arg50 seen in the three structures determined at RT (conformation B in chain A) could affect haem ruffling and crosslink formation through its decreased interaction with the haem propionate oxygen OD2 relative to the conformations previously observed.^68^ Asp102 in McP460 is probably involved in a proton relay, like the equivalent Glu96 in NeP460.^68^ In the previously published 100 K structure of McP460, Asp102 was further from the iron centre (average 5.7 Å from CG to Fe) than in the SFX structure (average 5.5 Å), which is probably more physiologically relevant due to the absence of radiation damage and cryogenic artefacts. The RT fresh structure is in between (average 5.6 Å). The differences could be due to a lengthening of the iron–water bond with radiation damage,^64^ with RT fresh being a relatively low-dose structure (32 kGy). In the ferrous cryo structure, Asp102 is the furthest from the iron centre (average 5.9 Å), consistent with proton transfer not being needed at this stage of the catalytic cycle, because no ligand is bound to the iron. Proton transfer has been suggested to be prevented when the carboxylate group of Asp102 is too distant to participate in the proton relay (*e.g.* Fe–CG distance of 6.4 Å in the crosslink-deficient inactive mutant of NeP460).^68^

### Comparison of experimental ferrous cryo structure and its simulated counterpart

Obtaining a crystal structure of the ferrous form of cytochrome P460 proved highly challenging, whether using chemical reduction or *via* electrons generated through X-ray exposure. While crystals change colour in a manner consistent with reduction by sodium dithionite, they are extremely prone to reoxidation under aerobic conditions. In contrast, under anaerobic conditions it was possible to determine a structure from a crystal with a corresponding single-crystal spectrum consistent with the ferrous enzyme. In the crystal structure, the haem distal binding position was vacant, consistent with loss of water molecule upon reduction. This results in 5-coordination of Fe(ii), which therefore moves 0.2 Å towards the proximal side of the plane of the haem. This is clearly different to the 6-coordination of Fe by water in the SFX and RT fresh structures, giving confidence that they are indeed in the ferric state. The second crosslink (Lys CD–haem C2A) is broken in the crystallographic ferrous structure, causing the terminal (CMA) carbon of pyrrole ring D to change from methylene (–CH_2_) to methyl (–CH_3_).

Simulations were carried out with the ferrous cryo structure following the same protocol as used for the Ferric-SC system. The reduced X-ray structure shows a long CD–C2A distance, consistent with an SC structure. As for the ferric state, QM/MM optimisation resulted in lowest energy for the quintet spin state. The haem–water distance was 3.26 Å and is in good agreement with that observed for ferrous cryo (3.42 Å). The Fe-OOP distance also showed good agreement, 0.40 Å for the optimised structure compared to 0.35 Å observed experimentally. The increased Fe–water distance is also reflected in MD, with the average distance 3.3 ± 0.1 Å and 3.4 ± 0.1 Å in chains A and B, respectively. This loss of water from haem coordination results in the displacement of the water towards the pyrrole rings and formation of H-bonds with the N of the pyrrole rings, behaviour not observed in the oxidised state (see Fig. S18B(I)†).

### Double crosslink models based on the ferrous cryo structure

For comparison, we also modelled DC and DCu starting from the ferrous cryo structure (Ferrous-DC/DCu), and compared optimised geometries and simulated UV-vis spectra (geometries in ESI (Fig. S19(I)†) and geometrical parameters in Table 2). The geometrical parameters are quite similar to the simulated DC and DCu reduced state forms generated from Ferric-SFX, except with longer Fe–HisN distances, shorter Fe–water distances and shorter Fe-OOP in SimFerrous-DC/DCu than Ferrous-DC/DCu.

### Comparing ferrous cryo and *in silico* reduced models

Our studies provide the opportunity to compare whether an *in silico* reduced model (SimFerrous-SC) from an oxidised structure (ferric SFX) gives results similar to simulations starting from the experimental reduced structure (Ferrous-SC).

SimFerrous-SC systems do show similar lengthening of the Fe–water distance and increase of the Fe-OOP distance, as in the ferrous cryo structure. MD shows more variations in the SimFerrous-SC system, due to the asymmetry in the two chains, and the trajectories reveal that Fe loses water coordination from the 6th coordination site for both systems. This water in turn forms H-bonds with pyrrole NA, more in chain A, in both systems. A similar pattern is observed among the two chains when H-bond interaction with Asp102 and the water is considered.

### Evidence from calculations of the UV-vis spectra

Spectral calculations revealed the computed spectral shifts between absorption maxima are relatively insensitive to the spin state of the ferrous and ferric forms. The spectral shift observed experimentally is qualitatively reproduced irrespective of changes in the spin state on reduction and the chemical structure of the crosslink. Although there is a complex relationship between geometry, spin multiplicity and energetics, a good level of agreement with experiment is seen for the calculate shift between the DCu model in the ferric form and SC model in the ferrous form, in line with the assignments in the experimental structures.

There are differences in the spin energetics for chain A and chain B arising due to the conformation of Asp102, the energy differences between the spin states are small (<∼4.0 kcal mol^−1^). These calculations altogether provide evidence for a double crosslinked species in the crystal. We do not observe a change in the calculated spectra across spin states, indicating spin independence of the spectra.

The double crosslink we observe in McP460 is reminiscent of that seen in the P460 haem from HAO.^69^ HAO also harbours a crosslink between the meso carbon of its haem (albeit the opposite one, α-meso a.k.a CHC) and an amino acid sidechain, though from a tyrosine residue. A second crosslink was observed between the tyrosine sidechain oxygen and the pyrrole carbon next to the α-*meso* carbon, forming a new 5-membered ring^70^ (as opposed to the herein reported 6-membered-ring). However, the π bond in the HAO 5-membered ring is unlikely to be part of the haem π-system, as it is orthogonal to it. On the other hand, the double bond on the lysine of McP460 is involved in the electronic transition of the Soret peak (indicated by natural transition orbitals, Fig. S20 in the ESI†). The difference between the Soret bands of McP460 (∼420 nm) and NeP460 (440 nm)^71^ was previously attributed to the iron-bound water of McP460.^6^ The second lysine crosslink of McP460 is likely to be a bigger factor.

## Conclusions

We describe the first serial femtosecond crystallography structure of any cytochrome P460, achieving the third highest resolution room temperature structure from an XFEL in the PDB at the time of writing. The structure of ferric McP460 is free of the effects of radiation induced chemistry, due to the principle of diffraction before destruction, and avoids the presence of artefacts due to cryocooling. This structure suggests a second cross link between the active site lysine and the haem, and room temperature multi-crystal structures support this, excluding the possibility that it could be caused by ageing of crystals. An anaerobic, freeze-trapped structure of the ferrous form of the enzyme was also determined from a single-crystal with the oxidation state confirmed using single-crystal UV-visible spectroscopy.

QM/MM simulations of the ferric form of McP460 indicate that a double crosslink structure is stable and compatible with the experimental data. Distortion of the haem between Fe and pyrrole D ring nitrogen is observed in the optimised structures, in qualitative agreement with the crystal structure. QM/MM geometry optimisations of the ferrous form also reproduced experimental structural features such as the interaction of water and iron at the 6th coordination site. QM/MM calculations of the oxidised and reduced forms revealed a complex energetic landscape, with several spin states accessible in both, and sensitive to the local environment of the haem. UV-vis spectroscopic calculations using TD-DFT suggest that the shift in absorption maximum observed experimentally between the ferric and ferrous forms is compatible with and supports a double crosslink model for the ferric form. We also considered a model reduced *in silico*, starting from the ferric crystal structure. This reproduced key features of the experimental data, indicating that reduction *in silico* is a valid approach to modelling the ferrous state, adding further support to the double-crosslink proposal.

Intriguingly, no evidence of a second cross link was observed in previous crystal structures^5^ of a different cytochrome P460 from an ammonia-oxidising organism, which may relate to differing catalytic activity and chemical conditions of the organisms. Notably, NeP460 contained a hydroxyl modification to the haem group evident in low-dose structures that disappeared in higher dose structures, a feature not present in McP460.

Our work highlights the power of combining multiple experimental and computational techniques to address complex structural questions. Electron density features that may be ambiguous or unclear even at very high resolutions can be investigated by simulation. The simulations benefit from starting structures free of experimental artefacts such as radiation damage and changes due to cryocooling. Comparison of predicted structures with crystallographic models allows for further cross-validation. Future work will focus on applying this combined approach to ligand-bound states of cytochrome P460, site-directed mutagenesis to probe the catalytic mechanism^16^ and analysing the effects of the novel crosslinking on it. We will also investigate the intriguing question of the mechanism of formation of the second crosslink.

## Author contributions

Experimental investigations and data analysis: H. E. P., H. R. A., M. A. H., M. E., with support from facilities scientists S. J., T. T., H. S., S. H., J. B.-E., R. L. O. and in collaboration with C. R. A., J. A. R. W. and I. T. Computational investigations and data analysis: K. S., A. G. R., Y. L., C. Y., A. J. M., T. W. K. Software development: Y. L., C. Y., K. S., T. W. K. Visualisations: H. E. P., K. S. and A. G. R. Research design: M. A. H., A. J. M., T. W. K. Funding acquisition: T. W. K., M. A. H., A. J. M., M. E., C. Y., Y. L. and K. S. Writing – original draft: H. E. P., A. G. R. and K. S. Writing – review and editing: M. A. H., A. J. M. and T. W. K., with additional reviewing and approval from all the authors.

## Conflicts of interest

There are no conflicts to declare.

## Supplementary Material

SC-016-D5SC04213E-s001

SC-016-D5SC04213E-s002

## Data Availability

Coordinates and data for crystal structures have been deposited at the Protein Data Bank under accession numbers shown in Table 1. The Python-based version of the ChemShell software package used for our QM/MM calculations (version 23.0) is available free of charge under an open source license at https://chemshell.org/. Further experimental and computational data supporting this article have been included in the ESI document,† together with a set of QM/MM optimised structures in an accompanying compressed folder.
