# Supplementary material for: Double crossed? Structural and computational studies of an unusually crosslinked haem in Methylococcus capsulatus cytochrome P460
Source: Chem Sci. 2025 Aug 8;16(35):16266–83. doi: 10.1039/d5sc04213e (PMC12352625; doi:10.1039/d5sc04213e)
Supplement: SC-016-D5SC04213E-s001 [file SC-016-D5SC04213E-s001.pdf]

Supplementary Information (SI) for Chemical Science.
This journal is © The Royal Society of Chemistry 2025

Supporting Information

Double crossed? Structural and computational studies of an unusually crosslinked haem in *Methylococcus capsulatus* cytochrome P460

Hans E. Pfalzgraf,§[a,b] Aditya G. Rao,§[c], Kakali Sen,§[d] Hannah R. Adams,[e] Marcus Edwards,[e] You Lu,[d] Chin Yong,[d] Sofia Jaho,[a,b] Takehiko Tosha,[f] Hiroshi Sugimoto,[g] Sam Horrell,[a,b] James Beilsten-Edmands,[a] Robin Owen, [a] Colin R. Andrew,[h] Jonathan A. R. Worrall,[e] Ivo Tews,[i] Adrian J. Mulholland,*[c] Michael A. Hough,*[a,b] and Thomas W. Keal*[d]

§ These authors contributed equally to this work: H.E.P., A.G.R., K.S.

* Corresponding authors: adrian.mulholland@bristol.ac.uk; michael.hough@diamond.ac.uk; thomas.keal@stfc.ac.uk

a Diamond Light Source Ltd., Harwell Science and Innovation Campus, Didcot, OX11 0DE, UK

b Research Complex at Harwell, Rutherford Appleton Laboratory, Didcot, OX11 0FA, UK

c Centre for Computational Chemistry, School of Chemistry, University of Bristol, Bristol BS8 1TS, UK

d STFC Scientific Computing, Daresbury Laboratory, Keckwick Lane, Daresbury, Warrington, WA4 4AD, UK

e School of Life Sciences, University of Essex, Wivenhoe Park, Colchester, Essex, CO4 3SQ, UK

f Graduate School of Science, University of Hyogo, Hyogo, Japan

g RIKEN SPring-8 Center, 1-1-1 Kouto, Sayo, Hyogo, 679-5148, Japan

h Department of Chemistry and Biochemistry, Eastern Oregon University, La Grande, Oregon, USA

i Biological Sciences, Institute for Life Sciences, University of Southampton, Southampton SO17 1BJ, UK

Table of Contents

		Page
1.	Crystals of ferric enzyme	3-4
2.	Crystals of ferrous enzyme	5
3.	Modelling haem atomic charges and crosslink structures	6-10
4.	Activity assay for oxidation of hydroxylamine	11
5.	Haem site parameters for structures of McP460, reported here and published earlier along with published NeP460	12
6.	Alignment and fitting of ferric structures	13-15
7.	Structural and spectroscopic data obtained in solution and crystal	14, 15
8.	Overlay of ferrous cryo with previous published ferric cryo structures	16
9.	Energies and structural parameters for all QM/MM optimised structures	17-21
10.	MD analysis of ferric, ferrous and *SimFerrous* enzyme systems	22-25
11.	Comparison of QM/MM optimized Ferrous and *SimFerrous* structures	26
12.	Computed excitation energies and oscillator strengths and simulated spectra of all optimised crosslinks in ferric and ferrous forms in all the respective spin states	27-34
13.	Haem restraints used in the determination of the crystallographic structures reported in this work	35
	References	71

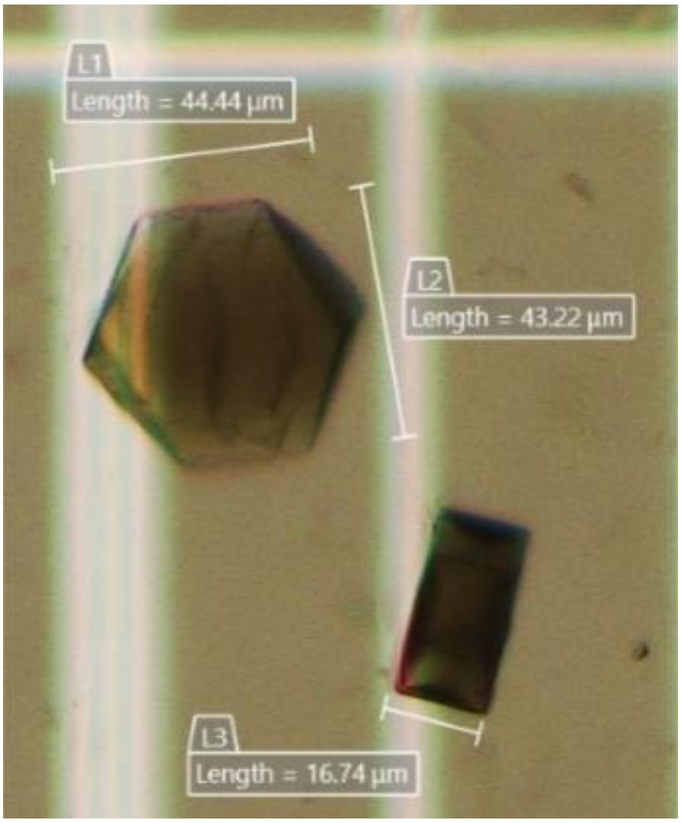

Fig. S1. Batch crystals from SFX experiments. L1 for length, L2 for width and L3 for thickness

A)

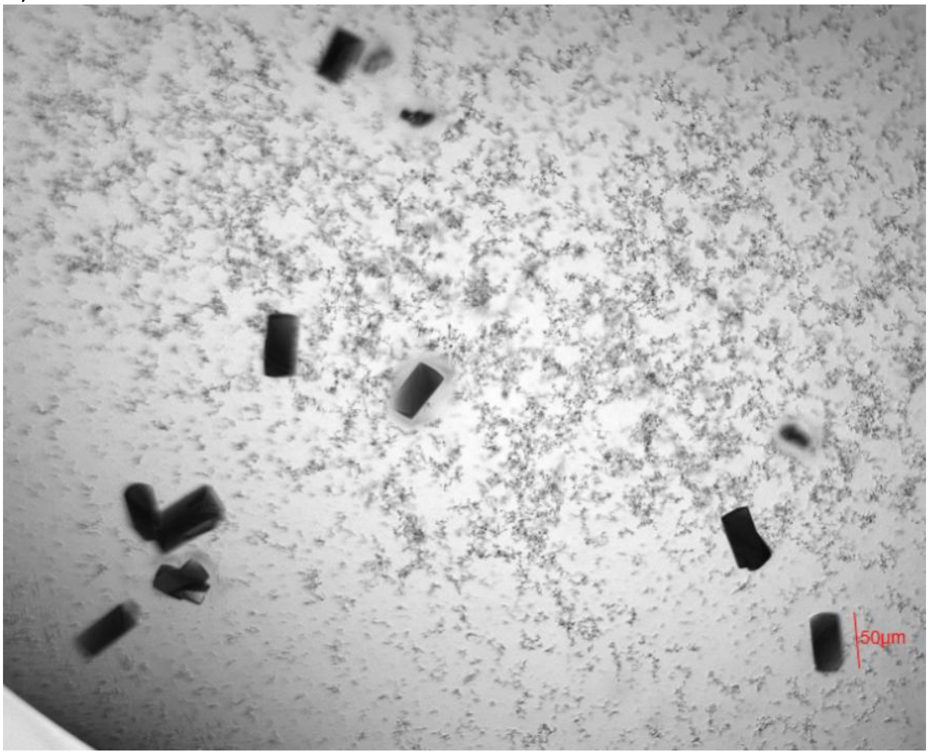

B)

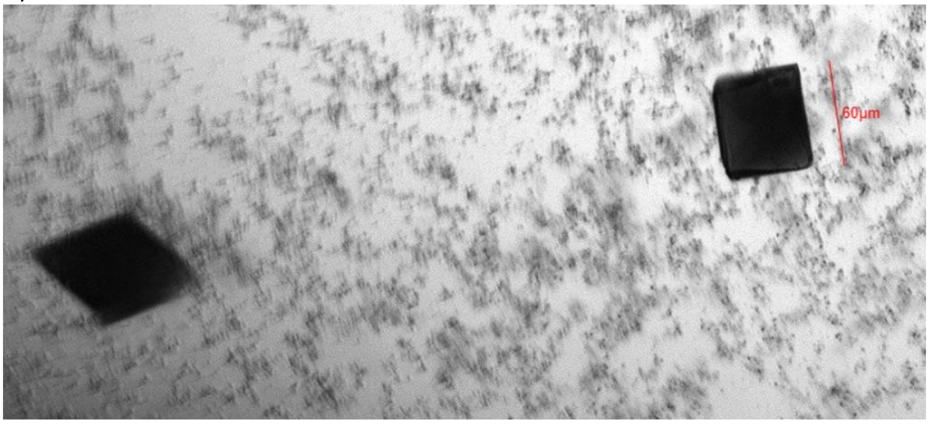

Fig. S2. Crystals used for A) RT fresh and B) RT aged structures

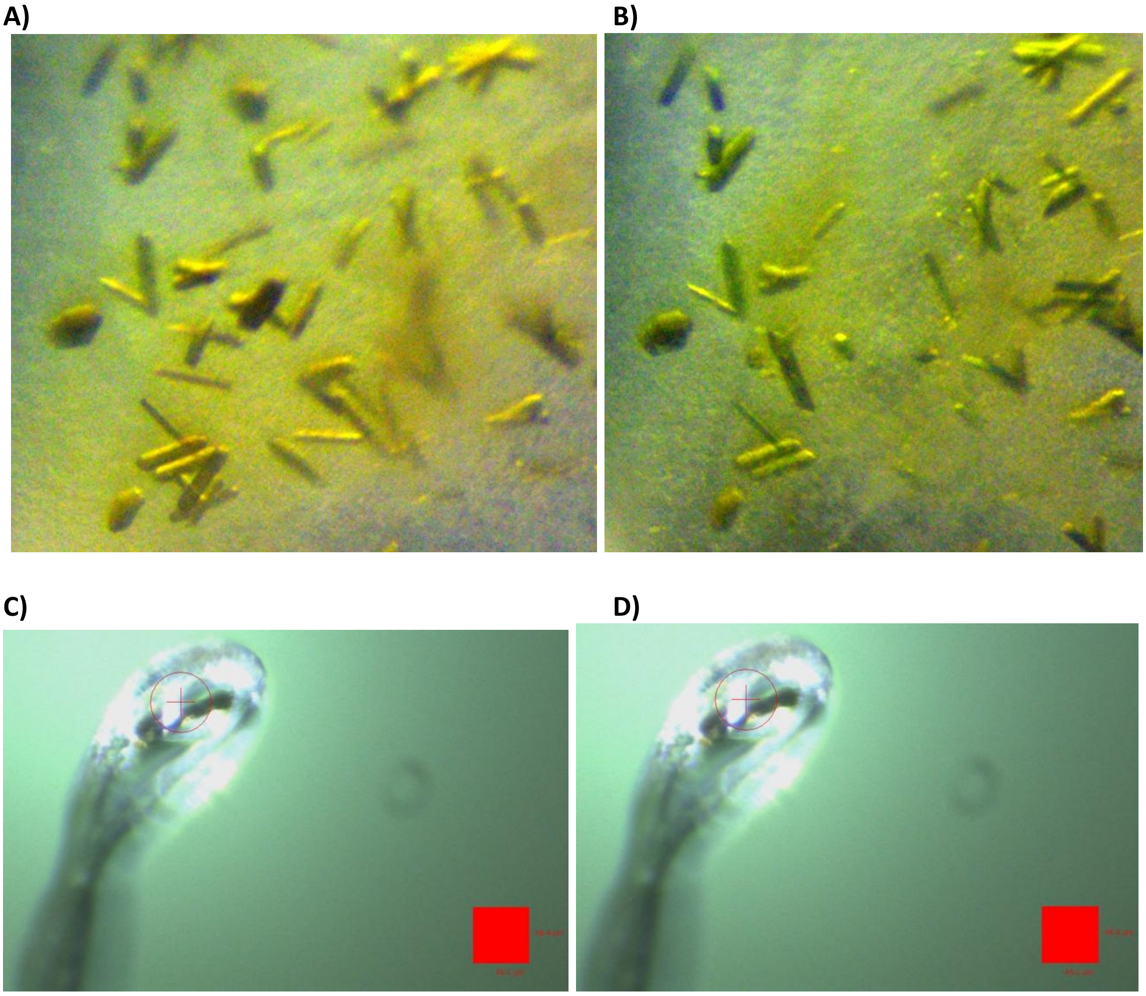

Fig. S3. McP460 crystals (200×35×20 µm) in cryoprotectant A) before and B) after addition of sodium dithionite reductant. C) Cryocooled crystal before and D) after collection of the first X-ray diffraction dataset (illuminated by the white light of the microspectrophotometer)

S.1 Charge determination

P460 has a unique haem C unit with an additional covalent bond between the NZ atom of Lys78 and meso-CG (CHA) of haem C (Scheme 1, main manuscript). Modelling this covalent modification required calculation of the atomic charges of the haem C unit with this unique covalent bond and the coordinated residues. CHARMM forcefields (FFs) use groups to define neutral units within molecules. For the available haem B cofactor all the side chains (propionate, vinyl and methyl) of Haem B unit are defined as groups and sums up to 0.0. To minimize the charge manipulation and fit it with the rest of the charges provided by CHARMM FF (Version 36)[1,2] the following steps were performed: for the charge calculation of the modified haem C cofactor in P460, a cluster model consisting of the core haem C, the covalently linked Cys140 and Cys143, the proximal His ligand and the crosslinked Lys78 was considered. All these amino acid residues were truncated at the CA-CB (Fig. S4. The brown stars indicate where the side chains of the haem were cut off, and the purple stars indicate the terminal atoms of the linked active site residues.) Geometry optimization was carried out in NWChem,[3] and the CB atoms of His144, Cys140, Cys143 were fixed to their crystallographic coordinates. For Lys78, to keep as close as possible to the crystal structure, CB and CG were fixed. The DFT functional B3LYP[4,5], with D3 dispersion correction[6], was used[7]. The basis set 6-31G*[8,9] was used for all atoms. The geometries were optimized in the spin states doublet (M = 2), quartet (M = 4) and sextet (M = 6) for the ferric state and singlet (M = 1), triplet (M = 3), and quintet (M = 5) for the ferric state, respectively. The electrostatic potential (ESP) scheme within NWChem was used to derive charges. The lowest energy optimized state was taken for charge determination: this was the quartet for ferric and triplet for ferrous states, respectively. Constraints were applied to the ESP charges to enforce equivalency of chemically similar groups and also to minimize disparity with the CHARMM FF charges when this cofactor is added within the protein for simulation. The constraints used were:

1. Charges were set to zero for the H atoms that were used to replace the side chains of haem (Figure S1, brown stars)
2. Charges were set to zero for the H atoms that were added to replace the cut made between CA-CB atoms for the amino acids (pink stars)
3. Additionally, the charges from CB to CD were summed to zero for the Lys78 side chain (pink stars) in line with the standard CHARMM FF (Version 36) for this amino acid.
4. In haem C, Cys covalently links to the vinyl (-C=CH_2) group of haem B forming -CH(SCys)-CH_3. The charges on this generated -CH_3 group were summed to zero (pink stars) and the charge on H-atoms were made equivalent.
5. The charges on the His residue were also summed to zero, following the His links to Haem B groups in the CHARMM FF which remains unaffected.
6. All equivalent atoms were required to have equal charges.
7. For the porphyrin core, the two pyrrole rings next to the methylene bridge (CHA), where Lys78 forms the cross link were kept equivalent to one another. The other two pyrroles were also kept equivalent to each other.
8. Except for the methylene bridge (CHA) where the Lys cross links, the other 3 were made equivalent.
9. The double crosslink (DC) and double crosslink with unsaturated Lys (DCu) values were generated by removing the corresponding H-atoms and adding the charges of the removed H- atoms to the C-atom it was bonded to.

The charges so derived are provided in Table S1. The charges that differ in the cases of DC and DCu are given in in Table S1.

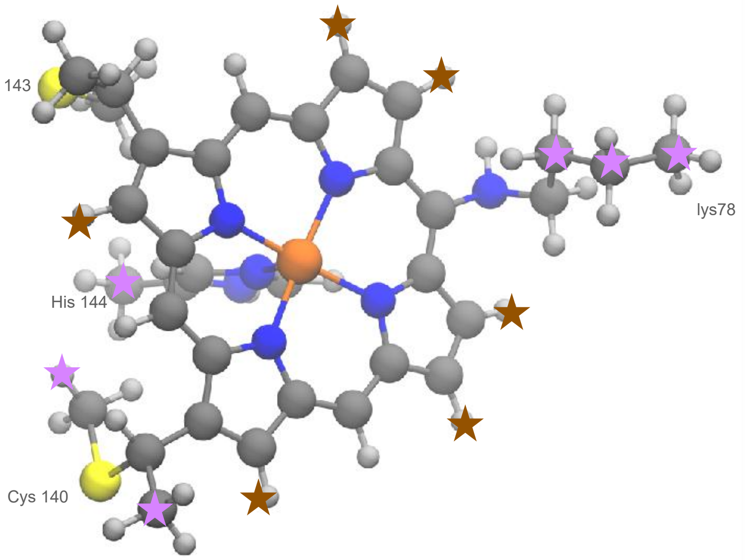

Fig. S4. The cluster model used for charge determination. The brown stars indicate where the side chains of the haem were cut off and the purple stars indicate the terminal atoms of the linked active site residues.

Table S1. MM atomic charges (units of electron charge) used in MD for the ferric and ferrous states for the haem C unit with the covalent links to Lys78, Cys140 and Cys143 and coordinated His residue. The full set of charges for the single crosslink (SC) models are provided. For the double crosslink (DC) and double cross link with unsaturated Lys (DCu) models, only the atoms that had different charges are listed.

Residue	Atom names	Single crosslink (SC)		Double crosslink (DC)		Double crosslink with unsaturated Lys (DCu)	
		Ferric	Ferrous	Ferric	Ferrous	Ferric	Ferrous
Lys-78	CD*	NC	NC	-0.09	-0.09	0.00	0.00
	HD	NC	NC	0.09	0.09	-	-
	CE*	0.08	0.16			0.23	0.24
	HE1	0.15	0.08			0.15	0.08
	HE2	0.15	0.08			-	-
	NZ	-0.85	-0.83				
	HZ	0.40	0.41				
Cys-140	CB	0.16	0.23				
	HB1	-0.05	-0.07				
	HB2	-0.05	-0.07				
	SG	-0.29	-0.37				
Cys-143	CB	0.16	0.23				
	HB1	-0.05	-0.07				
	HB2	-0.05	-0.07				
	SG	-0.29	-0.37				
His-144	CB	0.09	-0.04				
	HB1	0.06	0.08				

	HB2	0.06	0.08				
	ND1	-0.12	-0.14				
	HD1	0.31	0.28				
	CG	-0.10	0.02				
	CE1	-0.02	-0.02				
	HE1	0.18	0.16				
	NE2	-0.36	-0.28				
	CD2	0.18	-0.10				
	HD2	0.07	0.19				
Haem C	FE	0.51	0.20				
	NA	-0.20	-0.19				
	NB	-0.27	-0.10				
	NC	-0.27	-0.10				
	ND	-0.20	-0.19				
	C1A	0.24	0.26				
	C2A	-0.12	-0.17				
	C3A	-0.12	-0.17				
	C4A	0.24	0.26				
	C1B	0.34	0.18				
	C2B	-0.17	-0.16				
	C3B	-0.17	-0.16				
	C4B	0.34	0.18				
	C1C	0.34	0.18				
	C2C	-0.17	-0.16				
	C3C	-0.17	-0.16				
	C4C	0.34	0.18				
	C1D	0.24	0.26				
	C2D	-0.12	-0.17				
	C3D	-0.12	-0.17				
	C4D	0.24	0.26				
	CHA	0.28	0.16				
	CHB	-0.43	-0.36				
	HB	0.22	0.17				
	CHC	-0.43	-0.36				
	HC	0.22	0.17				
	CHD	-0.43	-0.36				
	HD	0.22	0.17				
	CMA*	NC	NC	-0.18	-0.18	-0.18	-0.18
	HMA1	NC	NC	0.09	0.09	0.09	0.09
	HMA2	NC	NC	0.09	0.09	0.09	0.09

Cys-140 link	CAB	0.52	0.61
	HAB	-0.08	-0.10
	CBB	-0.37	-0.39
	HBB1	0.11	0.09
	HBB2	0.11	0.09
	HBB3	0.11	0.09
Cys-143 link	CAC	0.52	0.61
	HAC	-0.08	-0.10
	CBC	-0.37	-0.39
	HBC1	0.11	0.09
	HBC2	0.11	0.09
	HBC3	0.11	0.09

NC: this group was not considered in the cluster for charge calculation; CHARMM FF charges were used directly. *DC and DCu involved removal of an H atom bonded to Haem-CMA and Lys-CD, so the charge on H atom that was removed was added to the charge of the C atom to which it was bonded. The change from DC to DCu involved an unsaturated Lys CD-CE bond, hence required removal of further H-atoms bonded to CD and CE of lysine. So, the charges of these H atoms removed were added to the remaining charge on the CE and CD atoms.

S.2 Modelling the crosslinks

To model the cross-link to represent both double and single crosslinks, partial optimization of the haem unit around the crosslink was performed. Gas-phase DFT cluster calculations were performed in which all the haem atoms, except those involved in the crosslink and their two immediate neighbouring atoms were fixed (Fig S5). Both oxidized and reduced forms of Fe were calculated; spin states considered were M=2, 4 and 6 for the ferric state and M=1, 3 and 5 for the ferrous state. The resulting optimised geometries from all spin states were similar for both oxidised ferric and reduced ferrous systems. The key distance: LysN-CHA (1.36 ± 0.02 Å) remains invariant among all the optimised structures for both SC and DC systems. In addition, the CD-C2A (1.55 Å) and C3A-CMA (1.35 Å) distances for DC are also invariant across all structures. The angles around the linking of NZ of Lys78 to haem averages to: NZ-CHA-C1A (115.69 ± 0.31^{o} and 116.60 ± 0.59 ^{o}, for SC and DC, respectively), NZ-CHA-C4D (120.58 ± 0.34^{o} and 121.86 ± 0.32^{o} for SC and DC, respectively). The angles around the second CD-C2A link: CD-C2A-C1A and CD-C2A-C3A for DC averages to 99.24 ± 0.46^{o} and 113.20 ± 0.20^{o}, respectively. The resulting optimised SC and DC haem C structures in the lowest spin state were aligned to the crystal haem C unit of the **ferric SFX** structure. The alignment showed good agreement at the junctions of CA – CB bond to Lys78, Cys140, Cys143 and His144. The haem unit from SFX structure was replaced by these optimised SC and DC haem units, thus providing starting structures for both SC and DC (Fig S6).

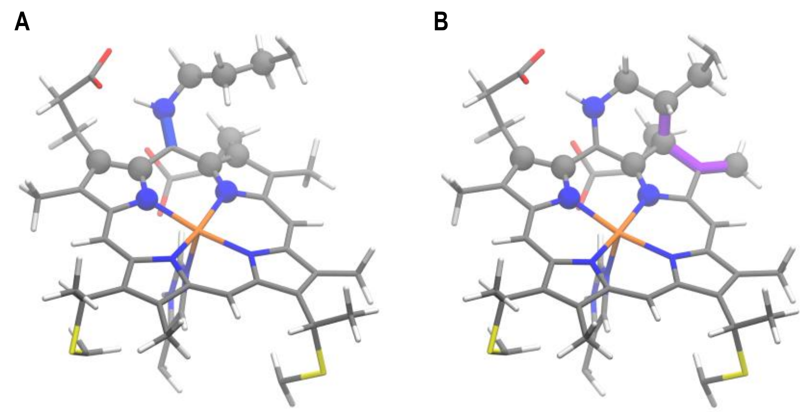

Fig. S5. QM models for generating A) SC and B) DC haem units. The atoms in spheres were optimized only, while the rest were fixed.

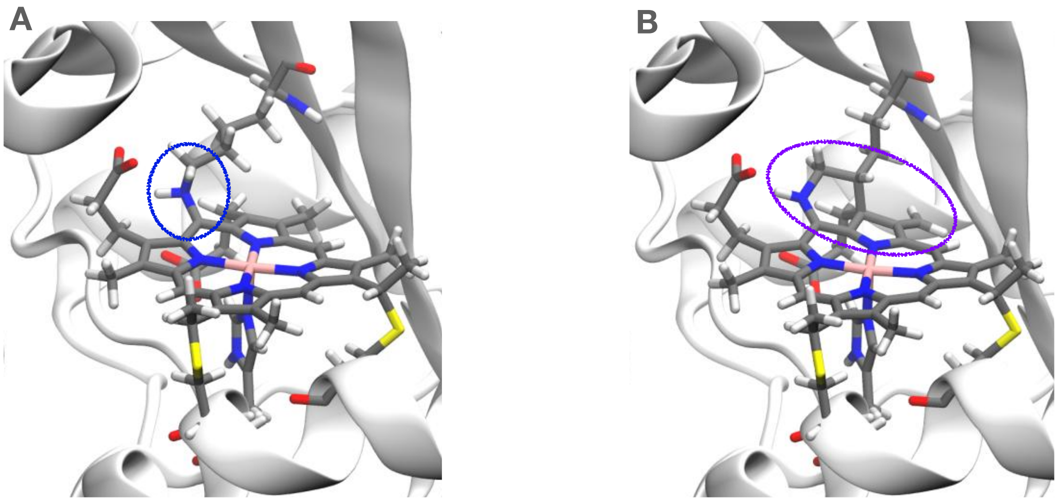

Fig. S6. Optimised QM models A) SC and B) DC haem units with crosslinks aligned within the active site of ferric SFX providing the initial structures for Ferric-SC and Ferric-DC.

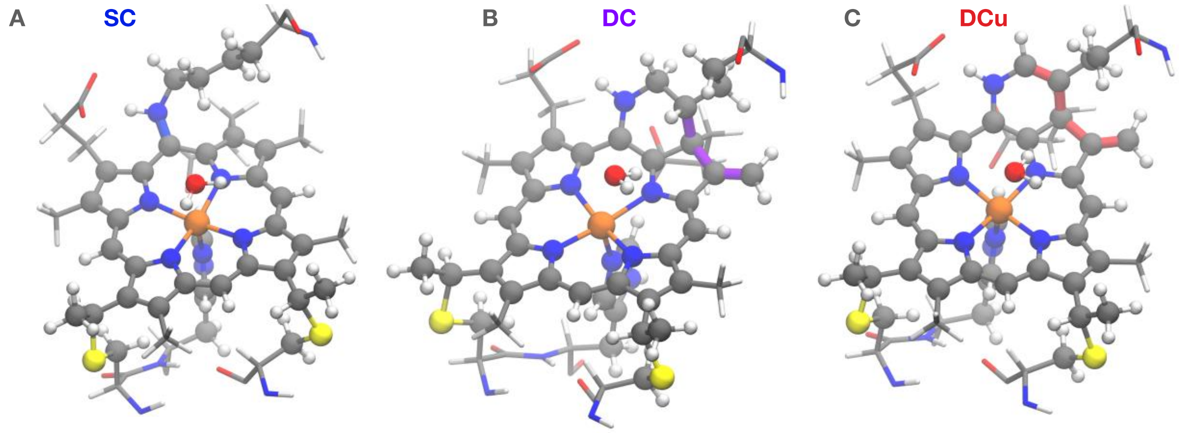

Fig. S7. Optimised models with QM region atoms shown in spheres for A) SC, B) DC and C) DCu. The changes in bonding due to crosslink are shown in blue for SC, in violet for DC and in red for DCu.

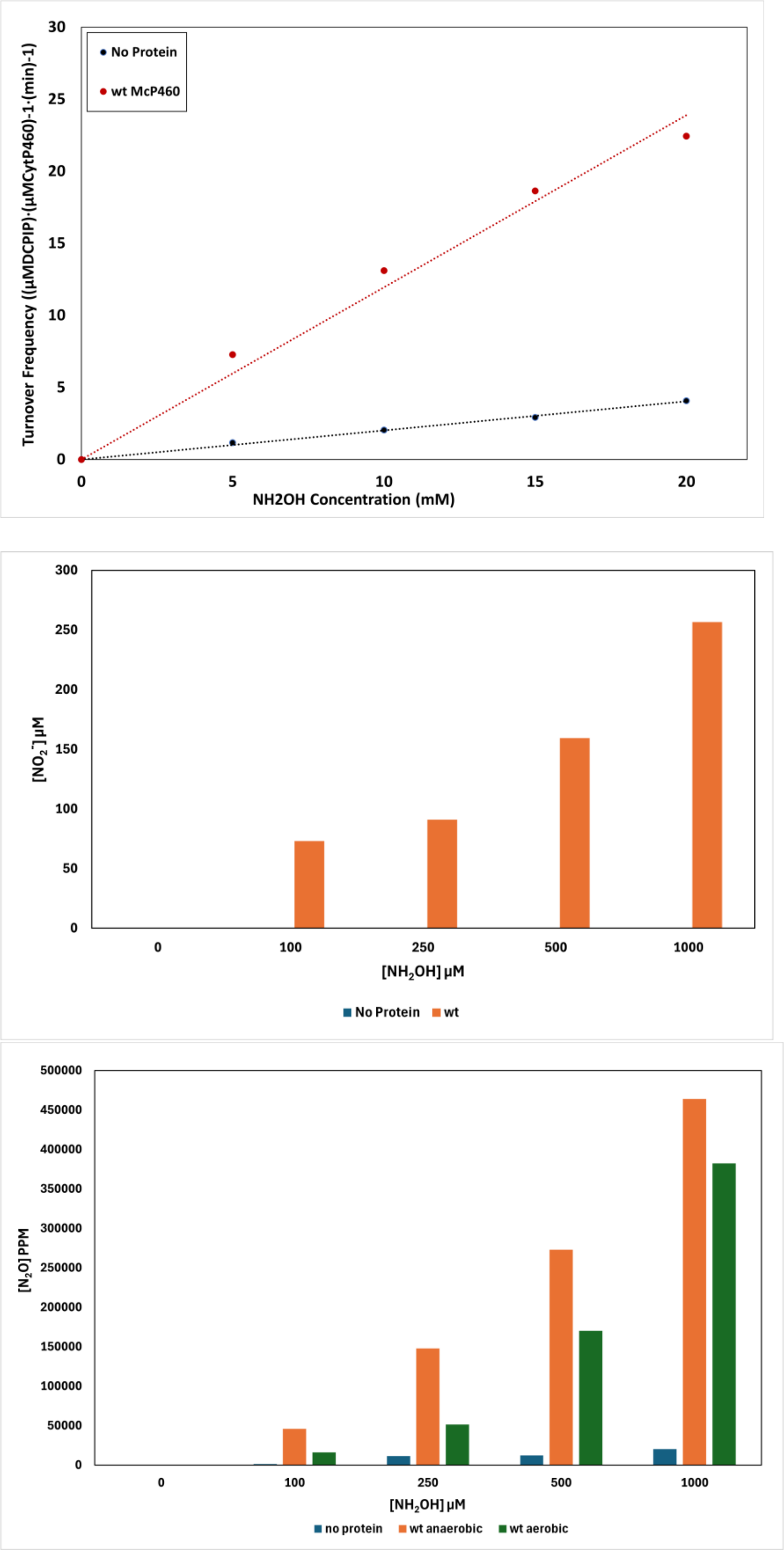

Fig. S8: Enzyme activity data for the oxidation of hydroxylamine by McP460 (top panel); production of nitrite (middle panel) under both aerobic and anaerobic conditions; production of nitrous oxide under aerobic and anaerobic conditions (bottom panel).

Table S2 - Haem site parameters for P460 crystal structures (chain A/chain B)

**Structure**	**Ferric SFX [9hs4]**	**VMXi 1 fresh [9hs9]**	**VMXi2 aged [9hs6]**	**Ferrous (100K) [9hrk]**	**6hiu (100K)**	**NeP460 (2je3)** (100K)
Resolution (Å)	1.28	1.66	1.77	1.33	1.36	1.80
Fe–His N (Å)	2.14/2.12	2.11/2.05	2.11/2.06	2.10/2.10	2.13/2.12	2.16
Fe–water (Å)	2.11/2.11	2.15/2.11	2.06/2.09	-3.33/3.20	2.32/2.37	2.62 to Pi
Fe-PyrNA (Å)	(2.03,1.99, 2.00,2.00) / (2.02,1.99, 2.01, 2.01)	(2.06, 2.00, 2.03,2.01) / (2.07,1.98, 2.04, 2.98)	(2.04,1.99, 2.04,2.02) / (2.09,2.01, 2.04,2.0)	(2.10,2.09, 2.06,2.12) / (2.10,2.05, 2.00,2.05)	(2.07,2.02,2 .07,2.04) / (2.08,2.01, 2.03,2.08)	2.12, 2.10, 2.12, 2.12
Lys–CHA (Å)	1.46/1.50	1.40/1.38	1.40/1.39	1.44/1.41	(1.35,1.35) / (1.33, 1.37)	1.63
LysCD-C2A	1.94/2.11	1.58/1.58	1.55/1.56	3.72/3.71	(2.17,3.92) / (2.26,3.83)	3.70
C3A-CMA	1.43/1.44	1.33/1.33	1.35/1.33	1.53/1.50	1.48/	1.55
Fe -OOP (Å)	0.06/0.06	0.11/0.13	0.08/0.06	0.35/0.33	0.11/0.10	0.24

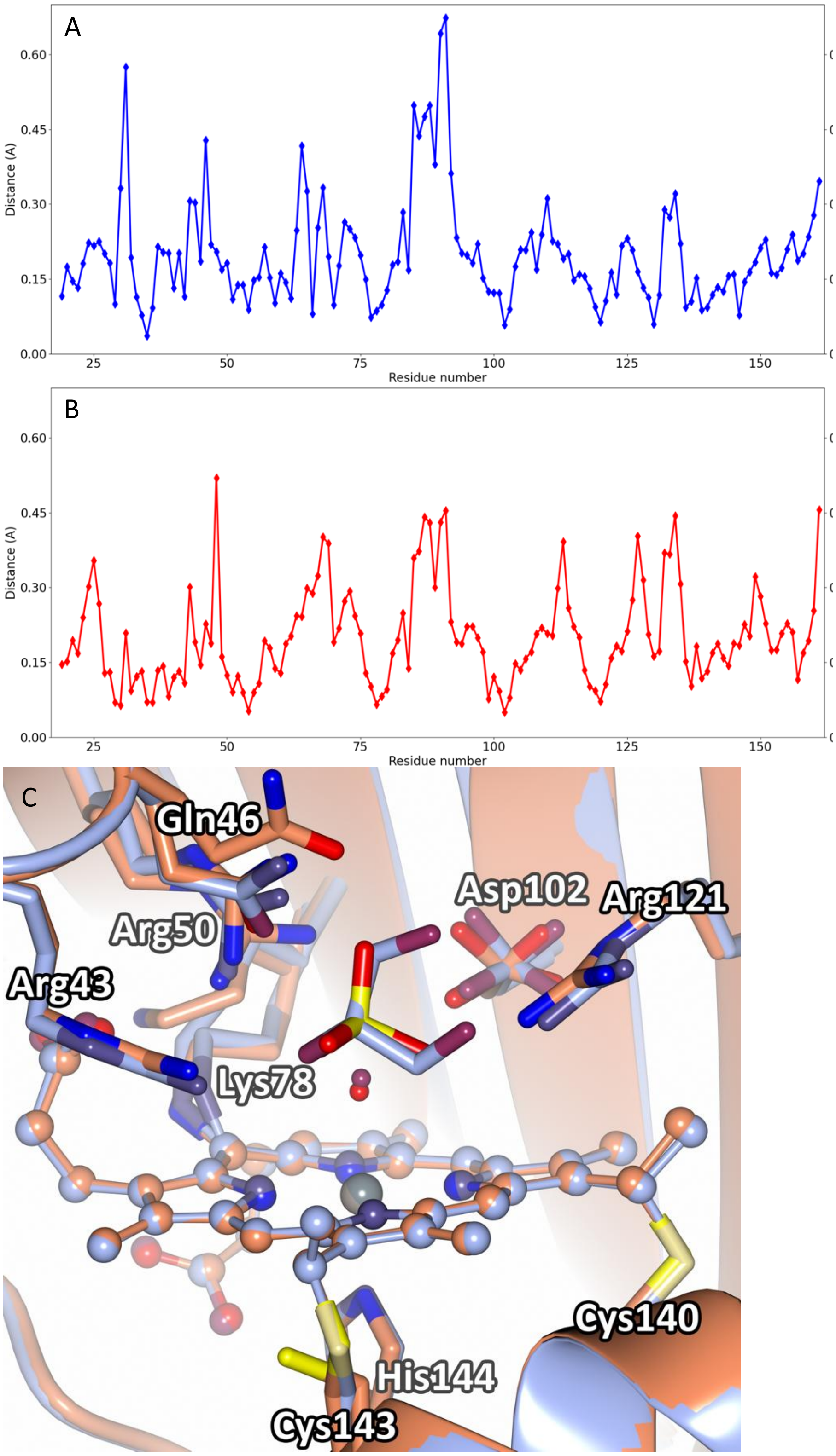

Fig. S9. A) RMSD between the Cα atoms of the previously published 100 K structure 6HIU and those of the **SFX** structure for chain A and **B)** for chain B based on their alignment using Gesamt. **C)** Comparison of the active sites in the previously published 100 K structure (PDB 6HIU, blue and cooler colours) and in the damage-free r.t SFX structure (orange and warmer colours). The sulphate (yellow and red) in the SFX structure is instead the site of a cryoprotectant molecule in the 100 K structure.

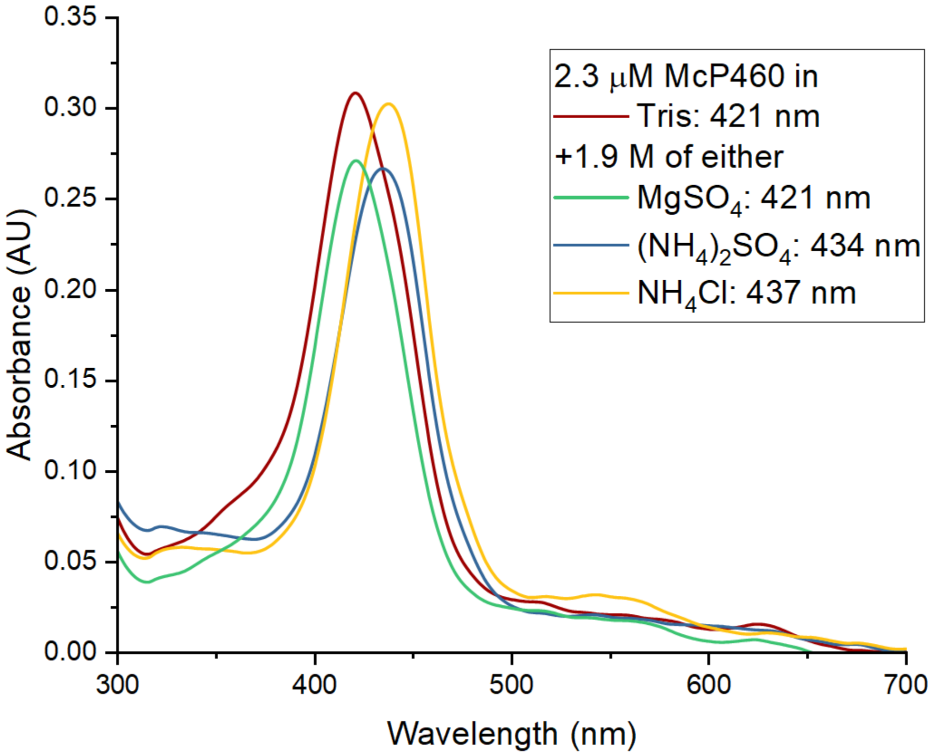

the

Fig. S10. Solution UV-vis spectroscopy of McP460 at pH 8.0, with peak wavelengths in the inset box.

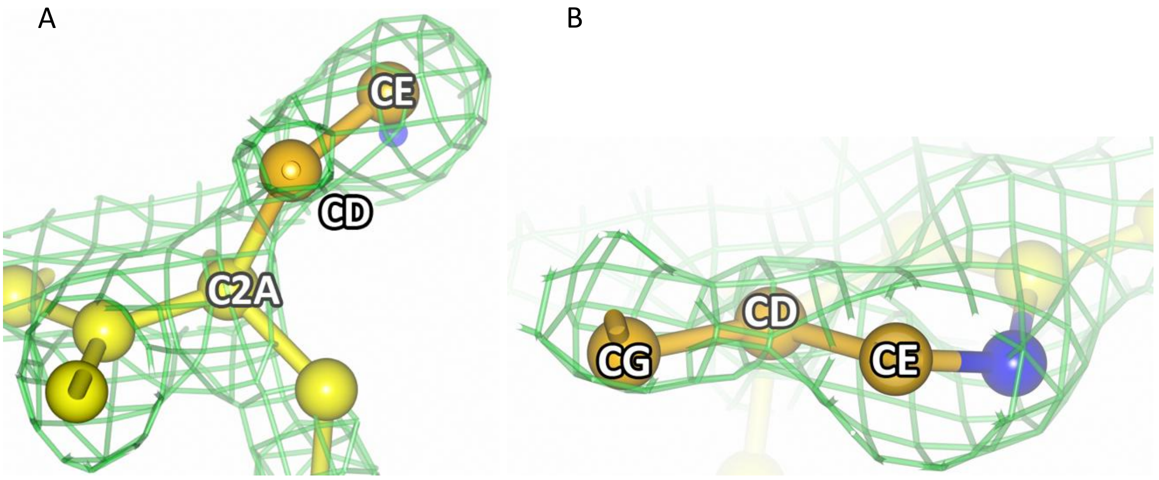

Fig. S11. Composite omit electron density map (light green) around the **RT fresh** crystal structure. It suggests a planar arrangement of atoms around the crosslinking CD of the lysine. Atoms are represented as ball and stick, with the haem in yellow and the lysine carbons in orange. A) Looking down the CG-CD bond. B) Looking down the CD-C2A bond. Contoured at 7.5 electrons/Å^3 (just high enough to clearly see the hole in the newly formed ring, as for Fig. 1D).

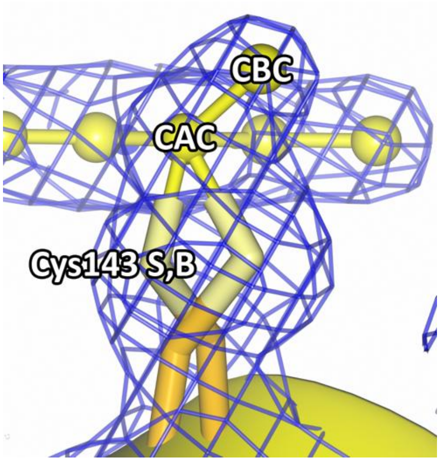

Fig. S12. RT fresh alternate conformation of Cys143, here in chain B, with its sulphur atom labelled. The polypeptide is represented as a yellow ribbon, the side chains carbons as orange sticks, and the haem as yellow ball-and-stick with two carbon atoms labelled. The blue mesh represents the *2-Fo–Fc* electron density map contoured at 0.30 electrons/Å^3.

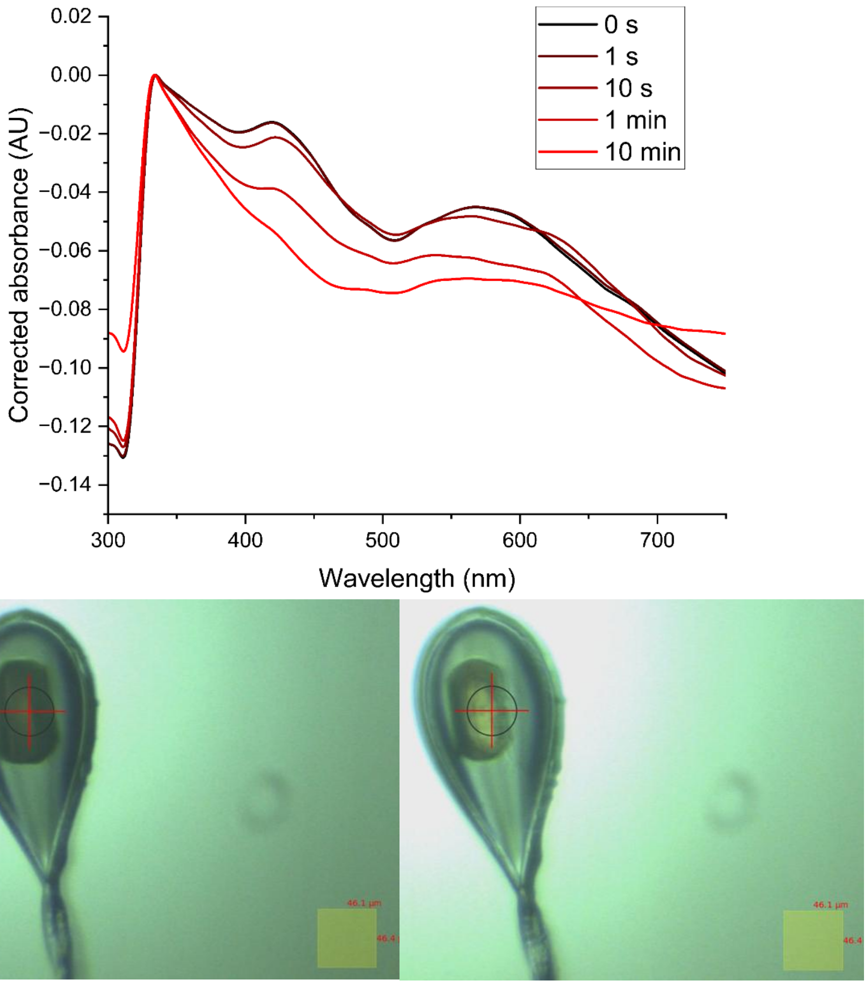

Fig. S13. Room-temperature single-crystal UV-vis spectroscopy (top panel) during exposure to a 20 keV X-ray beam of dimensions 50×50 µm delivering 3.1 MGy of radiation dose to the exposed area per 1 min; photographic images of cytochrome P460 crystals before and after 10 min of X-ray exposure (bottom panel).

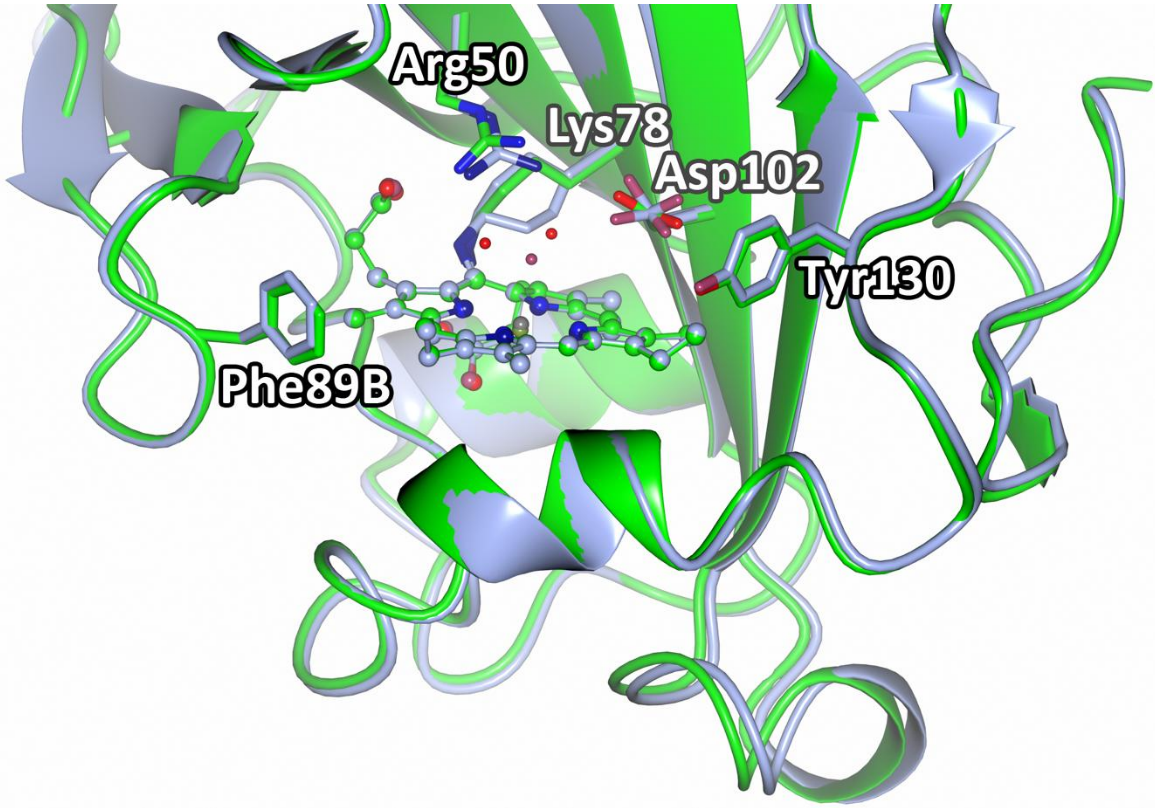

Fig. S14. Superposition of Ferrous cryo structure (green with oxygen atoms in red) and the previously published (PDB:6HIU) ferric cryo structure (ice blue with oxygen atoms in purple), showing chain A and a loop of chain B harbouring Phe89. The polypeptide of the ferrous structure (green) is visibly contracted compared to the ferric structure. The iron atom in the ferrous state (olive) has sunk 0.3 Å towards the proximal side of the haem compared to the ferrous state.

Table S3A. Absolute and relative energies of QM/MM optimised Ferric-SC/DC/DCu systems in all three spin states.

	Chain A (Hartree)	Relative energies wrt lowest energy spin state of same chain (kcal/mol)	Chain B (Hartree)	Relative energies wrt lowest energy spin state of same chain (kcal/mol)	Relative energies wrt lowest energy spin state of chain A (kcal/mol)
**Ferric-SC**					
**M=2**	-3840.45	7.14	-3840.46	5.27	2.77
**M=4**	-3840.46	0.65	**-3840.47**	**0.00**	**-2.50**
**M=6**	**-3840.46**	**0.00**	-3840.46	4.94	2.43
**Ferric-DC**					
**M=2**	-3878.43	0.88	**-3878.44**	**0.00**	**-1.10**
**M=4**	-3878.42	8.85	-3878.43	4.14	3.04
**M=6**	**-3878.44**	**0.00**	-3878.43	5.31	4.21
**Ferric-DCu**					
**M=2**	**-3877.11**	**0.00**	-3877.11	2.25	0.12
**M=4**	-3877.11	0.99	**-3877.12**	**0.00**	**-2.13**
**M=6**	-3877.11	2.70	-3877.11	2.12	-0.02

Table S3B. Absolute and relative energies of QM/MM optimised Ferrous-SC/DC/DCu systems in all three spin states.

	Chain A (Hartree)	Relative energies wrt lowest energy spin state of same chain (kcal/mol)	Chain B (Hartree)	Relative energies wrt lowest energy spin state of same chain (kcal/mol)
**Ferrous-SC**				
**M=1**	-3839.93	8.32	-3839.98	9.86
**M=3**	-3839.94	2.14	-3839.99	2.20
**M=5**	**-3839.95**	**0.00**	**-3839.99**	**0.00**
**Ferrous-DC**				
**M=1**	-3877.84	15.77	-3877.91	7.19
**M=3**	-3877.85	9.87	-3877.92	1.68
**M=5**	**-3877.87**	**0.00**	**-3877.92**	**0.00**
**Ferrous-DCu**				
**M=1**	-3876.49	4.64	-3876.55	9.01
**M=3**	-3876.49	2.28	-3876.56	2.53
**M=5**	**-3876.50**	**0.00**	**-3876.56**	**0.00**

Table S3C. Absolute and relative energies of QM/MM optimised *SimFerrous-SC/DC/DCu* systems in all three spin states.

	**Chain A (Hartree)**	**Relative energies wrt lowest energy spin state of same chain (kcal/mol)**	**Chain B (Hartree)**	**Relative energies wrt lowest energy spin state of same chain (kcal/mol)**
SimFerrous-SC				
**M=1**	-3840.34	5.44	-3840.34	1.99
**M=3**	-3840.34	1.51	**-3840.35**	**0.00**
**M=5**	**-3840.35**	**0.00**	-3840.35	0.20
SimFerrous-DC				
**M=1**	-3878.32	1.56	**-3878.31**	**0.00**
**M=3**	-3878.32	1.65	-3878.31	2.00
**M=5**	**-3878.32**	**0.00**	-3878.30	4.66
SimFerrous-DCu				
**M=1**	-3876.99	2.70	**-3876.99**	**0.00**
**M=3**	-3876.99	3.98	-3876.97	9.27
**M=5**	**-3876.99**	**0.00**	-3876.97	10.92

In tables S3A-C the two chains reported are treated as individual systems.

Table S4. Haem C site parameters for McP460 structures, including: the distance between proximal His143 N and Fe (Fe-HisN), the distance between the coordinated water to Fe (Fe-water), Fe to porphyrin N of all pyrrole rings (Fe-PyrN), the distance between Lys78 and CHA of haem (LysN-CHA), the distance between CD of Lys78 and C2A of haem in DC systems (LysCD-C2A), the distance between haem C3A and CMA distance (C3A-CMA) that undergoes exocyclic modification in DC systems and Fe out of plane motion (Fe-OOP).

A) Ferric-SC/DC/DCu in all spin states and both chains

	**Fe-His N (Å)**		**Fe-water N (Å)**		**Fe PyrN (Å): NA, NB, NC and ND**		**LysN-CHA (Å)**		**LysCD-C2A (Å)**		**C3A-CMA (Å)**		**Fe-OOP (Å)***	
**Chain**	**A**	**B**	**A**	**B**	**A**	**B**	**A**	**B**	**A**	**B**	**A**	**B**	**A**	**B**
**Ferric-SC**														
**M = 2**	1.96	2.00	2.16	2.30	1.99, 2.03, 2.05, 2.00	2.01, 2.01, 2.04, 2.04	1.40	1.41	-	-	1.47	1.47	0.12	0.08
**M = 4**	2.15	2.18	2.54	2.36	1.99, 2.03, 2.04, 2.01	2.01, 2.01, 2.02, 2.02	1.40	1.41	-	-	1.47	1.47	0.14	0.05
**M = 6**	2.09	2.13	2.58	2.32	2.08, 2.07, 2.09, 2.08	2.07, 2.06, 2.08, 2.08	1.35	1.41	-	-	1.47	1.47	0.24	0.10
**Ferric-DC**														
**M = 2**	1.92	1.95	2.35	2.29	2.09, 2.00, 2.01, 2.01	2.09, 2.01, 2.02, 2.02	1.36	1.36	1.57	1.57	1.35	1.35	0.12	0.09
**M = 4**	2.11	2.17	2.75	2.38	2.07, 2.01, 2.01, 2.01	2.11, 2.00, 2.00, 2.01	1.36	1.36	1.57	1.57	1.35	1.35	0.14	0.06
**M = 6**	2.11	2.17	2.68	2.37	2.22, 2.07, 2.06, 2.09	2.20, 2.06, 2.05, 2.09	1.34	1.35	1.57	1.56	1.35	1.35	0.20	0.09
**Ferric-DCu**														
**M = 2**	1.93	1.96	2.33	2.28	2.10, 2.00, 2.01, 2.02	2.11, 2.01, 2.03, 2.03	1.38	1.38	1.54	1.54	1.35	1.35	0.12	0.09
**M = 4**	2.11	2.16	2.51	2.30	2.12, 2.00, 2.00, 2.01	2.12, 2.00, 2.01, 2.01	1.38	1.38	1.54	1.54	1.35	1.35	0.13	0.07
**M = 6**	2.11	2.14	2.49	2.27	2.24, 2.06, 2.04, 2.09	2.22, 2.06, 2.03, 2.09	1.37	1.37	1.53	1.53	1.35	1.35	0.19	0.11

B) Ferrous-SC/DC/DCu in all spin states and both chains

	Fe-His N (Å)		Fe-water N (Å)		Fe PyrN (Å): NA, NB, NC and ND		LysN-CHA (Å)		LysCD-C2A (Å)		C3A-CMA (Å)		Fe-OOP (Å)*	
**Chain**	**A**	**B**	**A**	**B**	**A**	**B**	**A**	**B**	**A**	**B**	**A**	**B**	**A**	**B**
**Ferrous-SC**														
**M = 1**	1.97	1.96	3.11	3.51	2.04, 2.02, 2.03, 2.02	2.02, 2.02, 2.02, 2.05	1.36	1.36	-	-	1.47	1.47	0.15	0.17
**M = 3**	2.24	2.20	3.22	3.53	2.04, 2.01, 2.04, 2.03	2.03, 2.03, 2.03, 2.04	1.35	1.36	-	-	1.47	1.47	0.13	0.16
**M = 5**	2.11	2.08	3.38	3.68	2.14, 2.11, 2.12, 2.10	2.12, 2.12, 2.10, 2.14	1.35	1.35	-	-	1.47	1.47	0.40	0.43
**Ferrous-DC**														
**M = 1**	1.94	1.94	3.26	3.37	2.02, 2.03, 2.03, 2.03	2.02, 2.03, 2.02, 2.04	1.36	1.36	1.57	1.57	1.35	1.35	0.16	0.16
**M = 3**	2.17	2.15	3.31	3.48	2.02, 2.04, 2.03, 2.03	2.03, 2.04, 2.02, 2.04	1.36	1.36	1.57	1.57	1.35	1.35	0.15	0.17
**M = 5**	2.11	2.10	3.26	3.56	2.15, 2.08, 2.11, 2.10	2.13, 2.09, 2.11, 2.11	1.37	1.36	1.57	1.57	1.35	1.35	0.30	0.31
**Ferrous-DCu**														
**M = 1**	2.01	1.95	2.28	3.37	2.04, 2.02, 2.05, 2.05	2.04, 2.03, 2.02, 2.04	1.39	1.38	1.54	1.54	1.35	1.35	0.06	0.16
**M = 3**	2.19	2.16	3.14	3.46	2.04, 2.03, 2.04,2.04	2.05, 2.04, 2.03, 2.04	1.38	1.38	1.54	1.54	1.35	1.35	0.13	1.17
**M = 5**	2.13	2.10	3.21	3.56	2.14, 2.07, 2.12, 2.09	2.14, 2.09, 2.12, 2.10	1.38	1.38	1.54	1.54	1.35	1.35	0.26	0.31

C) *SimFerrous-SC/DC/DCu* in all spin states and both chains

	Fe-His N (Å)		Fe-water N (Å)		Fe PyrN (Å): NA, NB, NC and ND		LysN-CHA (Å)		LysCD-C2A (Å)		C3A-CMA (Å)		Fe-OOP (Å)*	
**Chain**	**A**	**B**	**A**	**B**	**A**	**B**	**A**	**B**	**A**	**B**	**A**	**B**	**A**	**B**
SimFerrous-SC														
**M = 1**	2.03	2.00	2.27	2.33	2.00, 2.05, 2.07, 2.03	2.04, 2.04, 2.04, 2.03	1.41	1.41	-	-	1.47	1.48	0.09	0.09
**M = 3**	2.23	2.24	3.28	3.05	2.01, 2.05, 2.05, 2.03	2.04, 2.04, 2.03, 2.04	1.41	1.41	-	-	1.47	1.47	0.14	0.13
**M = 5**	2.16	2.08	3.30	2.98	2.08, 2.11, 2.14, 2.10	2.13, 2.11, 2.12, 2.17	1.41	1.41	-	-	1.47	1.47	0.29	0.44
SimFerrous-DC														
**M = 1**	1.99	1.97	2.28	2.30	2.03, 2.04, 2.06, 2.05	2.04, 2.07, 2.05, 2.03	1.37	1.37	1.57	1.57	1.35	1.35	0.09	0.1
**M = 3**	2.16	2.16	3.32	3.25	2.04, 2.05, 2.02, 2.04	2.05, 2.04, 2.02, 2.04	1.37	1.37	1.57	1.57	1.35	1.35	0.16	0.16
**M = 5**	2.15	2.05	2.61	2.94	2.11, 2.09, 2.14, 2.10	2.19, 2.09, 2.10, 2.16	1.37	1.37	1.56	1.57	1.35	1.35	0.21	0.46
SimFerrous-DCu														
**M = 1**	2.00	1.99	2.27	2.26	2.05, 2.04, 2 .05, 2.06	2.05, 2.04, 2.06, 2.06	1.39	1.39	1.54	1.54	1.35	1.35	0.09	0.09
**M = 3**	2.17	2.18	3.33	2.96	2.05, 2.04, 2.03, 2.04	2.06, 2.03, 2.02, 2.05	1.39	1.39	1.54	1.54	1.35	1.35	0.16	0.13
**M = 5**	2.16	2.07	2.50	2.87	2.12, 2.09, 2.13, 2.10	2.18, 2.06, 2.10, 2.14	1.39	1.39	1.53	1.54	1.35	1.35	0.20	0.36

Table S5. DFT cluster calculations of Ferric-DC system without any environment

	Chain A (Hartree)	Relative energies wrt lowest energy spin state of chain A (kcal/mol)	Chain B (Hartree)	Relative energies wrt lowest energy spin state of chain A (kcal/mol)
Ferric-DC without environment				
M=2	-3875.65	2.85	-3875.65	2.66
M=4	**-3875.65**	**0.00**	**-3875.65**	**0.00**
M=6	-3875.64	5.65	-3875.64	5.68

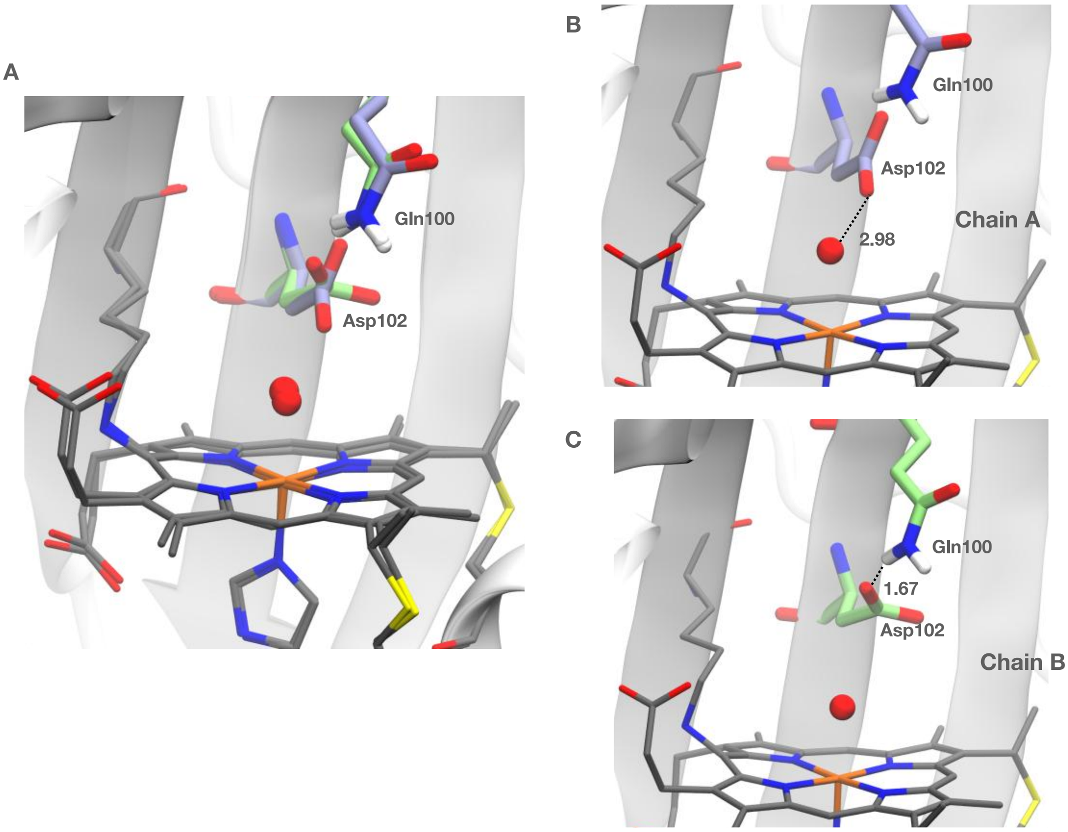

Fig. S15. Chain A and B of QM/MM optimized **ferric SFX** with SC in M=6 state showing A) the two alternating conformations of active site residue Asp102 in chain A (light blue) and chain B (light green). B) Interaction of Asp102 in chain A with the water coordinating to Fe and C) Interaction of Asp102 with another active site residue Gln100. The H-atoms for all residues except the amine group of Gln102 was removed for clarity. Distances are in Å.

S.3 MD analysis of Ferric-SC/DC systems

The MD trajectories of Ferric-SC and DC systems were analysed to identify water network patterns in the active site of the protein. The last 150 ns of the 175 ns of the MD was considered for the production run and analysis were performed on it. The Ferric **SFX** structure indicated water coordinating to Fe of haem so the MD trajectory was analysed for the presence of a coordinated water by calculating the distance between Fe atom and any water molecule (oxygen atom of the water molecule) within 3.5 Å of the Fe atom. The evolution of the distance of a coordinated water to Fe with time is given in the top panel of Fig. S16 A(I) for SC and A(II) for DC. Each frame corresponds to 0.1 ns. The average distance of this water from chain A and chain B for SC are 2.73 ± 0.20 Å and 3.24 ± 0.16 Å, respectively. These distances are very similar for DC: 2.8 ± 0.21 Å and 3.25 ± 0.15 Å for chain A and B, respectively. Next, the residence time of water molecule near the Fe-atom was calculated as a fraction of time the water molecule was present within 3.5 Å of Fe. To do so, the frequency a water was present was binned at intervals of discrete distance with a bin width of 0.2 Å, normalised to the total time and represented as histograms in the lower panel of Fig. S16 A(I) and A(II) representing SC and DC, respectively. In a similar manner, the frequency of H-bond interactions of the water molecule with the pyrrole N atoms of porphyrin ring was calculated as a fraction of the total time, binned with respect to the pyrrole N and plotted as histograms. The same was done for calculating H-bond interactions with the active site residues; the frequency of the presence of H-bonds was calculated as a fraction of the total time, binned with respect to the active site residue and plotted as histograms.

Furthermore, to verify that the difference in the residence time of the coordinated water in the two chains was a result of the orientation of Asp102, the MD simulations for both Ferric-SC and

Ferric-DC system were repeated restraining the backbone of the protein with a force constant of 25 kcal/Å^2 and allowing the side chains to relax. The same analyses were performed, and the results reveal restoration of similar distribution of water around the active site in both chains (SI Fig. 17) for both SC and DC systems. In case of the SC, the Fe-water distance is 2.85 ± 0.24 Å and 2.80 ± 0.25 Å for chain A and B, respectively and that for double cross link it is 2.63 ± 0.16 Å and 2.62 ± 0.16 Å for chain A and B, respectively. The water has negligible interaction with pyrrole N atoms. The interaction with Asp102 is similar in the two chains but quite different between SC and DC systems. In the SC system, water predominantly interacts with Asp102, whereas in double crosslink system this water interacts with both Asp102 and Arg50.

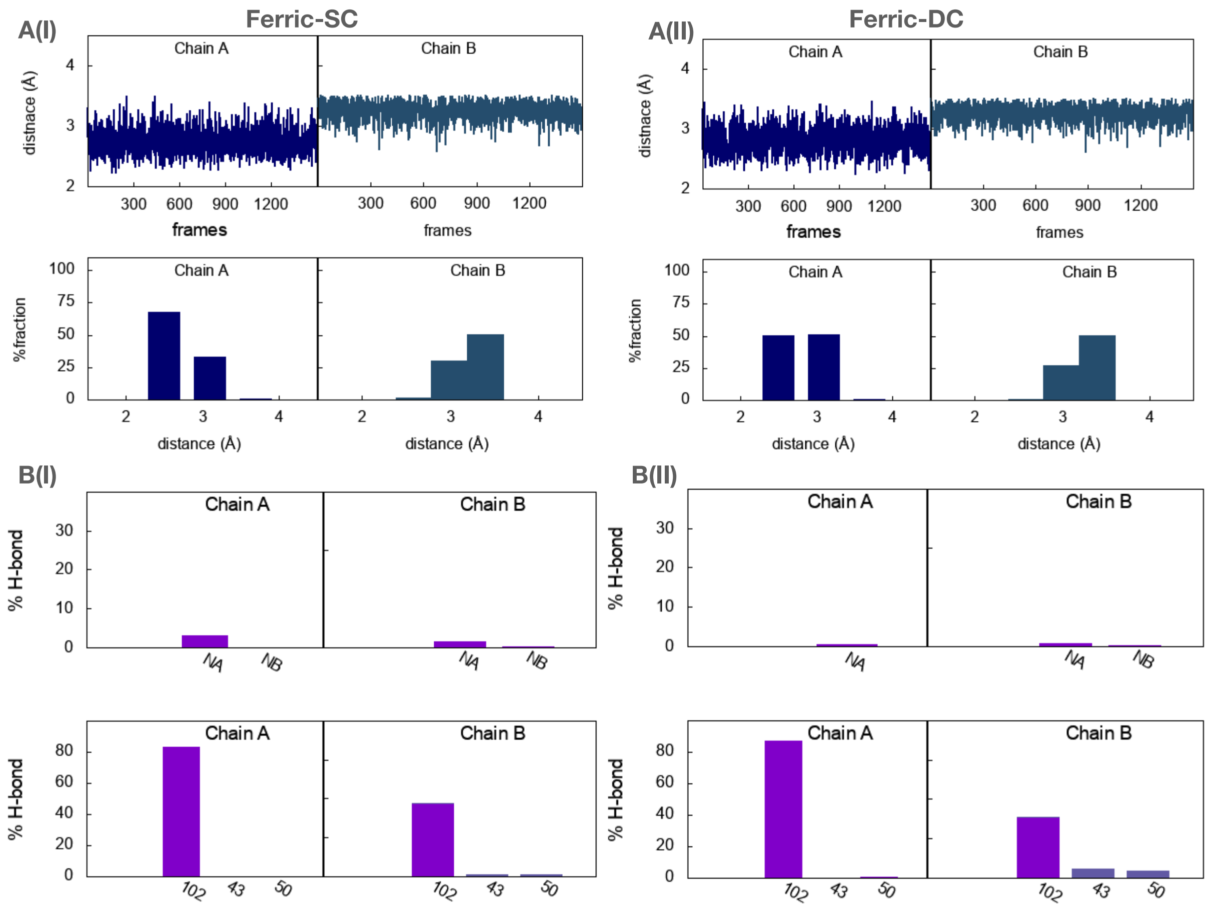

Fig. S16. (A) Evolution of the distance of water from Fe along the MD trajectory (top panel) and residence time of that water at discrete distance intervals (lower panel) is given for (I) for Ferric-SC and (II) for Ferric-DC. (B) Interaction of the above water close to Fe with N-atoms of pyrrole (top panel) and with active site residues Asp102, Arg43 and Arg50 (bottom panel) for (I) Ferric-SC and (II) Ferric-DC. Different water molecules are shown in different colours in the histogram.

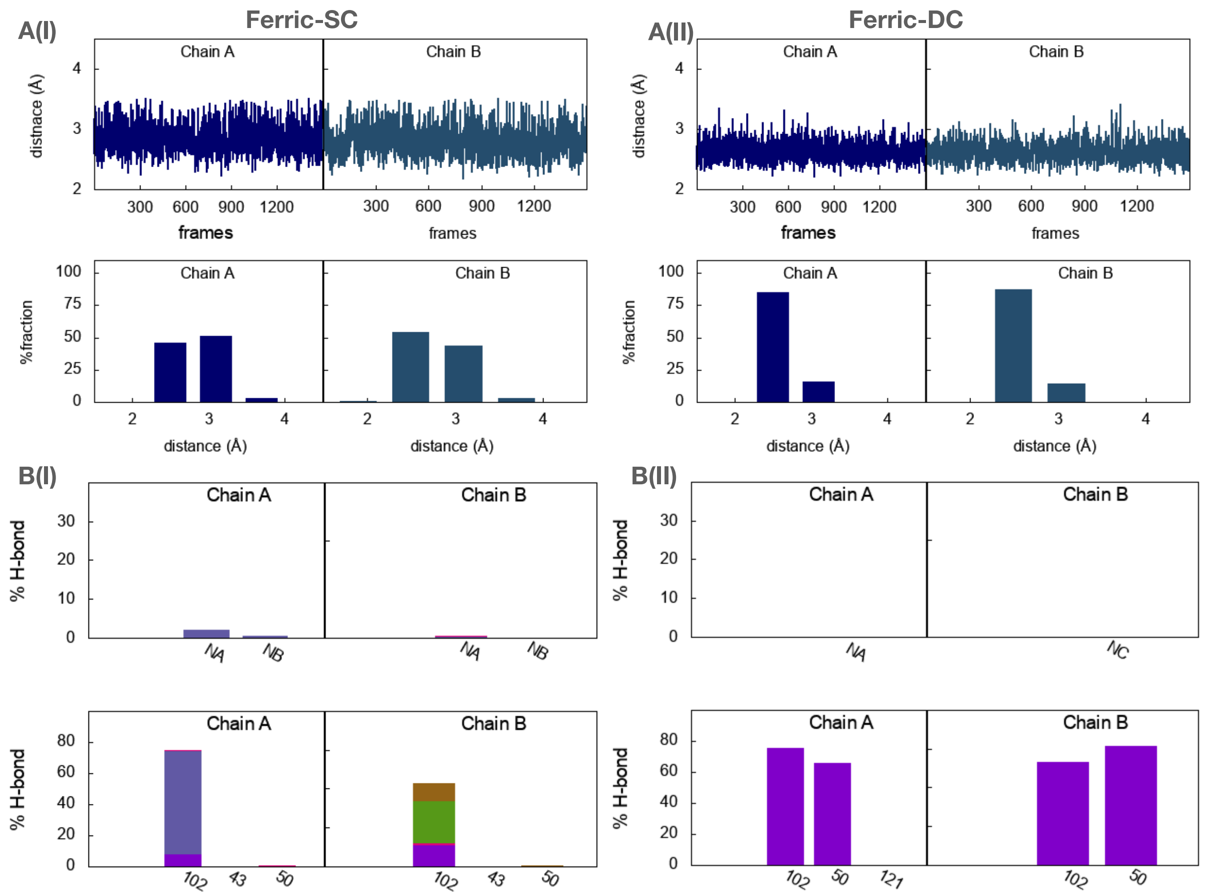

Fig. S17. (A) Evolution of the distance of water from Fe along the MD trajectory (top panel) and residence time of that water at discrete distance intervals (lower panel) for: (I) Ferric-SC and (II) Ferric-DC. (B) Interaction of the same water close to Fe with N-atoms of pyrrole (top panel) and with active site residues Asp102, Arg43 and Arg50 (bottom panel) for (I) Ferric-SC and (II) Ferric-DC. Different water molecules are shown in different colours in the histogram. Here, the backbone of the protein was restrained with a 25kcal/mol/$Å^2$ force constant and the side chains were allowed to move.

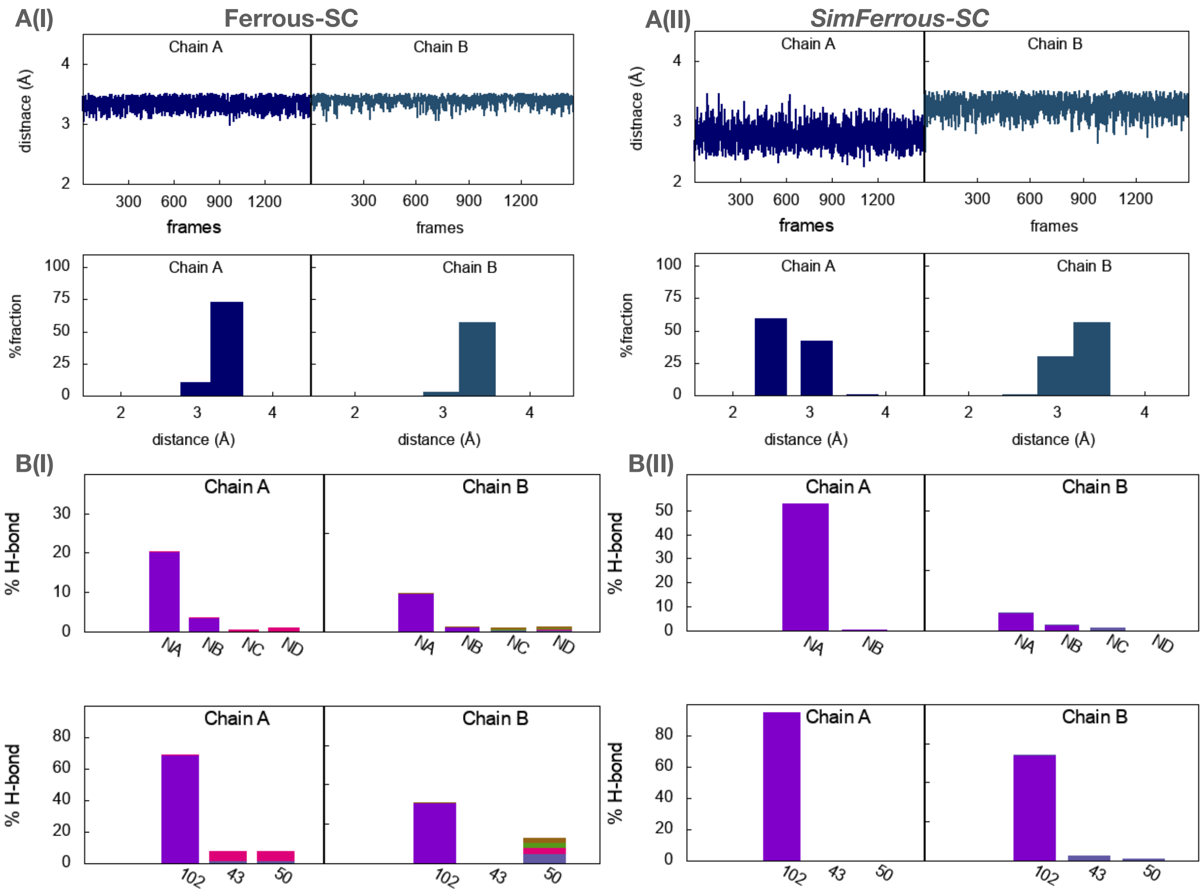

Fig. S18. (A) Evolution of the distance of water from Fe along the MD trajectory (top panel) and residence time of that water at discrete distance intervals (lower panel) is given for (I) Ferrous-SC and (II) *SimFerrous-SC*. (B) Interaction of the same water close to Fe with N-atoms of pyrrole (top panel) and with active site residues Asp102, Arg43 and Arg50 (bottom panel) for (I) Ferrous-SC and (II) *SimFerrous-SC*. Different water molecules are shown in different colours in the histogram.

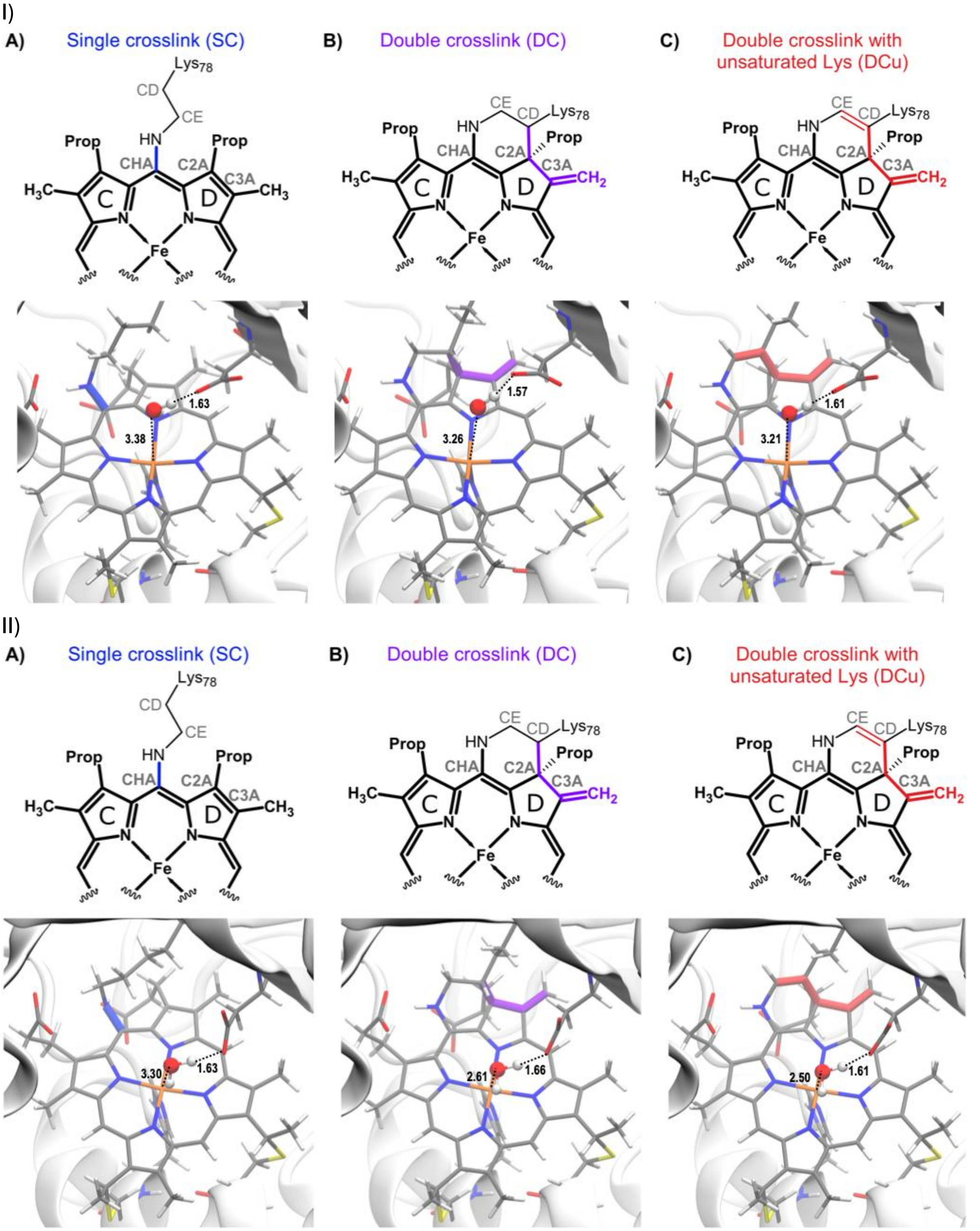

Fig. 19. I) QM/MM optimised geometries of A) Ferrous-SC, B) Ferrous-DC and C) Ferrous-DCu. (II) QM/MM optimised geometries of A) *SimFerrous-SC*, B) *SimFerrous-DC* and C) *SimFerrous-DCu*. The haem C unit is shown with sticks and the different crosslinks are shown in the same colour as in Scheme 1 (main manuscript). The 6th site coordinated water is shown in ball and stick. The active site residue Asp102 (which interacts with the coordinated water) is shown in sticks. Distances are given in Å.

Table S6. Excitation energies calculated using TD-DFT for all spin states for the three proposed crosslink models (wavelengths in nm). Oscillator strengths for each excitation are given in brackets.

**Model**	**Ferrous nm (f_{osc}) (M=1)**	**SimFerrous nm (f_{osc}) (M=1)**	**Ferrous nm (f_{osc}) (M=3)**	**SimFerrous nm (f_{osc}) (M=3)**	**Ferrous nm (f_{osc}) (M=5)**	**SimFerrous nm (f_{osc}) (M=5)**	**Ferric nm (f_{osc}) (M=2)**	**Ferric nm (f_{osc}) (M=4)**	**Ferric nm (f_{osc}) (M=6)**
DC	405.3 (0.46)<br>379.0 (0.45)	406.0 (0.47)<br>387.6 (0.54)	429.3 (0.42)	403.1 (0.52)	419.3 (0.61)<br>389.4 (0.46)<br>388.3 (0.15)<br>383.5 (0.17)	418.0 (0.65)<br>391.2 (0.60)<br>388 (0.19)	410.4 (0.14)<br>396.2 (0.14)<br>364.4 (0.47)	455.6 (0.1)<br>419.0 (0.1)	453.7 (0.17)<br>388.1 (0.33)<br>375.5 (0.23)<br>369.2 (0.39)
DCu	415.3 (0.32)<br>394.2 (0.56)	409.9 (0.48)<br>393.8 (0.65)	436.1 (0.22)<br>400.9 (0.45)	434.6 (0.25)	430.9 (0.23)<br>430.0 (0.3)<br>411.2 (0.12)<br>402.0 (0.57)	425.5 (0.52)<br>399.9 (0.57)	397.3 (0.16)<br>369.4 (0.11)<br>368.1 (0.38)<br>361.1 (0.13)	424.2 (0.16)	567.7 (0.1)<br>408.7 (0.2)<br>379.8 (0.4)
SC	377.6 (0.8)<br>371.4 (0.57)	389.7 (0.24)	390.3 (0.64)	410.2 (0.15)<br>397.5 (0.21)	395.4 (0.1)<br>389.4 (0.59)<br>385.3 (0.4)<br>384.2 (0.13)<br>382.4 (0.24)	401.1 (0.38)<br>387.4 (0.52)<br>360.7 (0.37)	358.7 (0.62)<br>357.6 (0.46)	361.8 (0.25)	386.0 (0.13)<br>374.3 (0.22)<br>371.2(0.12)<br>361.5(0.26)<br>352.3(0.14)<br>351.3 (0.24)

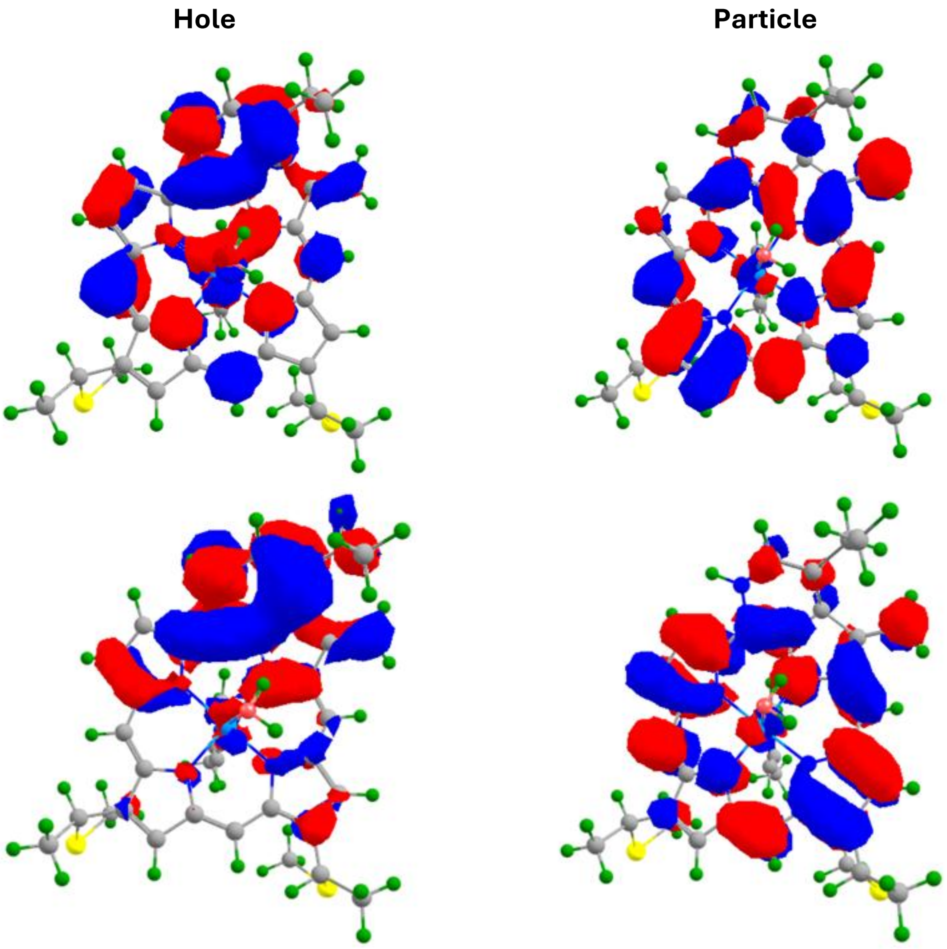

Fig. S20. The hole (left) and particle (right) densities for the NTOs corresponding to states with excitation energies 397 (top) and 368 nm (bottom) that comprise the Soret transition for the Ferric-DCu model. These were computed using TD-DFT with the CAM-B3LYP functional and def2-SVP basis set on all atoms other than Fe, which was treated using def2-TZVP.

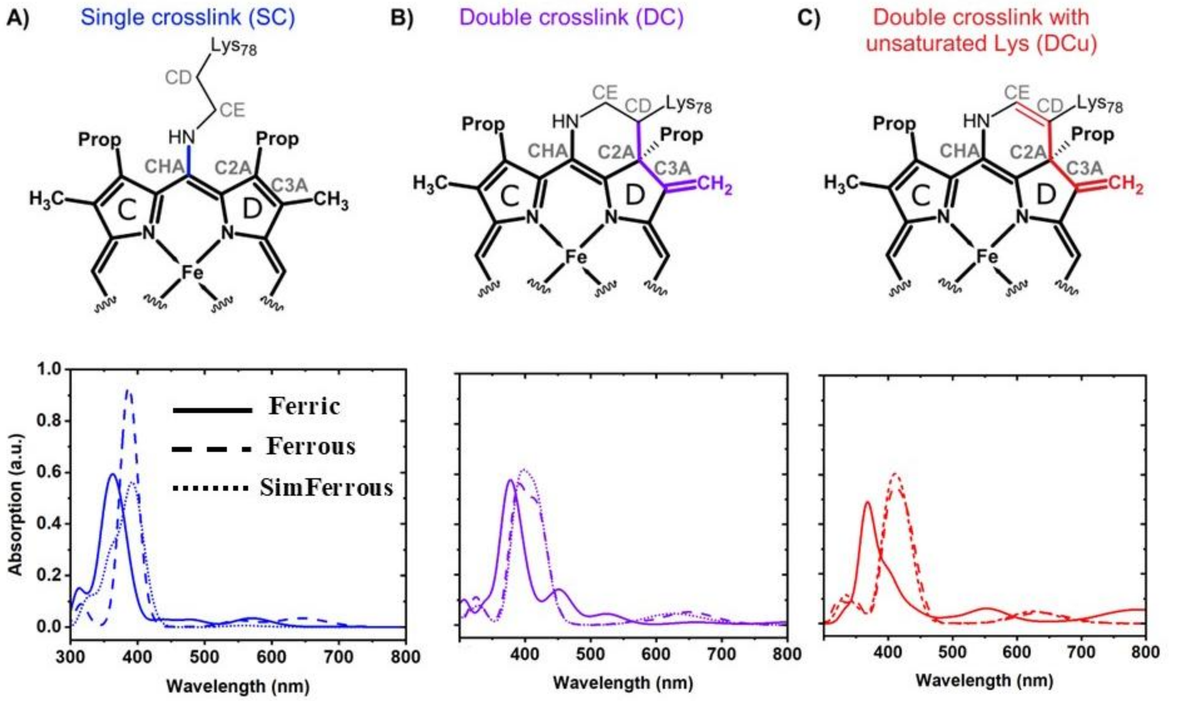

Fig. S21. Computed absorption spectra for the three crosslink models using CAM-B3LYP (TD-DFT) for the lowest energy spin states. Ferrous and *SimFerrous* forms for all models are in a quintet spin state whereas the Ferric forms of SC and DC are in a sextet spin state and DCu is in doublet spin state.

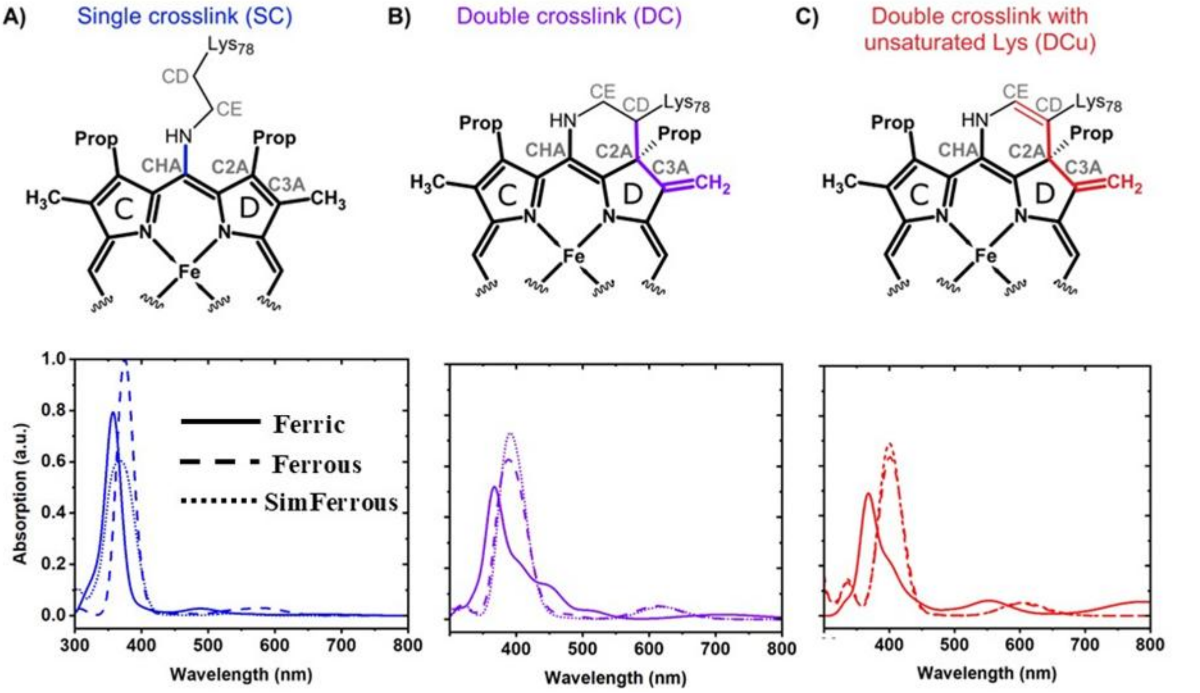

Fig. S22. Computed absorption spectra for the three crosslink models using CAM-B3LYP (TD-DFT) for the lowest spin states. The Ferrous and *SimFerrous* forms for all models are in a singlet spin state whereas the Ferric forms are in a doublet spin state

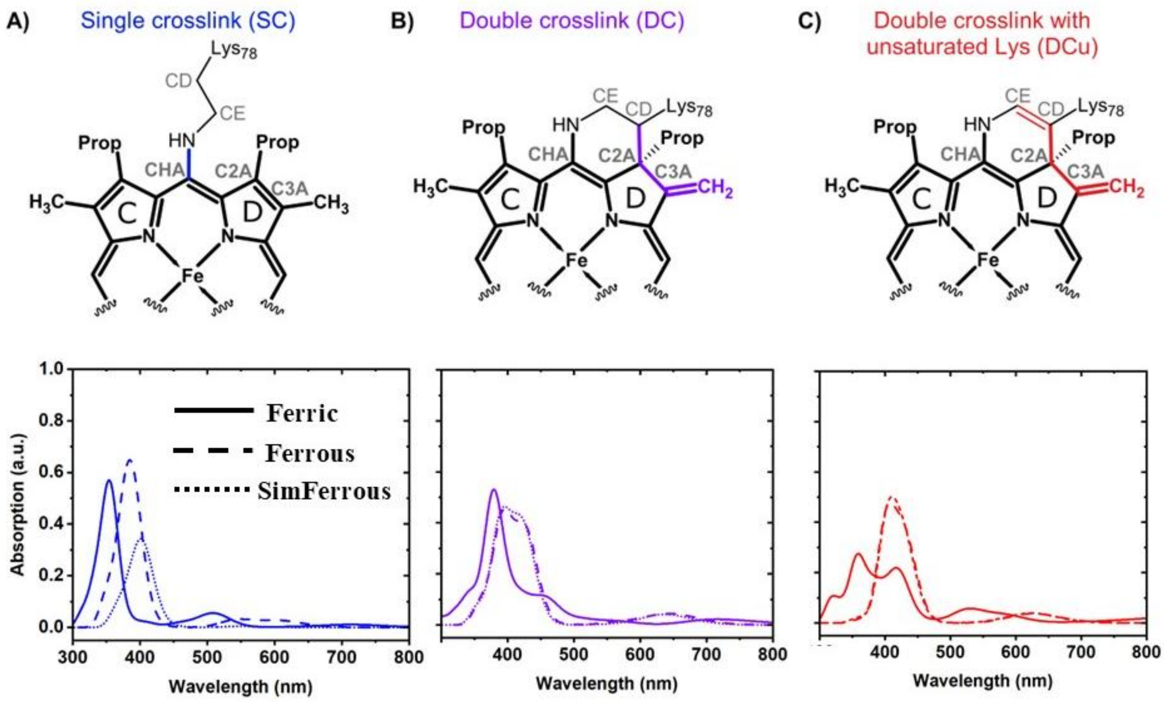

Fig. S23. Computed absorption spectra for the three crosslink models using CAM-B3LYP (TD-DFT). The Ferrous and *SimFerrous* forms for all models are in a triplet spin state whereas the Ferric forms are in a quartet spin state.

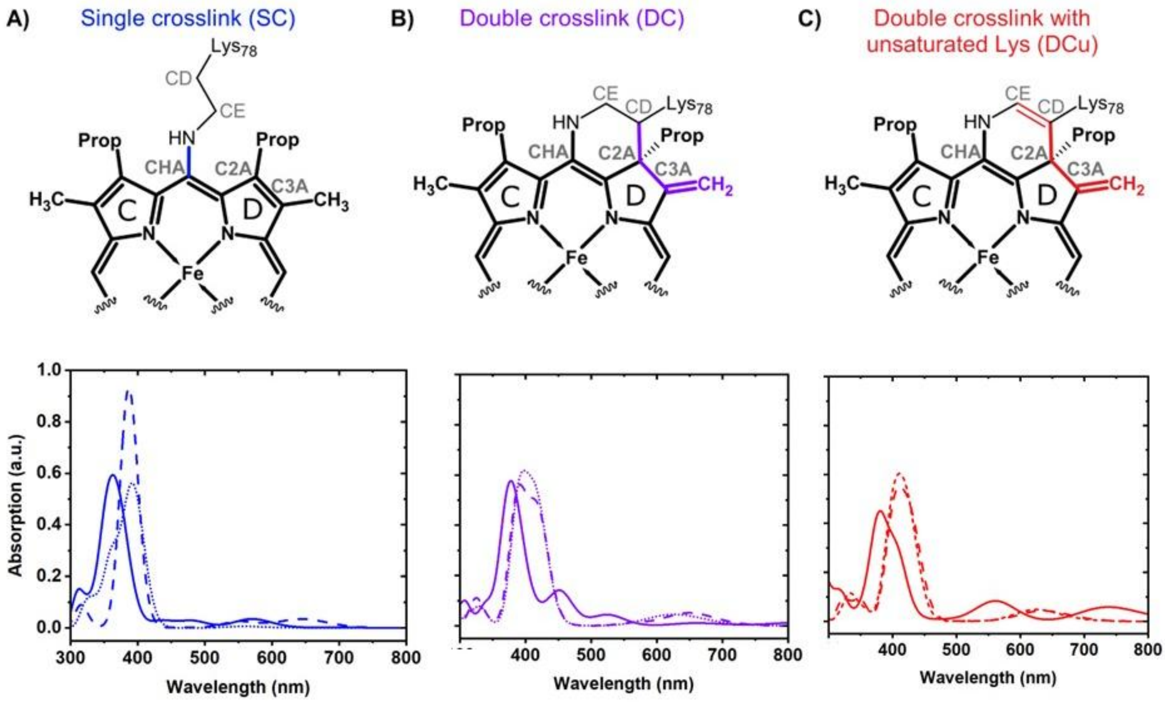

Fig. S24. Computed absorption spectra for the three crosslink models using CAM-B3LYP (TD-DFT). The Ferrous and *SimFerrous* forms for all models are in a quintet spin state whereas the Ferric forms are in a sextet spin state.

Table S7. Excitation energies calculated using sTD-DFT for all spin states for the three proposed crosslink models (wavelengths in nm). Oscillator strengths for each excitation are given in brackets.

Model	Ferrous nm (f_{osc}) (M=1)	SimFerrous nm (f_{osc}) (M=1)	Ferrous nm (f_{osc}) (M=3)	SimFerrous nm (f_{osc}) (M=3)	Ferrous nm (f_{osc}) (M=5)	SimFerrous nm (f_{osc}) (M=5)	Ferric nm (f_{osc}) (M=2)	Ferric nm (f_{osc}) (M=4)	Ferric nm (f_{osc}) (M=6)
DC	474.1<br>(0.68)<br>425.5<br>(0.79)	471.5 (0.56)<br>453.8 (0.39)	466.2<br>(0.84)<br>425.0<br>(0.82)	462.8 (0.89)<br>423.6 (0.78)	459.4<br>(0.94)<br>417.4<br>(0.92)	455.6 (0.99)<br>424.1 (0.34)	429.1<br>(0.25)<br>414.2<br>(0.28)	396.8<br>(0.62)	536.7<br>(0.14)<br>412.9<br>(0.64)<br>378.8<br>(0.42)
DCu	480.8<br>(0.37)<br>456.2<br>(0.43)<br>439.1<br>(0.62)	474.4 (0.33)<br>458.6 (0.46)<br>437.2 (0.70)	473.2<br>(0.71)<br>435.2<br>(0.68)	470.9 (0.74)<br>436.5 (0.4)	470.3<br>(0.75)<br>438.0<br>(0.72)<br>365.9<br>(0.19)	466.1 (0.87)<br>436.1 (0.72)	412.3<br>(0.27)	393.7<br>(0.36)	428.5<br>(0.57)<br>405.4<br>(0.33)
SC	418.2<br>(1.17)<br>404.7<br>(0.66)	423.9 (0.60)<br>418.0 (0.80)	419.4<br>(0.90)<br>412.3<br>(0.65)	434.1 (0.54)<br>425.3 (0.67)<br>357.2 (0.48)	418.6<br>(1.18)<br>411.0<br>(0.64)	430.1 (0.7)<br>421.5 (0.82)	486.6<br>(0.12)<br>389.9<br>(0.33)<br>374.7<br>(0.42)	377.3<br>(0.43)<br>375.5<br>(0.5)	427.4<br>(0.11)<br>398.2<br>(0.49)

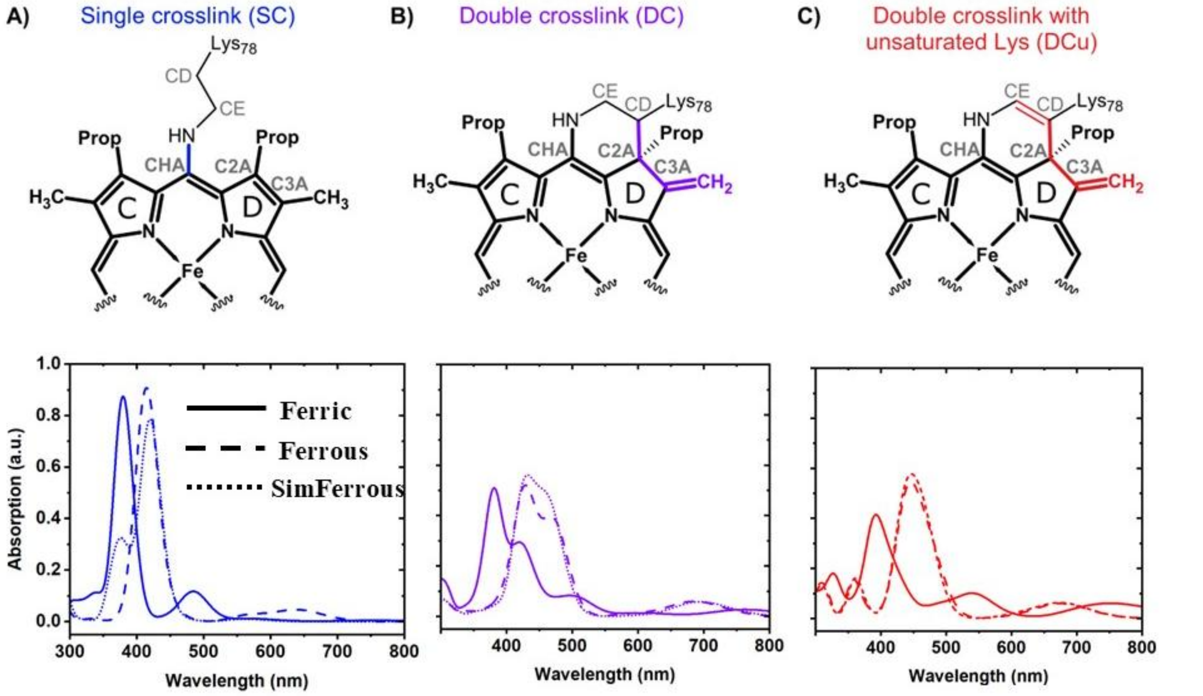

Fig. S25. Computed absorption spectra for the three crosslink models using CAM-B3LYP (sTD-DFT) for the lowest spin states. The Ferrous and *SimFerrous* forms for all models are in a singlet spin state whereas the Ferric forms are in a doublet spin state.

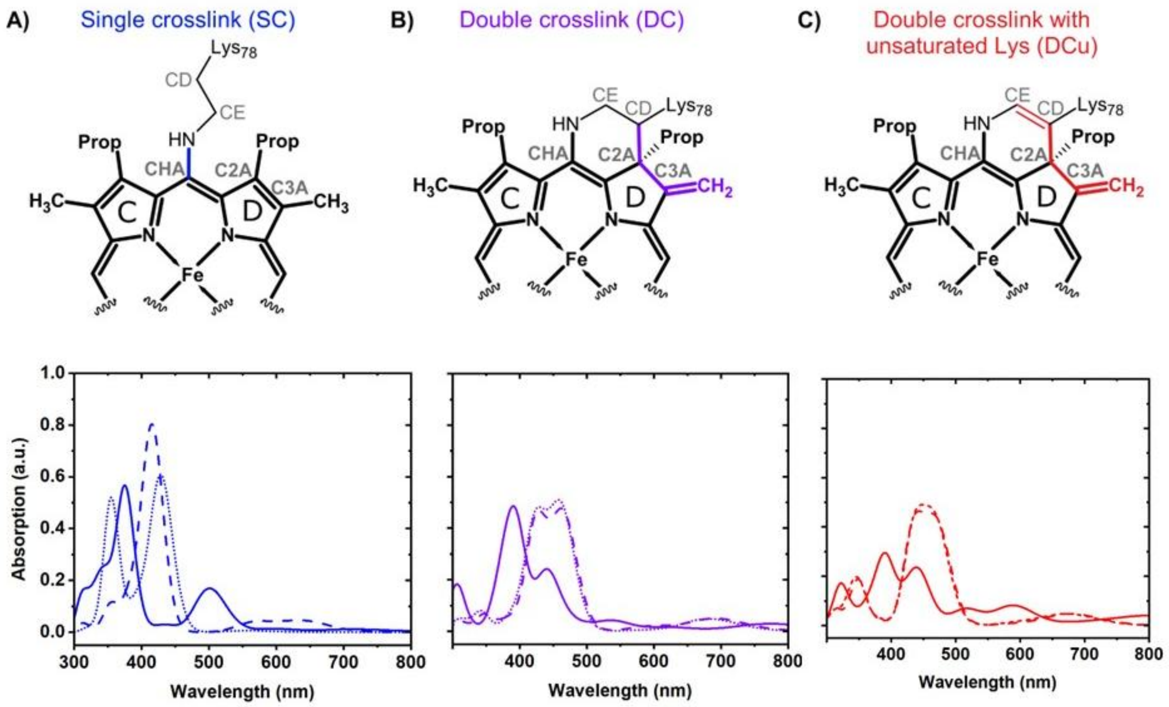

Fig. S26. Computed absorption spectra for the three crosslink models using CAM-B3LYP (sTD-DFT). The Ferrous and *SimFerrous* forms for all models are in a triplet spin state whereas the Ferric forms are in a quartet spin state.

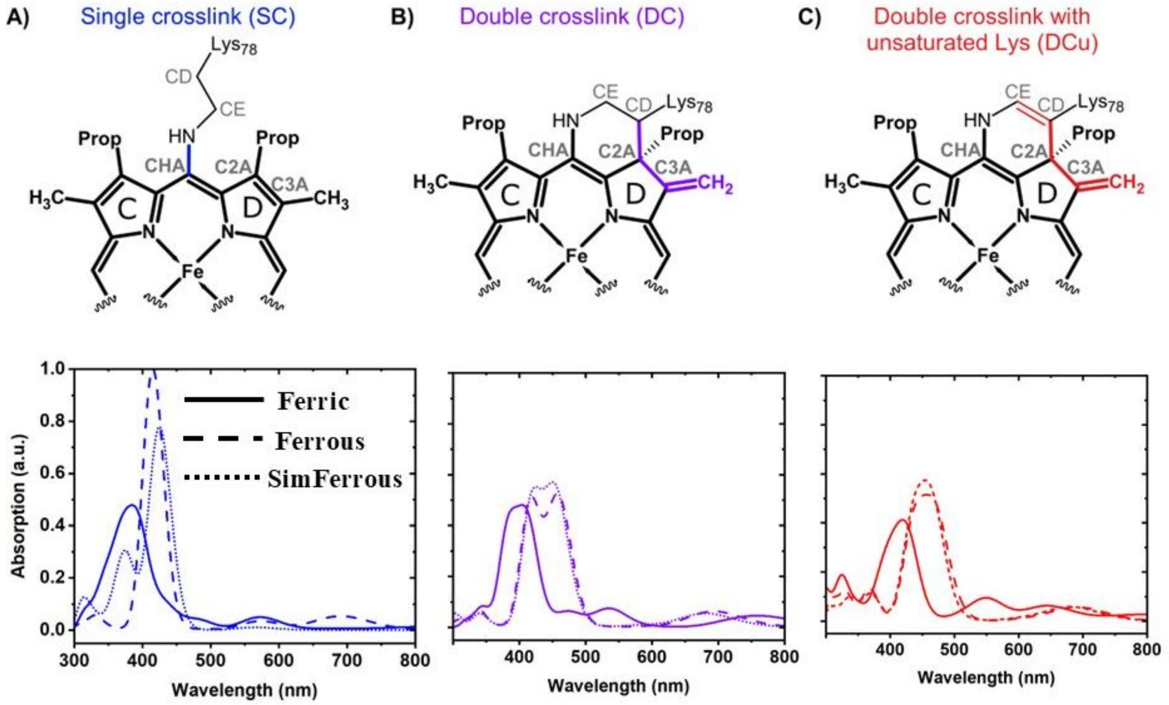

Fig. S27. Computed absorption spectra for the three crosslink models using CAM-B3LYP (sTD-DFT). The Ferrous and *SimFerrous* forms for all models are in a quintet spin state whereas the Ferric forms are in a sextet spin state.

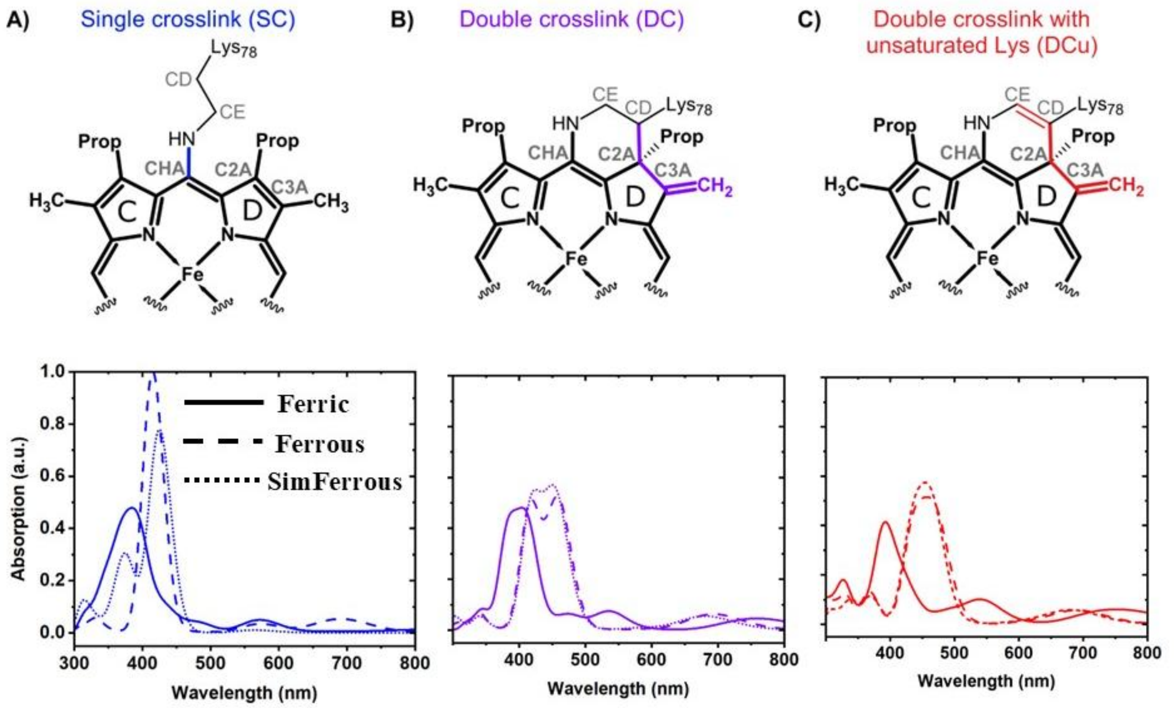

Fig. S28. Computed absorption spectra for the three crosslink models using CAM-B3LYP (sTD-DFT) for the lowest energy spin states. Ferrous and *SimFerrous* forms for all models are in a quintet spin state whereas the Ferric forms of SC and DC are in a sextet spin state and DCu is in doublet spin state.

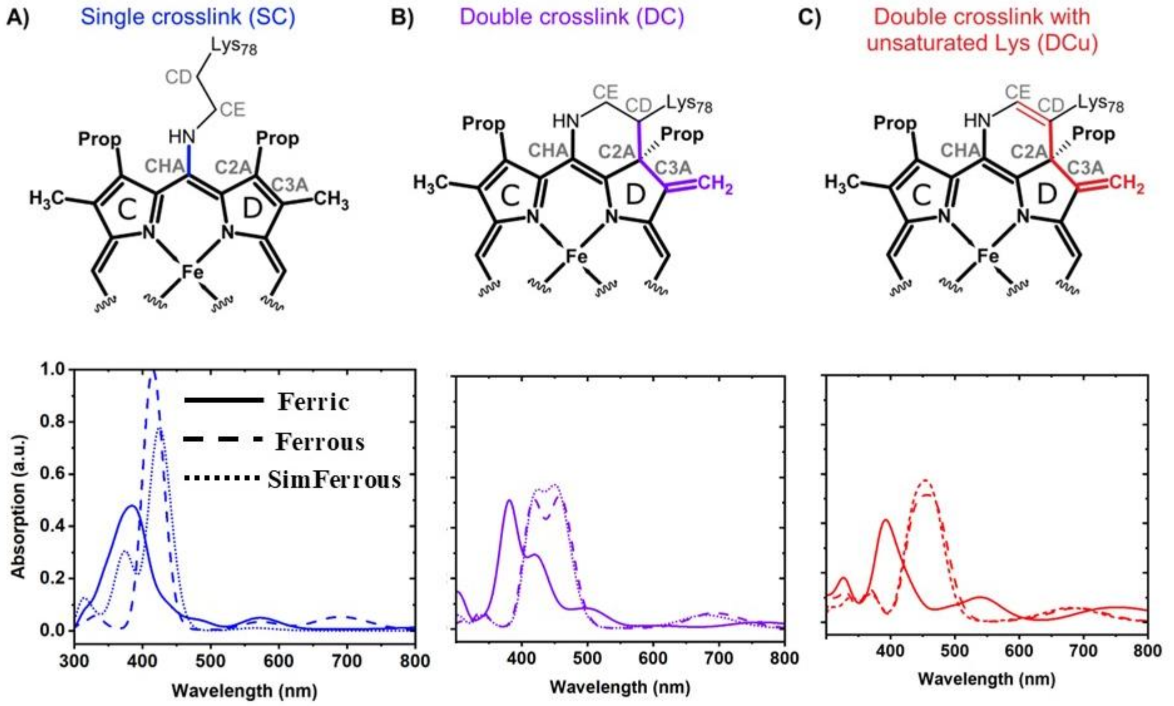

Fig. S29. Computed absorption spectra for the three crosslink models using CAM-B3LYP (sTD-DFT). Ferrous and *SimFerrous* forms for all models are in a quintet spin state whereas the Ferric forms of DC and DCu are in a doublet spin state and SC is in sextet spin state.

Table S8. Excitation energies calculated using ZINDO/S for the lowest spin state for the three proposed crosslink models (wavelengths in nm). Oscillator strengths for each excitation are given in brackets. The states correspond to a ππ* transition within the haem moiety. The absorption spectra computed by removing the point charges loses the direction of the shift. However, the DC models still display electronic transitions close to the experiment Soret peak at 460 nm.

Model	ZINDO/S			ZINDO/S without pointcharges		
	Ferrous<br>nm (f_{osc})	SimFerrous<br>nm (f_{osc})	Ferric<br>Nm (f_{osc})	Ferrous<br>nm (f_{osc})	SimFerrous<br>nm (f_{osc})	Ferric<br>nm (f_{osc})
DC	457.1 (1.33)<br>436.5 (0.43)	452.3(0.86)	426.7 (0.19)	445.9 (1.15)<br>430.5 (0.16)	447.4 (0.25)<br>440.5 (1.05)	458.8 (0.15)<br>421.9 (0.11)
DCu	450.3 (1.08)<br>430.5 (0.92)	449.3 (0.79)	429.9 (0.31)	438.4 (1.19)	443.9 (0.18)<br>437.4 (1.06)	464.4 (0.12)
SC	434.9 (1.20)<br>428.0 (1.41)	418.8 (0.15)	402.5 (1.46)	436.8 (1.11)<br>424.7 (1.34)	401.8 (1.46)<br>399.0 (1.46)	416.6 (0.30)

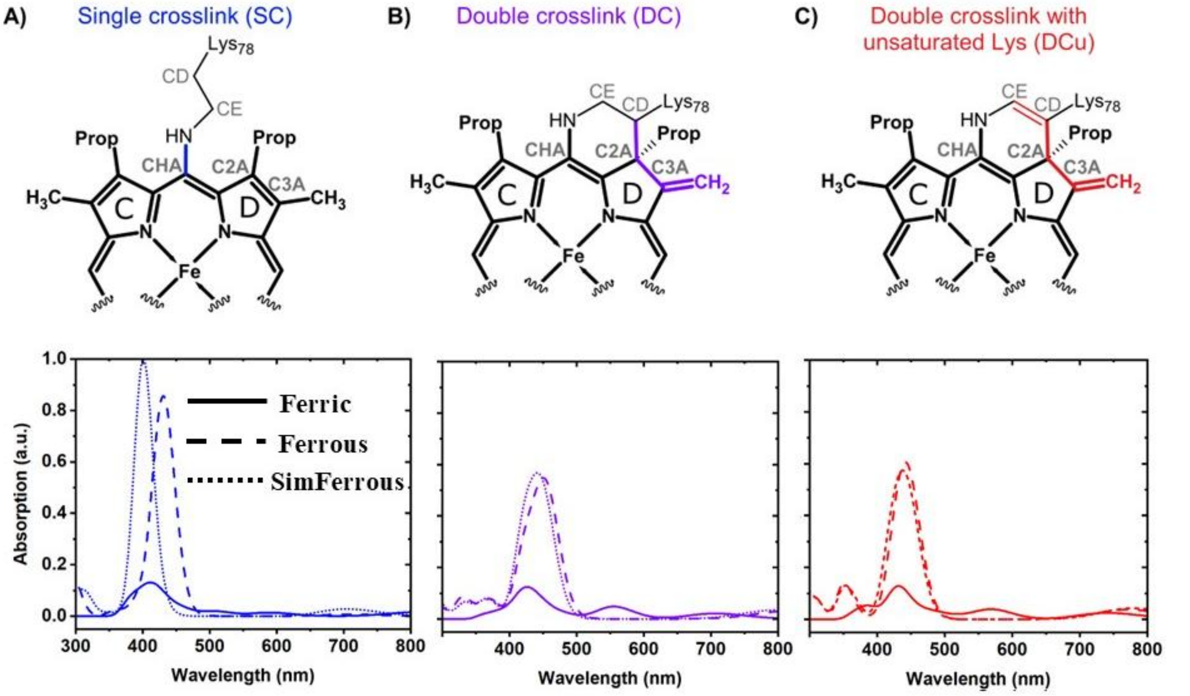

Fig. S30. Computed absorption spectra for the three crosslink models using ZINDO/S for the lowest spin states. The Ferrous and *SimFerrous* forms for all models are in a singlet spin state whereas the Ferric forms are in a doublet spin state. ZINDO/S provides good agreement with experiment for the computed Soret band for Ferrous and Ferric forms.

Appendix: Modified haem restraints (MEH) used for the refinement of SFX and ferrous cryo structures. These require the haem monomer of the PDB file to be renamed from HEC to MEH to apply. The effects of the MEH restraints include allowing more distortion in the plane of the haem than the more restrictive HEC restraints.

```
global_
_lib_name         mon_lib
_lib_version      5.39
_lib_update       06/11/2012
# ------------------------------------------------
#
# ---   LIST OF MONOMERS ---
#
data_comp_list
loop_
_chem_comp.id
_chem_comp.three_letter_code
_chem_comp.name
_chem_comp.group
_chem_comp.number_atoms_all
_chem_comp.number_atoms_nh
_chem_comp.desc_level
MEH     MEH 'HEM SINGLE CAB-CBB CAC-CBC       ' .              77  43.
No       No  '.                           ' .                   3   2.
#
# ---   LIST OF LINKS ---
#
data_link_list
loop_
_chem_link.id
_chem_link.comp_id_1
_chem_link.mod_id_1
_chem_link.group_comp_1
_chem_link.comp_id_2
_chem_link.mod_id_2
_chem_link.group_comp_2
_chem_link.name
CYS-MEHB CYS      CYS-HG   .        MEH      MEH-HABA .
CYS-MEHB
CYS-MEHC CYS      CYS-HG   .        MEH      MEH-HACA .
CYS-MEHC
HIS-MEH  HIS      HIS-HE2  .        MEH      .        .
HIS-MEH
#
# ---   LIST OF MODIFICATIONS ---
#
data_mod_list
loop_
_chem_mod.id
_chem_mod.name
_chem_mod.comp_id
_chem_mod.group_id
CYS-HG   CYSTINE                                      CYS      .
MEH-HABA MEH_DELETED_HABA                             MEH      .
MEH-HACA MEH_DELETED_HACA                             MEH      .
HIS-HE2  HISTIDINE-DELETED-HE2                        HIS      .
#
# --- DESCRIPTION OF MONOMERS ---
#
data_comp_MEH
```

```
#
loop_
_chem_comp_atom.comp_id
_chem_comp_atom.atom_id
_chem_comp_atom.type_symbol
_chem_comp_atom.type_energy
_chem_comp_atom.partial_charge
_chem_comp_atom.x
_chem_comp_atom.y
_chem_comp_atom.z
MEH           O2D    O    OC      -0.500     5.649   -3.481   -2.121
MEH           CGD    C    C        0.000     6.133   -3.500   -0.968
MEH           O1D    O    OC      -0.500     6.686   -2.506   -0.450
MEH           CBD    C    CH2      0.000     6.052   -4.778   -0.159
MEH           HBD    H    H        0.000     6.418   -5.607   -0.770
MEH           HBDA   H    H        0.000     6.728   -4.688    0.694
MEH           CAD    C    CH2      0.000     4.649   -5.112    0.368
MEH           HAD    H    H        0.000     4.578   -5.305   -0.704
MEH           HADA   H    H        0.000     4.615   -4.117    0.815
MEH           C3D    C    CR5      0.000     3.174   -4.966    0.026
MEH           C2D    C    CR5      0.000     2.275   -6.011    0.023
MEH           CMD    C    CH3      0.000     2.547   -7.456    0.366
MEH           HMDB   H    H        0.000     3.579   -7.598    0.549
MEH           HMDA   H    H        0.000     2.246   -8.070   -0.444
MEH           HMD    H    H        0.000     1.996   -7.721    1.230
MEH           C4D    C    CR5      0.000     2.419   -3.874   -0.416
MEH           CHA    C    C1       0.000     2.955   -2.503   -0.476
MEH           HHA    H    H        0.000     4.004   -2.385   -0.320
MEH           ND     N    NR5      0.000     1.098   -4.116   -0.520
MEH           C1D    C    CR5      0.000     1.074   -5.453   -0.377
MEH           CHD    C    C1       0.000    -0.154   -6.263   -0.428
MEH           HHD    H    H        0.000    -0.055   -7.314   -0.261
MEH           FE     FE   FE       0.000    -0.441   -2.869   -1.227
MEH           NB     N    NR5      0.000    -1.982   -1.592   -0.509
MEH           C4B    C    CR5      0.000    -3.304   -1.829   -0.323
MEH           C3B    C    CR5      0.000    -4.066   -0.739    0.087
MEH           CAB    C    CH2      0.000    -5.532   -0.710    0.473
MEH           HAB    H    H        0.000    -5.782    0.242    0.944
MEH           CBB    C    CH3      0.000    -6.416   -0.921   -0.740
MEH           HBBA   H    H        0.000    -6.409   -0.043   -1.333
MEH           HBB    H    H        0.000    -7.407   -1.118   -0.422
MEH           C2B    C    CR5      0.000    -3.184    0.311    0.075
MEH           CMB    C    CH3      0.000    -3.451    1.762    0.398
MEH           HMBB   H    H        0.000    -4.481    1.912    0.590
MEH           HMBA   H    H        0.000    -3.160    2.362   -0.425
MEH           HMB    H    H        0.000    -2.890    2.041    1.252
MEH           C1B    C    CR5      0.000    -1.991   -0.245   -0.361
MEH           CHB    C    C1       0.000    -0.796    0.599   -0.442
MEH           HHB    H    H        0.000    -0.907    1.650   -0.279
MEH           NC     N    NR5      0.000    -1.767   -4.384   -0.565
MEH           C4C    C    CR5      0.000    -1.504   -5.690   -0.348
MEH           C3C    C    CR5      0.000    -2.580   -6.464    0.081
MEH           CAC    C    CH2      0.000    -2.590   -7.937    0.441
MEH           HAC    H    H        0.000    -1.762   -8.463   -0.035
MEH           CBC    C    CH3      0.000    -2.522   -8.123    1.944
MEH           HBCA   H    H        0.000    -2.666   -9.147    2.178
MEH           HBC    H    H        0.000    -1.571   -7.814    2.293
MEH           C2C    C    CR5      0.000    -3.637   -5.577    0.123
MEH           CMC    C    CH3      0.000    -5.089   -5.828    0.476
MEH           HMCB   H    H        0.000    -4.695   -6.606    1.067
MEH           HMCA   H    H        0.000    -5.184   -5.721   -0.574
```

```
MEH           HMC    H    H        0.000     -5.754   -6.616    0.233
MEH           C1C    C    CR5      0.000     -3.088   -4.380   -0.320
MEH           CHC    C    C1       0.000     -3.897   -3.168   -0.355
MEH           HHC    H    H        0.000     -4.947   -3.270   -0.163
MEH           NA     N    NR5      0.000      0.799   -1.266   -0.613
MEH           C1A    C    CR5      0.000      2.135   -1.281   -0.410
MEH           C4A    C    CR5      0.000      0.547    0.037   -0.398
MEH           C3A    C    CR5      0.000      1.637    0.810   -0.016
MEH           CMA    C    CH3      0.000      1.673    2.278    0.340
MEH           HMAB   H    H        0.000      0.916    2.800   -0.185
MEH           HMAA   H    H        0.000      1.509    2.386    1.380
MEH           HMA    H    H        0.000      2.616    2.689    0.092
MEH           C2A    C    CR5      0.000      2.692   -0.070   -0.006
MEH           CAA    C    CH2      0.000      4.135    0.260    0.316
MEH           HAA    H    H        0.000      4.164    0.876    1.218
MEH           HAAA   H    H        0.000      4.709   -0.645    0.523
MEH           CBA    C    CH2      0.000      4.782    1.030   -0.830
MEH           HBA    H    H        0.000      4.144    1.857   -1.144
MEH           HBAA   H    H        0.000      4.912    0.365   -1.687
MEH           CGA    C    C        0.000      6.129    1.559   -0.396
MEH           O1A    O    OC      -0.500      6.332    2.791   -0.456
MEH           O2A    O    OC      -0.500      6.989    0.747    0.009
MEH           HABA   H    H        0.000     -5.727   -1.498    1.202
MEH           HBBB   H    H        0.000     -6.062   -1.733   -1.318
MEH           HACA   H    H        0.000     -3.514   -8.386    0.071
MEH           HBCB   H    H        0.000     -3.271   -7.546    2.418
loop_
_chem_comp_tree.comp_id
_chem_comp_tree.atom_id
_chem_comp_tree.atom_back
_chem_comp_tree.atom_forward
_chem_comp_tree.connect_type
MEH      O2D    CGD    .      .
MEH      CGD    CBD    O2D    .
MEH      O1D    CGD    .      .
MEH      CBD    CAD    CGD    .
MEH      HBD    CBD    .      .
MEH      HBDA   CBD    .      .
MEH      CAD    C3D    CBD    .
MEH      HAD    CAD    .      .
MEH      HADA   CAD    .      .
MEH      C3D    C4D    CAD    .
MEH      C2D    C1D    CMD    .
MEH      CMD    C2D    HMDB   .
MEH      HMDB   CMD    .      END
MEH      HMDA   CMD    .      .
MEH      HMD    CMD    .      .
MEH      C4D    ND     C3D    .
MEH      CHA    C1A    HHA    .
MEH      HHA    CHA    .      .
MEH      ND     FE     C4D    .
MEH      C1D    CHD    C2D    .
MEH      CHD    C4C    C1D    .
MEH      HHD    CHD    .      .
MEH      FE     NA     NC     .
MEH      NB     C1B    .      .
MEH      C4B    CHC    .      .
MEH      C3B    C2B    CAB    .
MEH      CAB    C3B    CBB    .
MEH      HAB    CAB    .      .
MEH      CBB    CAB    HBBA   .
```

```
MEH      HBBA   CBB    .      .
MEH      HBB    CBB    .      .
MEH      C2B    C1B    C3B    .
MEH      CMB    C2B    HMBB   .
MEH      HMBB   CMB    .      .
MEH      HMBA   CMB    .      .
MEH      HMB    CMB    .      .
MEH      C1B    CHB    NB     .
MEH      CHB    C4A    C1B    .
MEH      HHB    CHB    .      .
MEH      NC     FE     C1C    .
MEH      C4C    C3C    CHD    .
MEH      C3C    C2C    C4C    .
MEH      CAC    C3C    CBC    .
MEH      HAC    CAC    .      .
MEH      CBC    CAC    HBCA   .
MEH      HBCA   CBC    .      .
MEH      HBC    CBC    .      .
MEH      C2C    C1C    C3C    .
MEH      CMC    C2C    HMCB   .
MEH      HMCB   CMC    .      .
MEH      HMCA   CMC    .      .
MEH      HMC    CMC    .      .
MEH      C1C    NC     C2C    .
MEH      CHC    C1C    C4B    .
MEH      HHC    CHC    .      .
MEH      NA     C4A    FE     .
MEH      C1A    C2A    CHA    .
MEH      C4A    C3A    NA     .
MEH      C3A    C2A    C4A    .
MEH      CMA    C3A    HMAB   .
MEH      HMAB   CMA    .      .
MEH      HMAA   CMA    .      .
MEH      HMA    CMA    .      .
MEH      C2A    CAA    C3A    .
MEH      CAA    CBA    C2A    .
MEH      HAA    CAA    .      .
MEH      HAAA   CAA    .      .
MEH      CBA    CGA    CAA    .
MEH      HBA    CBA    .      .
MEH      HBAA   CBA    .      .
MEH      CGA    O2A    CBA    .
MEH      O1A    CGA    .      .
MEH      O2A    n/a    CGA    START
MEH      HABA   CAB    .      .
MEH      HBBB   CBB    .      .
MEH      HACA   CAC    .      .
MEH      HBCB   CBC    .      .
MEH      C3D    C2D    .    ADD
MEH      C4D    CHA    .    ADD
MEH      ND     C1D    .    ADD
MEH      FE     NB     .    ADD
MEH      NB     C4B    .    ADD
MEH      C4B    C3B    .    ADD
MEH      NC     C4C    .    ADD
MEH      NA     C1A    .    ADD
loop_
_chem_comp_bond.comp_id
_chem_comp_bond.atom_id_1
_chem_comp_bond.atom_id_2
_chem_comp_bond.type
```

```
_chem_comp_bond.value_dist
_chem_comp_bond.value_dist_esd
MEH      C1A    CHA      deloc       1.393    0.020
MEH      CHA    C4D      deloc       1.393    0.020
MEH      C1B    CHB      deloc       1.393    0.020
MEH      CHB    C4A      deloc       1.393    0.020
MEH      C1C    CHC      deloc       1.393    0.020
MEH      CHC    C4B      deloc       1.393    0.020
MEH      C1D    CHD      deloc       1.393    0.020
MEH      CHD    C4C      deloc       1.393    0.020
MEH      CHA    HHA      single      1.077    0.020
MEH      CHB    HHB      single      1.077    0.020
MEH      CHC    HHC      single      1.077    0.020
MEH      CHD    HHD      single      1.077    0.020
MEH      CGD    O2D      deloc       1.250    0.020
MEH      CGD    O1D      deloc       1.250    0.020
MEH      CGD    CBD      single      1.510    0.020
MEH      CBD    HBD      single      1.092    0.020
MEH      CBD    HBDA     single      1.092    0.020
MEH      CBD    CAD      single      1.524    0.020
MEH      CAD    HAD      single      1.092    0.020
MEH      CAD    HADA     single      1.092    0.020
MEH      CAD    C3D      single      1.510    0.020
MEH      C3D    C2D      aromatic    1.390    0.020
MEH      C3D    C4D      aromatic    1.390    0.020
MEH      C2D    CMD      single      1.506    0.020
MEH      C2D    C1D      aromatic    1.390    0.020
MEH      CMD    HMDB     single      1.059    0.020
MEH      CMD    HMDA     single      1.059    0.020
MEH      CMD    HMD      single      1.059    0.020
MEH      C4D    ND       aromatic    1.337    0.020
MEH      ND     C1D      aromatic    1.337    0.020
MEH      NB     C4B      aromatic    1.337    0.020
MEH      NB     C1B      aromatic    1.337    0.020
MEH      C4B    C3B      aromatic    1.390    0.020
MEH      C3B    CAB      single      1.510    0.020
MEH      C3B    C2B      aromatic    1.390    0.020
MEH      CAB    HAB      single      1.092    0.020
MEH      CAB    CBB      single      1.513    0.020
MEH      CAB    HABA     single      1.092    0.020
MEH      CBB    HBBA     single      1.059    0.020
MEH      CBB    HBB      single      1.059    0.020
MEH      CBB    HBBB     single      1.059    0.020
MEH      C2B    CMB      single      1.506    0.020
MEH      C2B    C1B      aromatic    1.390    0.020
MEH      CMB    HMBB     single      1.059    0.020
MEH      CMB    HMBA     single      1.059    0.020
MEH      CMB    HMB      single      1.059    0.020
MEH      NC     C4C      aromatic    1.337    0.020
MEH      NC     C1C      aromatic    1.337    0.020
MEH      C4C    C3C      aromatic    1.390    0.020
MEH      C3C    CAC      single      1.510    0.020
MEH      C3C    C2C      aromatic    1.390    0.020
MEH      CAC    HAC      single      1.092    0.020
MEH      CAC    CBC      single      1.513    0.020
MEH      CAC    HACA     single      1.092    0.020
MEH      CBC    HBCA     single      1.059    0.020
MEH      CBC    HBC      single      1.059    0.020
MEH      CBC    HBCB     single      1.059    0.020
MEH      C2C    CMC      single      1.506    0.020
MEH      C2C    C1C      aromatic    1.390    0.020
```

```
MEH      CMC    HMCB      single      1.059    0.020
MEH      CMC    HMCA      single      1.059    0.020
MEH      CMC    HMC       single      1.059    0.020
MEH      NA     C1A       aromatic    1.337    0.020
MEH      NA     C4A       aromatic    1.337    0.020
MEH      C1A    C2A       aromatic    1.390    0.020
MEH      C4A    C3A       aromatic    1.390    0.020
MEH      C3A    CMA       single      1.506    0.020
MEH      C3A    C2A       aromatic    1.390    0.020
MEH      CMA    HMAB      single      1.059    0.020
MEH      CMA    HMAA      single      1.059    0.020
MEH      CMA    HMA       single      1.059    0.020
MEH      C2A    CAA       single      1.510    0.020
MEH      CAA    HAA       single      1.092    0.020
MEH      CAA    HAAA      single      1.092    0.020
MEH      CAA    CBA       single      1.524    0.020
MEH      CBA    HBA       single      1.092    0.020
MEH      CBA    HBAA      single      1.092    0.020
MEH      CBA    CGA       single      1.510    0.020
MEH      CGA    O1A       deloc       1.250    0.020
MEH      CGA    O2A       deloc       1.250    0.020
MEH      FE     NA        metal       2.090    0.100
MEH      FE     NB        metal       2.090    0.100
MEH      FE     NC        metal       2.090    0.100
MEH      FE     ND        metal       2.090    0.100
loop_
_chem_comp_angle.comp_id
_chem_comp_angle.atom_id_1
_chem_comp_angle.atom_id_2
_chem_comp_angle.atom_id_3
_chem_comp_angle.value_angle
_chem_comp_angle.value_angle_esd
MEH      O2D    CGD    O1D     123.000    3.000
MEH      O2D    CGD    CBD     118.500    3.000
MEH      CGD    CBD    HBD     109.470    3.000
MEH      CGD    CBD    HBDA    109.470    3.000
MEH      CGD    CBD    CAD     109.470    3.000
MEH      O1D    CGD    CBD     118.500    3.000
MEH      CBD    CAD    HAD     109.470    3.000
MEH      CBD    CAD    HADA    109.470    3.000
MEH      CBD    CAD    C3D     109.470    3.000
MEH      HBD    CBD    HBDA    107.900    3.000
MEH      HBD    CBD    CAD     109.470    3.000
MEH      HBDA   CBD    CAD     109.470    3.000
MEH      CAD    C3D    C2D     126.000    3.000
MEH      CAD    C3D    C4D     126.000    3.000
MEH      HAD    CAD    HADA    107.900    3.000
MEH      HAD    CAD    C3D     109.470    3.000
MEH      HADA   CAD    C3D     109.470    3.000
MEH      C3D    C2D    CMD     126.000    3.000
MEH      C3D    C2D    C1D     108.000    3.000
MEH      C3D    C4D    CHA     117.000    3.000
MEH      C3D    C4D    ND      108.000    3.000
MEH      C2D    C3D    C4D     108.000    3.000
MEH      C2D    CMD    HMDB    109.470    3.000
MEH      C2D    CMD    HMDA    109.470    3.000
MEH      C2D    CMD    HMD     109.470    3.000
MEH      C2D    C1D    ND      108.000    3.000
MEH      C2D    C1D    CHD     117.000    3.000
MEH      CMD    C2D    C1D     126.000    3.000
MEH      HMDB   CMD    HMDA    109.470    3.000
```

MEH	HMDB	CMD	HMD	109.470	3.000
MEH	HMDA	CMD	HMD	109.470	3.000
MEH	C4D	CHA	HHA	120.000	3.000
MEH	C4D	CHA	C1A	120.000	3.000
MEH	C4D	ND	C1D	108.000	3.000
MEH	CHA	C4D	ND	108.000	3.000
MEH	CHA	C1A	NA	108.000	3.000
MEH	CHA	C1A	C2A	117.000	3.000
MEH	HHA	CHA	C1A	120.000	3.000
MEH	ND	C1D	CHD	108.000	3.000
MEH	C1D	CHD	HHD	120.000	3.000
MEH	C1D	CHD	C4C	120.000	3.000
MEH	CHD	C4C	NC	108.000	3.000
MEH	CHD	C4C	C3C	117.000	3.000
MEH	HHD	CHD	C4C	120.000	3.000
MEH	NB	C4B	C3B	108.000	3.000
MEH	NB	C4B	CHC	108.000	3.000
MEH	NB	C1B	C2B	108.000	3.000
MEH	NB	C1B	CHB	108.000	3.000
MEH	C4B	NB	C1B	108.000	3.000
MEH	C4B	C3B	CAB	126.000	3.000
MEH	C4B	C3B	C2B	108.000	3.000
MEH	C4B	CHC	C1C	120.000	3.000
MEH	C4B	CHC	HHC	120.000	3.000
MEH	C3B	C4B	CHC	117.000	3.000
MEH	C3B	CAB	HAB	109.470	3.000
MEH	C3B	CAB	CBB	109.470	3.000
MEH	C3B	CAB	HABA	109.470	3.000
MEH	C3B	C2B	CMB	126.000	3.000
MEH	C3B	C2B	C1B	108.000	3.000
MEH	CAB	C3B	C2B	126.000	3.000
MEH	CAB	CBB	HBBA	109.470	3.000
MEH	CAB	CBB	HBB	109.470	3.000
MEH	CAB	CBB	HBBB	109.470	3.000
MEH	HAB	CAB	CBB	109.470	3.000
MEH	HAB	CAB	HABA	107.900	3.000
MEH	CBB	CAB	HABA	109.470	3.000
MEH	HBBA	CBB	HBB	109.470	3.000
MEH	HBBA	CBB	HBBB	109.470	3.000
MEH	HBB	CBB	HBBB	109.470	3.000
MEH	C2B	CMB	HMBB	109.470	3.000
MEH	C2B	CMB	HMBA	109.470	3.000
MEH	C2B	CMB	HMB	109.470	3.000
MEH	C2B	C1B	CHB	117.000	3.000
MEH	CMB	C2B	C1B	126.000	3.000
MEH	HMBB	CMB	HMBA	109.470	3.000
MEH	HMBB	CMB	HMB	109.470	3.000
MEH	HMBA	CMB	HMB	109.470	3.000
MEH	C1B	CHB	HHB	120.000	3.000
MEH	C1B	CHB	C4A	120.000	3.000
MEH	CHB	C4A	NA	108.000	3.000
MEH	CHB	C4A	C3A	117.000	3.000
MEH	HHB	CHB	C4A	120.000	3.000
MEH	NC	C4C	C3C	108.000	3.000
MEH	NC	C1C	C2C	108.000	3.000
MEH	NC	C1C	CHC	108.000	3.000
MEH	C4C	NC	C1C	108.000	3.000
MEH	C4C	C3C	CAC	126.000	3.000
MEH	C4C	C3C	C2C	108.000	3.000
MEH	C3C	CAC	HAC	109.470	3.000
MEH	C3C	CAC	CBC	109.470	3.000

MEH	C3C	CAC	HACA	109.470	3.000
MEH	C3C	C2C	CMC	126.000	3.000
MEH	C3C	C2C	C1C	108.000	3.000
MEH	CAC	C3C	C2C	126.000	3.000
MEH	CAC	CBC	HBCA	109.470	3.000
MEH	CAC	CBC	HBC	109.470	3.000
MEH	CAC	CBC	HBCB	109.470	3.000
MEH	HAC	CAC	CBC	109.470	3.000
MEH	HAC	CAC	HACA	107.900	3.000
MEH	CBC	CAC	HACA	109.470	3.000
MEH	HBCA	CBC	HBC	109.470	3.000
MEH	HBCA	CBC	HBCB	109.470	3.000
MEH	HBC	CBC	HBCB	109.470	3.000
MEH	C2C	CMC	HMCB	109.470	3.000
MEH	C2C	CMC	HMCA	109.470	3.000
MEH	C2C	CMC	HMC	109.470	3.000
MEH	C2C	C1C	CHC	117.000	3.000
MEH	CMC	C2C	C1C	126.000	3.000
MEH	HMCB	CMC	HMCA	109.470	3.000
MEH	HMCB	CMC	HMC	109.470	3.000
MEH	HMCA	CMC	HMC	109.470	3.000
MEH	C1C	CHC	HHC	120.000	3.000
MEH	NA	C1A	C2A	108.000	3.000
MEH	NA	C4A	C3A	108.000	3.000
MEH	C1A	NA	C4A	108.000	3.000
MEH	C1A	C2A	C3A	108.000	3.000
MEH	C1A	C2A	CAA	126.000	3.000
MEH	C4A	C3A	CMA	126.000	3.000
MEH	C4A	C3A	C2A	108.000	3.000
MEH	C3A	CMA	HMAB	109.470	3.000
MEH	C3A	CMA	HMAA	109.470	3.000
MEH	C3A	CMA	HMA	109.470	3.000
MEH	C3A	C2A	CAA	126.000	3.000
MEH	CMA	C3A	C2A	126.000	3.000
MEH	HMAB	CMA	HMAA	109.470	3.000
MEH	HMAB	CMA	HMA	109.470	3.000
MEH	HMAA	CMA	HMA	109.470	3.000
MEH	C2A	CAA	HAA	109.470	3.000
MEH	C2A	CAA	HAAA	109.470	3.000
MEH	C2A	CAA	CBA	109.470	3.000
MEH	CAA	CBA	HBA	109.470	3.000
MEH	CAA	CBA	HBAA	109.470	3.000
MEH	CAA	CBA	CGA	109.470	3.000
MEH	HAA	CAA	HAAA	107.900	3.000
MEH	HAA	CAA	CBA	109.470	3.000
MEH	HAAA	CAA	CBA	109.470	3.000
MEH	CBA	CGA	O1A	118.500	3.000
MEH	CBA	CGA	O2A	118.500	3.000
MEH	HBA	CBA	HBAA	107.900	3.000
MEH	HBA	CBA	CGA	109.470	3.000
MEH	HBAA	CBA	CGA	109.470	3.000
MEH	O1A	CGA	O2A	123.000	3.000
MEH	FE	NA	C1A	126.000	7.500
MEH	FE	NA	C4A	126.000	7.500
MEH	FE	NB	C1B	126.000	7.500
MEH	FE	NB	C4B	126.000	7.500
MEH	FE	NC	C1C	126.000	7.500
MEH	FE	NC	C4C	126.000	7.500
MEH	FE	ND	C1D	126.000	7.500
MEH	FE	ND	C4D	126.000	7.500
MEH	NA	FE	NB	90.000	7.500

```
MEH      NB     FE     NC       90.000    7.500
MEH      NC     FE     ND       90.000    7.500
MEH      ND     FE     NA       90.000    7.500
loop_
_chem_comp_plane_atom.comp_id
_chem_comp_plane_atom.plane_id
_chem_comp_plane_atom.atom_id
_chem_comp_plane_atom.dist_esd
MEH      plan-1A          C4D       0.020
MEH      plan-1A          CHA       0.020
MEH      plan-1A          HHA       0.020
MEH      plan-1A          C1A       0.020
MEH      plan-2A          CHA       0.020
MEH      plan-2A          C1A       0.020
MEH      plan-2A          C2A       0.020
MEH      plan-2A          NA        0.020
MEH      plan-3A          NA        0.020
MEH      plan-3A          C1A       0.020
MEH      plan-3A          C2A       0.020
MEH      plan-3A          C3A       0.020
MEH      plan-3A          C4A       0.020
MEH      plan-4A          C1A       0.020
MEH      plan-4A          C2A       0.020
MEH      plan-4A          CAA       0.020
MEH      plan-4A          C3A       0.020
MEH      plan-5A          C2A       0.020
MEH      plan-5A          C3A       0.020
MEH      plan-5A          CMA       0.020
MEH      plan-5A          C4A       0.020
MEH      plan-6A          CBA       0.020
MEH      plan-6A          CGA       0.020
MEH      plan-6A          O1A       0.020
MEH      plan-6A          O2A       0.020
MEH      plan-7A          NA        0.020
MEH      plan-7A          C4A       0.020
MEH      plan-7A          C3A       0.020
MEH      plan-7A          CHB       0.020
MEH      plan-1B          C4A       0.020
MEH      plan-1B          CHB       0.020
MEH      plan-1B          HHB       0.020
MEH      plan-1B          C1B       0.020
MEH      plan-2B          CHB       0.020
MEH      plan-2B          C1B       0.020
MEH      plan-2B          C2B       0.020
MEH      plan-2B          NB        0.020
MEH      plan-3B          NB        0.020
MEH      plan-3B          C1B       0.020
MEH      plan-3B          C2B       0.020
MEH      plan-3B          C3B       0.020
MEH      plan-3B          C4B       0.020
MEH      plan-4B          C1B       0.020
MEH      plan-4B          C2B       0.020
MEH      plan-4B          CMB       0.020
MEH      plan-4B          C3B       0.020
MEH      plan-5B          C2B       0.020
MEH      plan-5B          C3B       0.020
MEH      plan-5B          CAB       0.020
MEH      plan-5B          C4B       0.020
MEH      plan-7B          NB        0.020
MEH      plan-7B          C4B       0.020
MEH      plan-7B          C3B       0.020
```

```
MEH      plan-7B           CHC       0.020
MEH      plan-1C           C4B       0.020
MEH      plan-1C           CHC       0.020
MEH      plan-1C           HHC       0.020
MEH      plan-1C           C1C       0.020
MEH      plan-2C           CHC       0.020
MEH      plan-2C           C1C       0.020
MEH      plan-2C           C2C       0.020
MEH      plan-2C           NC        0.020
MEH      plan-3C           NC        0.020
MEH      plan-3C           C1C       0.020
MEH      plan-3C           C2C       0.020
MEH      plan-3C           C3C       0.020
MEH      plan-3C           C4C       0.020
MEH      plan-4C           C1C       0.020
MEH      plan-4C           C2C       0.020
MEH      plan-4C           CMC       0.020
MEH      plan-4C           C3C       0.020
MEH      plan-5C           C2C       0.020
MEH      plan-5C           C3C       0.020
MEH      plan-5C           CAC       0.020
MEH      plan-5C           C4C       0.020
MEH      plan-7C           NC        0.020
MEH      plan-7C           C4C       0.020
MEH      plan-7C           C3C       0.020
MEH      plan-7C           CHD       0.020
MEH      plan-1D           C4C       0.020
MEH      plan-1D           CHD       0.020
MEH      plan-1D           HHD       0.020
MEH      plan-1D           C1D       0.020
MEH      plan-2D           CHD       0.020
MEH      plan-2D           C1D       0.020
MEH      plan-2D           C2D       0.020
MEH      plan-2D           ND        0.020
MEH      plan-3D           ND        0.020
MEH      plan-3D           C1D       0.020
MEH      plan-3D           C2D       0.020
MEH      plan-3D           C3D       0.020
MEH      plan-3D           C4D       0.020
MEH      plan-4D           C1D       0.020
MEH      plan-4D           C2D       0.020
MEH      plan-4D           CMD       0.020
MEH      plan-4D           C3D       0.020
MEH      plan-5D           C2D       0.020
MEH      plan-5D           C3D       0.020
MEH      plan-5D           CAD       0.020
MEH      plan-5D           C4D       0.020
MEH      plan-6D           CBD       0.020
MEH      plan-6D           CGD       0.020
MEH      plan-6D           O1D       0.020
MEH      plan-6D           O2D       0.020
MEH      plan-7D           ND        0.020
MEH      plan-7D           C4D       0.020
MEH      plan-7D           C3D       0.020
MEH      plan-7D           CHA       0.020
#
data_comp_No
#
loop_
_chem_comp_atom.comp_id
_chem_comp_atom.atom_id
```

```
_chem_comp_atom.type_symbol
_chem_comp_atom.type_energy
_chem_comp_atom.partial_charge
No            N      N    N         0.000
No            O      O    O         0.000
loop_
_chem_comp_tree.comp_id
_chem_comp_tree.atom_id
_chem_comp_tree.atom_back
_chem_comp_tree.atom_forward
_chem_comp_tree.connect_type
No       N      n/a    O      START
No       O      N      .      END
loop_
_chem_comp_bond.comp_id
_chem_comp_bond.atom_id_1
_chem_comp_bond.atom_id_2
_chem_comp_bond.type
_chem_comp_bond.value_dist
_chem_comp_bond.value_dist_esd
No       O      N         triple      1.15    0.020
#loop_
# -----------------------------------------------------
#
# --- DESCRIPTION OF MODIFICATIONS ---
#
data_mod_CYS-HG
#
loop_
_chem_mod_atom.mod_id
_chem_mod_atom.function
_chem_mod_atom.atom_id
_chem_mod_atom.new_atom_id
_chem_mod_atom.new_type_symbol
_chem_mod_atom.new_type_energy
_chem_mod_atom.new_partial_charge
CYS-HG   change   SG     .      .    S2      0.000
CYS-HG   delete   HG     .      .    .       0.000
#
data_mod_MEH-HABA
#
loop_
_chem_mod_atom.mod_id
_chem_mod_atom.function
_chem_mod_atom.atom_id
_chem_mod_atom.new_atom_id
_chem_mod_atom.new_type_symbol
_chem_mod_atom.new_type_energy
_chem_mod_atom.new_partial_charge
MEH-HABA change   CAB    .      .    CH1     0.000
MEH-HABA delete   HABA   .      .    .       0.000
loop_
_chem_mod_bond.mod_id
_chem_mod_bond.function
_chem_mod_bond.atom_id_1
_chem_mod_bond.atom_id_2
_chem_mod_bond.new_type
_chem_mod_bond.new_value_dist
_chem_mod_bond.new_value_dist_esd
MEH-HABA change   C3B     CAB       .           1.480    0.020
#
```

```
data_mod_MEH-HACA
#
loop_
_chem_mod_atom.mod_id
_chem_mod_atom.function
_chem_mod_atom.atom_id
_chem_mod_atom.new_atom_id
_chem_mod_atom.new_type_symbol
_chem_mod_atom.new_type_energy
_chem_mod_atom.new_partial_charge
MEH-HACA change   CAC    .      .    CH1     0.000
MEH-HACA delete   HACA   .      .    .       0.000
loop_
_chem_mod_bond.mod_id
_chem_mod_bond.function
_chem_mod_bond.atom_id_1
_chem_mod_bond.atom_id_2
_chem_mod_bond.new_type
_chem_mod_bond.new_value_dist
_chem_mod_bond.new_value_dist_esd
MEH-HACA change   C3C     CAC       .           1.480    0.020
#
data_mod_HIS-HE2
#
loop_
_chem_mod_atom.mod_id
_chem_mod_atom.function
_chem_mod_atom.atom_id
_chem_mod_atom.new_atom_id
_chem_mod_atom.new_type_symbol
_chem_mod_atom.new_type_energy
_chem_mod_atom.new_partial_charge
HIS-HE2  delete   HE2    .      .    .       0.000
# ------------------------------------------------------
#
# --- DESCRIPTION OF LINKS ---
#
data_link_CYS-MEHB
#
loop_
_chem_link_bond.link_id
_chem_link_bond.atom_1_comp_id
_chem_link_bond.atom_id_1
_chem_link_bond.atom_2_comp_id
_chem_link_bond.atom_id_2
_chem_link_bond.type
_chem_link_bond.value_dist
_chem_link_bond.value_dist_esd
CYS-MEHB 1 SG      2 CAB       single      1.765    0.020
loop_
_chem_link_angle.link_id
_chem_link_angle.atom_1_comp_id
_chem_link_angle.atom_id_1
_chem_link_angle.atom_2_comp_id
_chem_link_angle.atom_id_2
_chem_link_angle.atom_3_comp_id
_chem_link_angle.atom_id_3
_chem_link_angle.value_angle
_chem_link_angle.value_angle_esd
CYS-MEHB 2 CAB     1 SG      1 CB      109.470    3.000
CYS-MEHB 2 HAB     2 CAB     1 SG      109.500    3.000
```

```
CYS-MEHB 2 CBB     2 CAB     1 SG      109.500    3.000
CYS-MEHB 2 C3B     2 CAB     1 SG      109.500    3.000
loop_
_chem_link_chir.link_id
_chem_link_chir.atom_centre_comp_id
_chem_link_chir.atom_id_centre
_chem_link_chir.atom_1_comp_id
_chem_link_chir.atom_id_1
_chem_link_chir.atom_2_comp_id
_chem_link_chir.atom_id_2
_chem_link_chir.atom_3_comp_id
_chem_link_chir.atom_id_3
_chem_link_chir.volume_sign
CYS-MEHB   2 CAB     2 C3B     2 CBB     1 SG      both
#
data_link_CYS-MEHC
#
loop_
_chem_link_bond.link_id
_chem_link_bond.atom_1_comp_id
_chem_link_bond.atom_id_1
_chem_link_bond.atom_2_comp_id
_chem_link_bond.atom_id_2
_chem_link_bond.type
_chem_link_bond.value_dist
_chem_link_bond.value_dist_esd
CYS-MEHC 1 SG      2 CAC       single      1.765    0.020
loop_
_chem_link_angle.link_id
_chem_link_angle.atom_1_comp_id
_chem_link_angle.atom_id_1
_chem_link_angle.atom_2_comp_id
_chem_link_angle.atom_id_2
_chem_link_angle.atom_3_comp_id
_chem_link_angle.atom_id_3
_chem_link_angle.value_angle
_chem_link_angle.value_angle_esd
CYS-MEHC 2 CAC     1 SG      1 CB      109.470    3.000
CYS-MEHC 2 HAC     2 CAC     1 SG      109.500    3.000
CYS-MEHC 2 CBC     2 CAC     1 SG      109.500    3.000
CYS-MEHC 2 C3C     2 CAC     1 SG      109.500    3.000
loop_
_chem_link_chir.link_id
_chem_link_chir.atom_centre_comp_id
_chem_link_chir.atom_id_centre
_chem_link_chir.atom_1_comp_id
_chem_link_chir.atom_id_1
_chem_link_chir.atom_2_comp_id
_chem_link_chir.atom_id_2
_chem_link_chir.atom_3_comp_id
_chem_link_chir.atom_id_3
_chem_link_chir.volume_sign
CYS-MEHC   2 CAC     2 C3C     2 CBC     1 SG      both
#
data_link_HIS-MEH
#
loop_
_chem_link_bond.link_id
_chem_link_bond.atom_1_comp_id
_chem_link_bond.atom_id_1
_chem_link_bond.atom_2_comp_id
```

```
_chem_link_bond.atom_id_2
_chem_link_bond.type
_chem_link_bond.value_dist
_chem_link_bond.value_dist_esd
HIS-MEH  1 NE2     2 FE        metal       1.935    0.120
# -------------------------------------------------------
```

HX2 restraints

Restraints used for the RT fresh and RT aged structures. The restraints were made in JLigand based on the SMILES string of the proposed haem-lysine structure. The haem and Lys78 monomers were deleted in Coot and replaced by the HX2 monomer. The iron atom (Fe201) was added back in.

```
data_xyz

loop_
_struct_conn.id
_struct_conn.conn_type_id
_struct_conn.ptnr1_label_asym_id
_struct_conn.ptnr1_label_seq_id
_struct_conn.ptnr1_label_comp_id
_struct_conn.ptnr1_label_atom_id
_struct_conn.ptnr2_label_asym_id
_struct_conn.ptnr2_label_seq_id
_struct_conn.ptnr2_label_comp_id
_struct_conn.ptnr2_label_atom_id
_struct_conn.ccp4_link_id
   1 covalent A   3 HX2 .    A   5 CYS .    HX2-CYS
   2 covalent A   9 CYS .    A   11 HX2 .    CYS-HX2

loop_
_atom_site.id
_atom_site.type_symbol
_atom_site.label_atom_id
_atom_site.label_alt_id
_atom_site.label_comp_id
_atom_site.label_asym_id
_atom_site.auth_seq_id
_atom_site.Cartn_x
_atom_site.Cartn_y
_atom_site.Cartn_z
_atom_site.occupancy
_atom_site.B_iso_or_equiv
   1 C  CBC   . HX2 A   1    -6.641    -7.573     0.719  1.00 .
   2 C  CAC   . HX2 A   1    -7.081    -8.936     0.230  1.00 .
   3 S  S1    . HX2 A   1    -8.068    -9.810     1.479  1.00 .
   4 C  C3C   . HX2 A   1    -5.909    -9.768    -0.200  1.00 .
   5 C  C4C   . HX2 A   1    -5.630   -11.153     0.091  1.00 .
   6 N  NC    . HX2 A   1    -4.437   -11.454    -0.515  1.00 .
   7 C  C1C   . HX2 A   1    -3.950   -10.365    -1.191  1.00 .
   8 C  CHC   . HX2 A   1    -2.748   -10.327    -1.875  1.00 .
   9 C  C4B   . HX2 A   1    -1.533   -10.269    -1.260  1.00 .
  10 N  NB    . HX2 A   1    -0.458   -11.076    -1.501  1.00 .
  11 C  C1B   . HX2 A   1     0.117   -11.139    -0.261  1.00 .
  12 C  CHB   . HX2 A   1     0.649   -12.275     0.323  1.00 .
  13 C  C4A   . HX2 A   1    -0.030   -13.482     0.375  1.00 .
  14 N  NA    . HX2 A   1    -1.298   -13.719     0.914  1.00 .
  15 C  C1A   . HX2 A   1    -1.933   -14.576     0.071  1.00 .
  16 C  CHA   . HX2 A   1    -3.236   -14.657    -0.431  1.00 .
  17 N  NZ    . HX2 A   1    -3.262   -14.679    -1.782  1.00 .
```

18	C	CE	.	HX2	A	1	-2.139	-14.930	-2.493	1.00	.
19	C	CD	.	HX2	A	1	-1.145	-15.674	-1.924	1.00	.
20	C	C2A	.	HX2	A	1	-0.855	-15.513	-0.446	1.00	.
21	C	CAA	.	HX2	A	1	-0.808	-16.870	0.292	1.00	.
22	C	CBA	.	HX2	A	1	0.572	-17.295	0.774	1.00	.
23	C	CGA	.	HX2	A	1	1.609	-17.386	-0.331	1.00	.
24	O	O2A	.	HX2	A	1	2.377	-18.370	-0.338	1.00	.
25	O	O1A	.	HX2	A	1	1.648	-16.472	-1.181	1.00	.
26	C	C3A	.	HX2	A	1	0.398	-14.697	-0.132	1.00	.
27	C	CMA	.	HX2	A	1	1.641	-15.100	-0.319	1.00	.
28	C	C4D	.	HX2	A	1	-4.485	-14.675	0.334	1.00	.
29	N	ND	.	HX2	A	1	-5.643	-14.166	-0.175	1.00	.
30	C	C1D	.	HX2	A	1	-6.043	-13.391	0.876	1.00	.
31	C	CHD	.	HX2	A	1	-6.366	-12.067	0.831	1.00	.
32	C	C2D	.	HX2	A	1	-5.823	-14.207	2.072	1.00	.
33	C	CMD	.	HX2	A	1	-6.525	-14.092	3.394	1.00	.
34	C	C3D	.	HX2	A	1	-4.846	-15.078	1.697	1.00	.
35	C	CAD	.	HX2	A	1	-4.292	-16.224	2.484	1.00	.
36	C	CBD	.	HX2	A	1	-4.746	-17.656	2.253	1.00	.
37	C	CGD	.	HX2	A	1	-3.670	-18.676	2.566	1.00	.
38	O	O1D	.	HX2	A	1	-3.193	-18.691	3.720	1.00	.
39	O	O2D	.	HX2	A	1	-3.314	-19.452	1.654	1.00	.
40	C	C2B	.	HX2	A	1	-0.044	-9.818	0.324	1.00	.
41	C	CMB	.	HX2	A	1	0.900	-9.109	1.250	1.00	.
42	C	C3B	.	HX2	A	1	-1.225	-9.379	-0.160	1.00	.
43	C	CAB	.	HX2	A	1	-2.097	-8.254	0.325	1.00	.
44	C	CBB	.	HX2	A	1	-1.883	-7.984	1.797	1.00	.
45	S	S2	.	HX2	A	1	-3.854	-8.546	-0.033	1.00	.
46	C	C2C	.	HX2	A	1	-4.874	-9.290	-0.977	1.00	.
47	C	CMC	.	HX2	A	1	-4.720	-7.900	-1.520	1.00	.
48	H	H1	.	HX2	A	1	-5.862	-7.294	0.231	1.00	.
49	H	H21	.	HX2	A	1	-7.349	-6.940	0.584	1.00	.
50	H	H31	.	HX2	A	1	-6.430	-7.623	1.655	1.00	.
51	H	H4	.	HX2	A	1	-7.662	-8.800	-0.561	1.00	.
52	H	H5	.	HX2	A	1	-7.279	-9.790	2.399	1.00	.
53	H	H6	.	HX2	A	1	-4.048	-12.241	-0.490	1.00	.
54	H	H7	.	HX2	A	1	-2.764	-10.326	-2.827	1.00	.
55	H	H8	.	HX2	A	1	1.524	-12.219	0.701	1.00	.
56	H	H9	.	HX2	A	1	-1.633	-13.385	1.654	1.00	.
57	H	H10	.	HX2	A	1	-4.029	-14.526	-2.207	1.00	.
58	H	H11	.	HX2	A	1	-2.033	-14.601	-3.369	1.00	.
59	H	H13	.	HX2	A	1	-1.158	-17.563	-0.307	1.00	.
60	H	H14	.	HX2	A	1	-1.409	-16.826	1.065	1.00	.
61	H	H15	.	HX2	A	1	0.883	-16.651	1.445	1.00	.
62	H	H16	.	HX2	A	1	0.497	-18.172	1.207	1.00	.
63	H	H17	.	HX2	A	1	2.362	-14.530	-0.095	1.00	.
64	H	H18	.	HX2	A	1	1.810	-15.958	-0.676	1.00	.
65	H	H19	.	HX2	A	1	-7.112	-11.768	1.345	1.00	.
66	H	H20	.	HX2	A	1	-7.472	-13.941	3.247	1.00	.
67	H	H211	.	HX2	A	1	-6.405	-14.911	3.899	1.00	.
68	H	H22	.	HX2	A	1	-6.156	-13.350	3.896	1.00	.
69	H	H23	.	HX2	A	1	-3.999	-16.415	1.571	1.00	.
70	H	H24	.	HX2	A	1	-5.235	-16.211	2.740	1.00	.
71	H	H25	.	HX2	A	1	-5.529	-17.853	2.811	1.00	.
72	H	H26	.	HX2	A	1	-5.020	-17.773	1.319	1.00	.
73	H	H27	.	HX2	A	1	0.420	-8.425	1.741	1.00	.
74	H	H28	.	HX2	A	1	1.284	-9.744	1.873	1.00	.
75	H	H29	.	HX2	A	1	1.610	-8.695	0.735	1.00	.
76	H	H30	.	HX2	A	1	-1.829	-7.443	-0.175	1.00	.
77	H	H311	.	HX2	A	1	-1.654	-7.170	2.278	1.00	.
78	H	H32	.	HX2	A	1	-2.699	-7.474	1.655	1.00	.

79	H	H33	.	HX2	A	1	-0.975	-7.853	1.474	1.00	.
80	H	H34	.	HX2	A	1	-4.093	-9.342	0.852	1.00	.
81	H	H35	.	HX2	A	1	-5.512	-7.379	-1.313	1.00	.
82	H	H36	.	HX2	A	1	-3.943	-7.478	-1.118	1.00	.
83	H	H37	.	HX2	A	1	-4.602	-7.938	-2.483	1.00	.
84	N	N	.	HX2	A	1	-2.370	-19.884	-2.666	1.00	.
85	C	CA	.	HX2	A	1	-1.083	-19.140	-2.702	1.00	.
86	C	C	.	HX2	A	1	-0.528	-19.017	-1.274	1.00	.
87	O	O	.	HX2	A	1	0.650	-19.394	-1.084	1.00	.
88	C	CB	.	HX2	A	1	-1.283	-17.760	-3.329	1.00	.
89	C	CG	.	HX2	A	1	-0.379	-16.685	-2.741	1.00	.
90	O	OXT	.	HX2	A	1	-1.293	-18.549	-0.402	1.00	.
91	H	H	.	HX2	A	1	-2.463	-20.297	-1.869	1.00	.
92	H	H2	.	HX2	A	1	-2.386	-20.503	-3.321	1.00	.
93	H	H3	.	HX2	A	1	-3.060	-19.313	-2.783	1.00	.
94	H	HA	.	HX2	A	1	-0.440	-19.656	-3.251	1.00	.
95	H	HB3	.	HX2	A	1	-2.217	-17.491	-3.201	1.00	.
96	H	HB2	.	HX2	A	1	-1.116	-17.825	-4.292	1.00	.
97	H	HG3	.	HX2	A	1	0.300	-17.114	-2.168	1.00	.
98	H	HG2	.	HX2	A	1	0.085	-16.218	-3.477	1.00	.
99	C	CBC	.	HX2	A	3	-16.842	2.710	-1.317	1.00	.
100	C	CAC	.	HX2	A	3	-17.335	1.730	-0.258	1.00	.
101	C	C3C	.	HX2	A	3	-17.246	0.305	-0.729	1.00	.
102	C	C4C	.	HX2	A	3	-16.130	-0.225	-1.456	1.00	.
103	N	NC	.	HX2	A	3	-16.399	-1.550	-1.693	1.00	.
104	C	C1C	.	HX2	A	3	-17.628	-1.888	-1.180	1.00	.
105	C	CHC	.	HX2	A	3	-18.199	-3.152	-1.259	1.00	.
106	C	C4B	.	HX2	A	3	-17.870	-4.129	-2.156	1.00	.
107	N	NB	.	HX2	A	3	-16.983	-5.140	-1.936	1.00	.
108	C	C1B	.	HX2	A	3	-16.232	-5.002	-3.070	1.00	.
109	C	CHB	.	HX2	A	3	-14.851	-4.947	-3.112	1.00	.
110	C	C4A	.	HX2	A	3	-14.106	-4.606	-1.999	1.00	.
111	N	NA	.	HX2	A	3	-12.824	-4.046	-1.955	1.00	.
112	C	C1A	.	HX2	A	3	-12.621	-3.617	-0.682	1.00	.
113	C	CHA	.	HX2	A	3	-12.048	-2.497	-0.074	1.00	.
114	N	NZ	.	HX2	A	3	-11.426	-2.799	1.085	1.00	.
115	C	CE	.	HX2	A	3	-12.176	-3.446	2.004	1.00	.
116	C	CD	.	HX2	A	3	-13.361	-3.967	1.561	1.00	.
117	C	C2A	.	HX2	A	3	-13.317	-4.638	0.199	1.00	.
118	C	CAA	.	HX2	A	3	-12.589	-6.000	0.246	1.00	.
119	C	CBA	.	HX2	A	3	-12.183	-6.559	-1.109	1.00	.
120	C	CGA	.	HX2	A	3	-10.866	-6.015	-1.629	1.00	.
121	O	O2A	.	HX2	A	3	-9.808	-6.469	-1.146	1.00	.
122	O	O1A	.	HX2	A	3	-10.900	-5.137	-2.517	1.00	.
123	C	C3A	.	HX2	A	3	-14.556	-4.752	-0.691	1.00	.
124	C	CMA	.	HX2	A	3	-15.806	-4.969	-0.321	1.00	.
125	C	C4D	.	HX2	A	3	-12.269	-1.125	-0.529	1.00	.
126	N	ND	.	HX2	A	3	-12.847	-0.787	-1.720	1.00	.
127	C	C1D	.	HX2	A	3	-13.713	0.214	-1.355	1.00	.
128	C	CHD	.	HX2	A	3	-14.975	0.459	-1.812	1.00	.
129	C	C2D	.	HX2	A	3	-13.078	0.946	-0.255	1.00	.
130	C	CMD	.	HX2	A	3	-13.488	2.282	0.293	1.00	.
131	C	C3D	.	HX2	A	3	-12.087	0.132	0.194	1.00	.
132	C	CAD	.	HX2	A	3	-11.075	0.477	1.241	1.00	.
133	C	CBD	.	HX2	A	3	-11.691	0.711	2.612	1.00	.
134	C	CGD	.	HX2	A	3	-13.048	1.380	2.535	1.00	.
135	O	O1D	.	HX2	A	3	-13.190	2.488	3.094	1.00	.
136	O	O2D	.	HX2	A	3	-13.958	0.791	1.914	1.00	.
137	C	C2B	.	HX2	A	3	-17.183	-4.795	-4.148	1.00	.
138	C	CMB	.	HX2	A	3	-17.003	-5.140	-5.598	1.00	.
139	C	C3B	.	HX2	A	3	-18.254	-4.224	-3.549	1.00	.

140	C	CAB	.	HX2	A	3	-19.554	-3.794	-4.168	1.00	.
141	C	CBB	.	HX2	A	3	-19.315	-2.955	-5.403	1.00	.
142	S	S2	.	HX2	A	3	-20.641	-2.944	-2.986	1.00	.
143	C	C2C	.	HX2	A	3	-18.162	-0.709	-0.560	1.00	.
144	C	CMC	.	HX2	A	3	-19.488	-0.615	0.136	1.00	.
145	H	H1	.	HX2	A	3	-16.008	2.399	-1.677	1.00	.
146	H	H21	.	HX2	A	3	-17.491	2.772	-2.022	1.00	.
147	H	H31	.	HX2	A	3	-16.717	3.575	-0.920	1.00	.
148	H	H4	.	HX2	A	3	-18.289	1.931	-0.070	1.00	.
149	H	H6	.	HX2	A	3	-15.867	-2.101	-2.126	1.00	.
150	H	H7	.	HX2	A	3	-18.857	-3.373	-0.605	1.00	.
151	H	H8	.	HX2	A	3	-14.408	-5.128	-3.940	1.00	.
152	H	H9	.	HX2	A	3	-12.252	-3.979	-2.617	1.00	.
153	H	H10	.	HX2	A	3	-10.573	-2.594	1.228	1.00	.
154	H	H11	.	HX2	A	3	-11.917	-3.544	2.904	1.00	.
155	H	H13	.	HX2	A	3	-13.172	-6.651	0.692	1.00	.
156	H	H14	.	HX2	A	3	-11.784	-5.906	0.800	1.00	.
157	H	H15	.	HX2	A	3	-12.114	-7.536	-1.039	1.00	.
158	H	H16	.	HX2	A	3	-12.887	-6.352	-1.759	1.00	.
159	H	H17	.	HX2	A	3	-16.486	-5.030	-0.975	1.00	.
160	H	H18	.	HX2	A	3	-16.018	-5.061	0.594	1.00	.
161	H	H19	.	HX2	A	3	-15.087	1.218	-2.377	1.00	.
162	H	H20	.	HX2	A	3	-12.705	2.842	0.406	1.00	.
163	H	H211	.	HX2	A	3	-14.104	2.710	-0.320	1.00	.
164	H	H22	.	HX2	A	3	-13.922	2.161	1.152	1.00	.
165	H	H23	.	HX2	A	3	-10.603	1.289	0.968	1.00	.
166	H	H24	.	HX2	A	3	-10.417	-0.242	1.305	1.00	.
167	H	H25	.	HX2	A	3	-11.796	-0.143	3.083	1.00	.
168	H	H26	.	HX2	A	3	-11.100	1.273	3.155	1.00	.
169	H	H27	.	HX2	A	3	-17.757	-5.666	-5.903	1.00	.
170	H	H28	.	HX2	A	3	-16.187	-5.651	-5.711	1.00	.
171	H	H29	.	HX2	A	3	-16.947	-4.325	-6.121	1.00	.
172	H	H30	.	HX2	A	3	-20.025	-4.618	-4.450	1.00	.
173	H	H311	.	HX2	A	3	-18.739	-3.441	-6.019	1.00	.
174	H	H32	.	HX2	A	3	-18.887	-2.118	-5.150	1.00	.
175	H	H33	.	HX2	A	3	-20.165	-2.766	-5.838	1.00	.
176	H	H34	.	HX2	A	3	-20.741	-1.875	-3.550	1.00	.
177	H	H35	.	HX2	A	3	-19.486	0.143	0.741	1.00	.
178	H	H36	.	HX2	A	3	-20.193	-0.500	-0.522	1.00	.
179	H	H37	.	HX2	A	3	-19.649	-1.430	0.639	1.00	.
180	N	N	.	HX2	A	3	-12.938	-5.131	4.811	1.00	.
181	C	CA	.	HX2	A	3	-14.316	-4.576	4.781	1.00	.
182	C	C	.	HX2	A	3	-15.301	-5.680	4.361	1.00	.
183	O	O	.	HX2	A	3	-14.956	-6.863	4.572	1.00	.
184	C	CB	.	HX2	A	3	-14.390	-3.396	3.815	1.00	.
185	C	CG	.	HX2	A	3	-14.628	-3.818	2.372	1.00	.
186	O	OXT	.	HX2	A	3	-16.377	-5.316	3.838	1.00	.
187	H	H	.	HX2	A	3	-12.693	-5.391	3.982	1.00	.
188	H	H2	.	HX2	A	3	-12.905	-5.844	5.363	1.00	.
189	H	H3	.	HX2	A	3	-12.359	-4.506	5.104	1.00	.
190	H	HA	.	HX2	A	3	-14.550	-4.268	5.694	1.00	.
191	H	HB3	.	HX2	A	3	-13.550	-2.893	3.865	1.00	.
192	H	HB2	.	HX2	A	3	-15.117	-2.801	4.097	1.00	.
193	H	HG3	.	HX2	A	3	-15.206	-3.148	1.936	1.00	.
194	H	HG2	.	HX2	A	3	-15.111	-4.679	2.369	1.00	.
195	N	N	.	CYS	A	5	-19.811	3.740	2.578	1.00	.
196	C	CA	.	CYS	A	5	-18.962	2.811	1.785	1.00	.
197	C	C	.	CYS	A	5	-19.729	1.504	1.526	1.00	.
198	O	O	.	CYS	A	5	-19.804	0.689	2.472	1.00	.
199	C	CB	.	CYS	A	5	-17.613	2.541	2.453	1.00	.
200	S	SG	.	CYS	A	5	-16.397	2.024	1.264	1.00	.

201	O	OXT	.	CYS	A	5	-20.223	1.349	0.387	1.00	.
202	H	H	.	CYS	A	5	-20.270	3.277	3.200	1.00	.
203	H	H2	.	CYS	A	5	-19.292	4.352	2.988	1.00	.
204	H	H3	.	CYS	A	5	-20.393	4.162	2.032	1.00	.
205	H	HA	.	CYS	A	5	-18.768	3.241	0.914	1.00	.
206	H	HB3	.	CYS	A	5	-17.316	3.364	2.895	1.00	.
207	H	HB2	.	CYS	A	5	-17.737	1.840	3.127	1.00	.
208	H	HG	.	CYS	A	5	-15.567	2.908	1.090	1.00	.
209	H	HSG1	.	CYS	A	5	-15.851	0.991	1.634	1.00	.
210	N	N	.	CYS	A	9	7.013	1.935	-2.367	1.00	.
211	C	CA	.	CYS	A	9	7.311	1.474	-0.985	1.00	.
212	C	C	.	CYS	A	9	6.245	2.016	-0.016	1.00	.
213	O	O	.	CYS	A	9	5.639	1.183	0.694	1.00	.
214	C	CB	.	CYS	A	9	8.720	1.856	-0.529	1.00	.
215	S	SG	.	CYS	A	9	9.210	0.966	0.930	1.00	.
216	O	OXT	.	CYS	A	9	6.060	3.254	-0.009	1.00	.
217	H	H	.	CYS	A	9	7.009	2.837	-2.394	1.00	.
218	H	H2	.	CYS	A	9	6.206	1.627	-2.625	1.00	.
219	H	H3	.	CYS	A	9	7.642	1.628	-2.935	1.00	.
220	H	HA	.	CYS	A	9	7.267	0.484	-0.977	1.00	.
221	H	HB3	.	CYS	A	9	8.733	2.818	-0.343	1.00	.
222	H	HB2	.	CYS	A	9	9.342	1.670	-1.264	1.00	.
223	H	HG	.	CYS	A	9	8.599	1.428	1.887	1.00	.
224	H	HSG1	.	CYS	A	9	8.951	-0.223	0.844	1.00	.
225	C	CBC	.	HX2	A	11	5.922	-3.937	-0.076	1.00	.
226	C	CAC	.	HX2	A	11	6.886	-4.073	-1.237	1.00	.
227	S	S1	.	HX2	A	11	7.220	-5.811	-1.652	1.00	.
228	C	C3C	.	HX2	A	11	8.168	-3.316	-1.017	1.00	.
229	C	C4C	.	HX2	A	11	9.150	-3.607	-0.002	1.00	.
230	N	NC	.	HX2	A	11	10.160	-2.690	-0.147	1.00	.
231	C	C1C	.	HX2	A	11	9.890	-1.824	-1.176	1.00	.
232	C	CHC	.	HX2	A	11	10.719	-0.773	-1.562	1.00	.
233	C	C4B	.	HX2	A	11	11.795	-0.249	-0.886	1.00	.
234	N	NB	.	HX2	A	11	13.063	-0.174	-1.378	1.00	.
235	C	C1B	.	HX2	A	11	13.766	-0.715	-0.336	1.00	.
236	C	CHB	.	HX2	A	11	14.889	-1.512	-0.482	1.00	.
237	C	C4A	.	HX2	A	11	15.119	-2.757	0.089	1.00	.
238	N	NA	.	HX2	A	11	14.243	-3.521	0.864	1.00	.
239	C	C1A	.	HX2	A	11	14.401	-4.822	0.508	1.00	.
240	C	CHA	.	HX2	A	11	13.490	-5.891	0.432	1.00	.
241	N	NZ	.	HX2	A	11	13.832	-6.860	-0.454	1.00	.
242	C	CE	.	HX2	A	11	14.773	-6.666	-1.423	1.00	.
243	C	CD	.	HX2	A	11	15.829	-5.780	-1.231	1.00	.
244	C	C2A	.	HX2	A	11	15.863	-4.990	0.075	1.00	.
245	C	CAA	.	HX2	A	11	16.709	-5.774	1.113	1.00	.
246	C	CBA	.	HX2	A	11	16.856	-5.208	2.533	1.00	.
247	C	CGA	.	HX2	A	11	18.221	-4.622	2.855	1.00	.
248	O	O2A	.	HX2	A	11	18.320	-3.385	2.997	1.00	.
249	O	O1A	.	HX2	A	11	19.186	-5.409	2.962	1.00	.
250	C	C3A	.	HX2	A	11	16.277	-3.514	-0.004	1.00	.
251	C	CMA	.	HX2	A	11	17.513	-3.054	-0.099	1.00	.
252	C	C4D	.	HX2	A	11	12.247	-5.935	1.209	1.00	.
253	N	ND	.	HX2	A	11	11.522	-4.815	1.510	1.00	.
254	C	C1D	.	HX2	A	11	10.229	-5.266	1.468	1.00	.
255	C	CHD	.	HX2	A	11	9.135	-4.629	0.943	1.00	.
256	C	C2D	.	HX2	A	11	10.251	-6.626	2.009	1.00	.
257	C	CMD	.	HX2	A	11	9.122	-7.340	2.695	1.00	.
258	C	C3D	.	HX2	A	11	11.510	-7.080	1.755	1.00	.
259	C	CAD	.	HX2	A	11	12.033	-8.464	1.976	1.00	.
260	C	CBD	.	HX2	A	11	11.396	-9.500	1.066	1.00	.
261	C	CGD	.	HX2	A	11	11.834	-10.913	1.388	1.00	.

```
262 O  O1D  . HX2 A   11    11.312   -11.478     2.372  1.00 .
263 O  O2D  . HX2 A   11    12.696   -11.445     0.655  1.00 .
264 C  C2B  . HX2 A   11    13.095    -0.320     0.897  1.00 .
265 C  CMB  . HX2 A   11    13.635    -0.513     2.286  1.00 .
266 C  C3B  . HX2 A   11    11.920     0.235     0.478  1.00 .
267 C  CAB  . HX2 A   11    11.000     1.250     1.119  1.00 .
268 C  CBB  . HX2 A   11    11.198     1.387     2.631  1.00 .
269 C  C2C  . HX2 A   11     8.623    -2.218    -1.730  1.00 .
270 C  CMC  . HX2 A   11     7.933    -1.548    -2.885  1.00 .
271 H  H1   . HX2 A   11     6.250    -3.286     0.549  1.00 .
272 H  H21  . HX2 A   11     5.832    -4.785     0.367  1.00 .
273 H  H31  . HX2 A   11     5.064    -3.658    -0.405  1.00 .
274 H  H4   . HX2 A   11     6.440    -3.693    -2.032  1.00 .
275 H  H5   . HX2 A   11     8.299    -5.678    -2.188  1.00 .
276 H  H6   . HX2 A   11    10.879    -2.662     0.358  1.00 .
277 H  H7   . HX2 A   11    10.536    -0.391    -2.416  1.00 .
278 H  H8   . HX2 A   11    15.568    -1.169    -1.060  1.00 .
279 H  H9   . HX2 A   11    13.682    -3.232     1.473  1.00 .
280 H  H10  . HX2 A   11    13.426    -7.650    -0.403  1.00 .
281 H  H11  . HX2 A   11    14.697    -7.128    -2.239  1.00 .
282 H  H13  . HX2 A   11    17.608    -5.890     0.738  1.00 .
283 H  H14  . HX2 A   11    16.325    -6.672     1.191  1.00 .
284 H  H15  . HX2 A   11    16.673    -5.929     3.173  1.00 .
285 H  H16  . HX2 A   11    16.179    -4.514     2.678  1.00 .
286 H  H17  . HX2 A   11    18.242    -3.657    -0.111  1.00 .
287 H  H18  . HX2 A   11    17.668    -2.123    -0.153  1.00 .
288 H  H19  . HX2 A   11     8.281    -4.933     1.235  1.00 .
289 H  H20  . HX2 A   11     9.453    -8.147     3.116  1.00 .
290 H  H211 . HX2 A   11     8.442    -7.573     2.045  1.00 .
291 H  H22  . HX2 A   11     8.735    -6.762     3.370  1.00 .
292 H  H23  . HX2 A   11    11.873    -8.720     2.906  1.00 .
293 H  H24  . HX2 A   11    13.001    -8.466     1.829  1.00 .
294 H  H25  . HX2 A   11    10.419    -9.455     1.140  1.00 .
295 H  H26  . HX2 A   11    11.625    -9.309     0.131  1.00 .
296 H  H27  . HX2 A   11    13.904     0.341     2.654  1.00 .
297 H  H28  . HX2 A   11    14.405    -1.099     2.264  1.00 .
298 H  H29  . HX2 A   11    12.954    -0.907     2.850  1.00 .
299 H  H30  . HX2 A   11    11.197     2.135     0.714  1.00 .
300 H  H311 . HX2 A   11    10.530     1.995     2.993  1.00 .
301 H  H32  . HX2 A   11    11.104     0.517     3.053  1.00 .
302 H  H33  . HX2 A   11    12.084     1.746     2.812  1.00 .
303 H  H35  . HX2 A   11     6.980    -1.490    -2.704  1.00 .
304 H  H36  . HX2 A   11     8.076    -2.064    -3.694  1.00 .
305 H  H37  . HX2 A   11     8.288    -0.654    -3.007  1.00 .
306 N  N    . HX2 A   11    19.366    -8.396    -2.799  1.00 .
307 C  CA   . HX2 A   11    18.228    -7.785    -2.058  1.00 .
308 C  C    . HX2 A   11    17.347    -8.905    -1.473  1.00 .
309 O  O    . HX2 A   11    16.954    -9.795    -2.260  1.00 .
310 C  CB   . HX2 A   11    17.437    -6.852    -2.979  1.00 .
311 C  CG   . HX2 A   11    16.898    -5.597    -2.290  1.00 .
312 O  OXT  . HX2 A   11    17.085    -8.847    -0.251  1.00 .
313 H  H    . HX2 A   11    19.061    -8.821    -3.533  1.00 .
314 H  H2   . HX2 A   11    19.801    -8.988    -2.276  1.00 .
315 H  H3   . HX2 A   11    19.949    -7.756    -3.052  1.00 .
316 H  HA   . HX2 A   11    18.600    -7.255    -1.308  1.00 .
317 H  HB3  . HX2 A   11    18.020    -6.573    -3.716  1.00 .
318 H  HB2  . HX2 A   11    16.691    -7.348    -3.377  1.00 .
319 H  HG3  . HX2 A   11    16.545    -4.990    -2.984  1.00 .
320 H  HG2  . HX2 A   11    17.666    -5.127    -1.888  1.00 .

data_comp_list
```

```
loop_
_chem_comp.id
_chem_comp.three_letter_code
_chem_comp.name
_chem_comp.group
_chem_comp.number_atoms_all
_chem_comp.number_atoms_nh
_chem_comp.desc_level
HX2      HX2 .                                   peptide            98  54.

data_mod_list

loop_
_chem_mod.id
_chem_mod.name
_chem_mod.comp_id
_chem_mod.group_id
HX2mod1  .                                                HX2      .
CYSmod1  .                                                CYS      .
CYSmod2  .                                                CYS      .
HX2mod2  .                                                HX2      .

data_link_list

loop_
_chem_link.id
_chem_link.comp_id_1
_chem_link.mod_id_1
_chem_link.group_comp_1
_chem_link.comp_id_2
_chem_link.mod_id_2
_chem_link.group_comp_2
_chem_link.name
HX2-CYS  HX2      HX2mod1  .          CYS      CYSmod1.
.
CYS-HX2  CYS      CYSmod2  .          HX2      HX2mod2.
.

data_comp_HX2

loop_
_chem_comp_atom.comp_id
_chem_comp_atom.atom_id
_chem_comp_atom.type_symbol
_chem_comp_atom.type_energy
_chem_comp_atom.charge
_chem_comp_atom.x
_chem_comp_atom.y
_chem_comp_atom.z
HX2           CBC    C    CH3       0      -6.641   -7.573    0.719
HX2           CAC    C    CH1       0      -7.081   -8.936    0.230
HX2           S1     S    SH1       0      -8.068   -9.810    1.479
HX2           C3C    C    CR5       0      -5.909   -9.768   -0.200
HX2           C4C    C    CR5       0      -5.630  -11.153    0.091
HX2           NC     N    NR15      0      -4.437  -11.454   -0.515
HX2           C1C    C    CR5       0      -3.950  -10.365   -1.191
HX2           CHC    C    C1        0      -2.748  -10.327   -1.875
HX2           C4B    C    CR5       0      -1.533  -10.269   -1.260
HX2           NB     N    NRD5      0      -0.458  -11.076   -1.501
```

HX2	C1B	C	CR5	0	0.117	-11.139	-0.261
HX2	CHB	C	C1	0	0.649	-12.275	0.323
HX2	C4A	C	CR5	0	-0.030	-13.482	0.375
HX2	NA	N	NR15	0	-1.298	-13.719	0.914
HX2	C1A	C	CR56	0	-1.933	-14.576	0.071
HX2	CHA	C	CR6	0	-3.236	-14.657	-0.431
HX2	NZ	N	NR16	0	-3.262	-14.679	-1.782
HX2	CE	C	CR16	0	-2.139	-14.930	-2.493
HX2	CD	C	CR6	0	-1.145	-15.674	-1.924
HX2	C2A	C	CT	0	-0.855	-15.513	-0.446
HX2	CAA	C	CH2	0	-0.808	-16.870	0.292
HX2	CBA	C	CH2	0	0.572	-17.295	0.774
HX2	CGA	C	C	0	1.609	-17.386	-0.331
HX2	O2A	O	O	0	2.377	-18.370	-0.338
HX2	O1A	O	OC	-1	1.648	-16.472	-1.181
HX2	C3A	C	CR5	0	0.398	-14.697	-0.132
HX2	CMA	C	C2	0	1.641	-15.100	-0.319
HX2	C4D	C	CR5	0	-4.485	-14.675	0.334
HX2	ND	N	NRD5	0	-5.643	-14.166	-0.175
HX2	C1D	C	CR5	0	-6.043	-13.391	0.876
HX2	CHD	C	C1	0	-6.366	-12.067	0.831
HX2	C2D	C	CR5	0	-5.823	-14.207	2.072
HX2	CMD	C	CH3	0	-6.525	-14.092	3.394
HX2	C3D	C	CR5	0	-4.846	-15.078	1.697
HX2	CAD	C	CH2	0	-4.292	-16.224	2.484
HX2	CBD	C	CH2	0	-4.746	-17.656	2.253
HX2	CGD	C	C	0	-3.670	-18.676	2.566
HX2	O1D	O	O	0	-3.193	-18.691	3.720
HX2	O2D	O	OC	-1	-3.314	-19.452	1.654
HX2	C2B	C	CR5	0	-0.044	-9.818	0.324
HX2	CMB	C	CH3	0	0.900	-9.109	1.250
HX2	C3B	C	CR5	0	-1.225	-9.379	-0.160
HX2	CAB	C	CH1	0	-2.097	-8.254	0.325
HX2	CBB	C	CH3	0	-1.883	-7.984	1.797
HX2	S2	S	SH1	0	-3.854	-8.546	-0.033
HX2	C2C	C	CR5	0	-4.874	-9.290	-0.977
HX2	CMC	C	CH3	0	-4.720	-7.900	-1.520
HX2	H1	H	H	0	-5.862	-7.294	0.231
HX2	H21	H	H	0	-7.349	-6.940	0.584
HX2	H31	H	H	0	-6.430	-7.623	1.655
HX2	H4	H	H	0	-7.662	-8.800	-0.561
HX2	H5	H	HSH1	0	-7.279	-9.790	2.399
HX2	H6	H	H	0	-4.048	-12.241	-0.490
HX2	H7	H	H	0	-2.764	-10.326	-2.827
HX2	H8	H	H	0	1.524	-12.219	0.701
HX2	H9	H	H	0	-1.633	-13.385	1.654
HX2	H10	H	H	0	-4.029	-14.526	-2.207
HX2	H11	H	H	0	-2.033	-14.601	-3.369
HX2	H13	H	H	0	-1.158	-17.563	-0.307
HX2	H14	H	H	0	-1.409	-16.826	1.065
HX2	H15	H	H	0	0.883	-16.651	1.445
HX2	H16	H	H	0	0.497	-18.172	1.207
HX2	H17	H	H	0	2.362	-14.530	-0.095
HX2	H18	H	H	0	1.810	-15.958	-0.676
HX2	H19	H	H	0	-7.112	-11.768	1.345
HX2	H20	H	H	0	-7.472	-13.941	3.247
HX2	H211	H	H	0	-6.405	-14.911	3.899
HX2	H22	H	H	0	-6.156	-13.350	3.896
HX2	H23	H	H	0	-3.999	-16.415	1.571
HX2	H24	H	H	0	-5.235	-16.211	2.740
HX2	H25	H	H	0	-5.529	-17.853	2.811

```
HX2           H26    H    H         0     -5.020  -17.773    1.319
HX2           H27    H    H         0      0.420   -8.425    1.741
HX2           H28    H    H         0      1.284   -9.744    1.873
HX2           H29    H    H         0      1.610   -8.695    0.735
HX2           H30    H    H         0     -1.829   -7.443   -0.175
HX2           H311   H    H         0     -1.654   -7.170    2.278
HX2           H32    H    H         0     -2.699   -7.474    1.655
HX2           H33    H    H         0     -0.975   -7.853    1.474
HX2           H34    H    HSH1      0     -4.093   -9.342    0.852
HX2           H35    H    H         0     -5.512   -7.379   -1.313
HX2           H36    H    H         0     -3.943   -7.478   -1.118
HX2           H37    H    H         0     -4.602   -7.938   -2.483
HX2           N      N    NT3       1     -2.370  -19.884   -2.666
HX2           CA     C    CH1       0     -1.083  -19.140   -2.702
HX2           C      C    C         0     -0.528  -19.017   -1.274
HX2           O      O    O         0      0.650  -19.394   -1.084
HX2           CB     C    CH2       0     -1.283  -17.760   -3.329
HX2           CG     C    CH2       0     -0.379  -16.685   -2.741
HX2           OXT    O    OC       -1     -1.293  -18.549   -0.402
HX2           H      H    H         0     -2.463  -20.297   -1.869
HX2           H2     H    H         0     -2.386  -20.503   -3.321
HX2           H3     H    H         0     -3.060  -19.313   -2.783
HX2           HA     H    H         0     -0.440  -19.656   -3.251
HX2           HB3    H    H         0     -2.217  -17.491   -3.201
HX2           HB2    H    H         0     -1.116  -17.825   -4.292
HX2           HG3    H    H         0      0.300  -17.114   -2.168
HX2           HG2    H    H         0      0.085  -16.218   -3.477

loop_
_chem_comp_bond.comp_id
_chem_comp_bond.atom_id_1
_chem_comp_bond.atom_id_2
_chem_comp_bond.type
_chem_comp_bond.aromatic
_chem_comp_bond.value_dist
_chem_comp_bond.value_dist_esd
HX2      N      CA        single   n    1.487    0.010
HX2      CA     C         single   n    1.538    0.011
HX2      C      O         double   n    1.251    0.018
HX2      C      OXT       single   n    1.251    0.018
HX2      CA     CB        single   n    1.529    0.010
HX2      CB     CG        single   n    1.523    0.019
HX2      CD     CG        single   n    1.511    0.010
HX2      CBC    CAC       single   n    1.513    0.012
HX2      CAC    C3C       single   n    1.503    0.010
HX2      CAC    S1        single   n    1.817    0.016
HX2      C3C    C4C       double   y    1.442    0.017
HX2      C3C    C2C       single   y    1.375    0.020
HX2      C4C    NC        single   y    1.373    0.010
HX2      NC     C1C       single   y    1.373    0.010
HX2      C1C    CHC       single   n    1.392    0.019
HX2      CHC    C4B       double   n    1.372    0.020
HX2      C4B    C3B       single   n    1.451    0.017
HX2      C4B    NB        single   n    1.365    0.010
HX2      NB     C1B       double   n    1.367    0.012
HX2      C1B    C2B       single   n    1.453    0.010
HX2      C1B    CHB       single   n    1.384    0.011
HX2      CHB    C4A       double   n    1.382    0.020
HX2      C4A    NA        single   n    1.396    0.010
HX2      NA     C1A       single   n    1.363    0.020
HX2      C1A    CHA       double   n    1.396    0.020
```

HX2	CHA	NZ	single	n	1.351	0.013
HX2	NZ	CE	single	n	1.354	0.016
HX2	CE	CD	double	n	1.368	0.020
HX2	C1A	C2A	single	n	1.525	0.017
HX2	CD	C2A	single	n	1.515	0.013
HX2	C2A	C3A	single	n	1.525	0.017
HX2	C2A	CAA	single	n	1.547	0.014
HX2	CAA	CBA	single	n	1.521	0.019
HX2	CBA	CGA	single	n	1.518	0.014
HX2	CGA	O2A	double	n	1.249	0.016
HX2	CGA	O1A	single	n	1.249	0.016
HX2	C4A	C3A	single	n	1.381	0.020
HX2	C3A	CMA	double	n	1.321	0.010
HX2	CHA	C4D	single	n	1.464	0.015
HX2	C4D	ND	double	n	1.366	0.020
HX2	ND	C1D	single	n	1.374	0.016
HX2	C4C	CHD	single	n	1.392	0.019
HX2	C1D	CHD	double	n	1.372	0.020
HX2	C1D	C2D	single	n	1.467	0.010
HX2	C2D	CMD	single	n	1.501	0.010
HX2	C4D	C3D	single	n	1.458	0.018
HX2	C2D	C3D	double	n	1.360	0.011
HX2	C3D	CAD	single	n	1.497	0.010
HX2	CAD	CBD	single	n	1.519	0.020
HX2	CBD	CGD	single	n	1.515	0.012
HX2	CGD	O2D	single	n	1.249	0.016
HX2	CGD	O1D	double	n	1.249	0.016
HX2	C2B	CMB	single	n	1.501	0.010
HX2	C2B	C3B	double	n	1.354	0.013
HX2	C3B	CAB	single	n	1.505	0.014
HX2	CAB	CBB	single	n	1.513	0.012
HX2	CAB	S2	single	n	1.817	0.016
HX2	C1C	C2C	double	y	1.434	0.010
HX2	C2C	CMC	single	n	1.501	0.011
HX2	CBC	H1	single	n	0.960	0.010
HX2	CBC	H21	single	n	0.960	0.010
HX2	CBC	H31	single	n	0.960	0.010
HX2	CAC	H4	single	n	0.990	0.020
HX2	S1	H5	single	n	1.212	0.020
HX2	NC	H6	single	n	0.879	0.020
HX2	CHC	H7	single	n	0.953	0.019
HX2	CHB	H8	single	n	0.956	0.020
HX2	NA	H9	single	n	0.878	0.020
HX2	NZ	H10	single	n	0.890	0.020
HX2	CE	H11	single	n	0.942	0.018
HX2	CAA	H13	single	n	0.981	0.016
HX2	CAA	H14	single	n	0.981	0.016
HX2	CBA	H15	single	n	0.981	0.017
HX2	CBA	H16	single	n	0.981	0.017
HX2	CMA	H18	single	n	0.945	0.020
HX2	CMA	H17	single	n	0.945	0.020
HX2	CHD	H19	single	n	0.953	0.019
HX2	CMD	H20	single	n	0.969	0.015
HX2	CMD	H211	single	n	0.969	0.015
HX2	CMD	H22	single	n	0.969	0.015
HX2	CAD	H23	single	n	0.978	0.017
HX2	CAD	H24	single	n	0.978	0.017
HX2	CBD	H25	single	n	0.981	0.011
HX2	CBD	H26	single	n	0.981	0.011
HX2	CMB	H27	single	n	0.969	0.015
HX2	CMB	H28	single	n	0.969	0.015

```
HX2      CMB    H29      single   n    0.969    0.015
HX2      CAB    H30      single   n    0.990    0.020
HX2      CBB    H311     single   n    0.973    0.010
HX2      CBB    H33      single   n    0.973    0.010
HX2      CBB    H32      single   n    0.973    0.010
HX2      S2     H34      single   n    1.212    0.020
HX2      CMC    H35      single   n    0.971    0.014
HX2      CMC    H36      single   n    0.971    0.014
HX2      CMC    H37      single   n    0.971    0.014
HX2      N      H        single   n    0.902    0.010
HX2      N      H2       single   n    0.902    0.010
HX2      N      H3       single   n    0.902    0.010
HX2      CA     HA       single   n    0.991    0.020
HX2      CB     HB2      single   n    0.980    0.017
HX2      CB     HB3      single   n    0.980    0.017
HX2      CG     HG2      single   n    0.987    0.010
HX2      CG     HG3      single   n    0.987    0.010

loop_
_chem_comp_angle.comp_id
_chem_comp_angle.atom_id_1
_chem_comp_angle.atom_id_2
_chem_comp_angle.atom_id_3
_chem_comp_angle.value_angle
_chem_comp_angle.value_angle_esd
HX2      CAC    CBC    H1      109.518    1.500
HX2      CAC    CBC    H21     109.518    1.500
HX2      CAC    CBC    H31     109.518    1.500
HX2      H1     CBC    H21     109.460    1.500
HX2      H1     CBC    H31     109.460    1.500
HX2      H21    CBC    H31     109.460    1.500
HX2      CBC    CAC    C3C     112.400    1.500
HX2      CBC    CAC    S1      112.610    3.000
HX2      CBC    CAC    H4      108.549    2.040
HX2      S1     CAC    C3C     111.652    3.000
HX2      C3C    CAC    H4      108.177    1.500
HX2      S1     CAC    H4      108.757    3.000
HX2      CAC    S1     H5       99.186    3.000
HX2      CAC    C3C    C4C     126.768    3.000
HX2      CAC    C3C    C2C     125.511    3.000
HX2      C4C    C3C    C2C     107.721    1.500
HX2      C3C    C4C    NC      107.299    1.500
HX2      C3C    C4C    CHD     127.218    1.940
HX2      NC     C4C    CHD     125.483    1.570
HX2      C4C    NC     C1C     110.518    1.500
HX2      C4C    NC     H6      124.741    3.000
HX2      C1C    NC     H6      124.741    3.000
HX2      NC     C1C    CHC     125.600    1.570
HX2      NC     C1C    C2C     106.742    1.500
HX2      CHC    C1C    C2C     127.658    3.000
HX2      C1C    CHC    C4B     128.527    3.000
HX2      C1C    CHC    H7      115.410    2.170
HX2      C4B    CHC    H7      116.063    1.500
HX2      CHC    C4B    C3B     125.026    2.880
HX2      CHC    C4B    NB      125.218    1.500
HX2      NB     C4B    C3B     109.756    2.280
HX2      C4B    NB     C1B     106.462    2.670
HX2      NB     C1B    C2B     110.179    3.000
HX2      NB     C1B    CHB     125.177    1.680
HX2      CHB    C1B    C2B     124.644    2.930
HX2      C1B    CHB    C4A     125.148    3.000
```

HX2	C1B	CHB	H8	116.958	3.000
HX2	C4A	CHB	H8	117.894	3.000
HX2	CHB	C4A	NA	126.984	3.000
HX2	CHB	C4A	C3A	126.493	3.000
HX2	NA	C4A	C3A	106.523	3.000
HX2	C4A	NA	C1A	109.510	3.000
HX2	C4A	NA	H9	126.008	3.000
HX2	C1A	NA	H9	124.482	3.000
HX2	NA	C1A	CHA	130.085	3.000
HX2	NA	C1A	C2A	107.838	3.000
HX2	CHA	C1A	C2A	122.076	3.000
HX2	C1A	CHA	NZ	116.163	3.000
HX2	C1A	CHA	C4D	122.143	3.000
HX2	NZ	CHA	C4D	121.694	3.000
HX2	CHA	NZ	CE	122.624	2.880
HX2	CHA	NZ	H10	118.481	3.000
HX2	CE	NZ	H10	118.895	3.000
HX2	NZ	CE	CD	120.688	3.000
HX2	NZ	CE	H11	119.999	2.540
HX2	CD	CE	H11	119.312	1.500
HX2	CE	CD	CG	120.858	3.000
HX2	C2A	CD	CG	117.577	3.000
HX2	CE	CD	C2A	121.565	3.000
HX2	C1A	C2A	CD	109.895	3.000
HX2	C1A	C2A	C3A	101.524	2.900
HX2	C1A	C2A	CAA	111.194	3.000
HX2	CD	C2A	C3A	109.856	3.000
HX2	CD	C2A	CAA	109.486	3.000
HX2	CAA	C2A	C3A	108.076	3.000
HX2	C2A	CAA	CBA	114.329	3.000
HX2	C2A	CAA	H13	108.328	1.500
HX2	C2A	CAA	H14	108.328	1.500
HX2	CBA	CAA	H13	108.359	1.500
HX2	CBA	CAA	H14	108.359	1.500
HX2	H13	CAA	H14	106.929	1.500
HX2	CAA	CBA	CGA	113.560	3.000
HX2	CAA	CBA	H15	108.638	1.500
HX2	CAA	CBA	H16	108.638	1.500
HX2	CGA	CBA	H15	108.531	1.500
HX2	CGA	CBA	H16	108.531	1.500
HX2	H15	CBA	H16	107.705	2.230
HX2	CBA	CGA	O2A	118.194	3.000
HX2	CBA	CGA	O1A	118.194	3.000
HX2	O2A	CGA	O1A	123.612	1.820
HX2	C4A	C3A	C2A	108.027	3.000
HX2	C2A	C3A	CMA	124.704	3.000
HX2	C4A	C3A	CMA	127.269	3.000
HX2	C3A	CMA	H18	119.932	1.500
HX2	C3A	CMA	H17	119.932	1.500
HX2	H17	CMA	H18	120.136	1.500
HX2	CHA	C4D	ND	123.012	3.000
HX2	CHA	C4D	C3D	127.850	3.000
HX2	ND	C4D	C3D	109.138	3.000
HX2	C4D	ND	C1D	107.166	2.340
HX2	ND	C1D	CHD	125.680	3.000
HX2	ND	C1D	C2D	109.717	2.280
HX2	CHD	C1D	C2D	124.603	1.500
HX2	C4C	CHD	C1D	128.527	3.000
HX2	C4C	CHD	H19	115.410	2.170
HX2	C1D	CHD	H19	116.063	1.500
HX2	C1D	C2D	CMD	126.711	1.500

HX2	C1D	C2D	C3D	106.953	1.500
HX2	CMD	C2D	C3D	126.336	3.000
HX2	C2D	CMD	H20	109.573	1.500
HX2	C2D	CMD	H211	109.573	1.500
HX2	C2D	CMD	H22	109.573	1.500
HX2	H20	CMD	H211	109.306	2.100
HX2	H20	CMD	H22	109.306	2.100
HX2	H211	CMD	H22	109.306	2.100
HX2	C4D	C3D	C2D	107.026	1.500
HX2	C4D	C3D	CAD	126.632	3.000
HX2	C2D	C3D	CAD	126.342	3.000
HX2	C3D	CAD	CBD	113.552	1.680
HX2	C3D	CAD	H23	109.334	3.000
HX2	C3D	CAD	H24	109.334	3.000
HX2	CBD	CAD	H23	109.251	3.000
HX2	CBD	CAD	H24	109.251	3.000
HX2	H23	CAD	H24	107.902	2.140
HX2	CAD	CBD	CGD	113.745	3.000
HX2	CAD	CBD	H25	111.034	3.000
HX2	CAD	CBD	H26	111.034	3.000
HX2	CGD	CBD	H25	108.600	1.500
HX2	CGD	CBD	H26	108.600	1.500
HX2	H25	CBD	H26	107.539	1.500
HX2	CBD	CGD	O2D	118.035	1.950
HX2	CBD	CGD	O1D	118.035	1.950
HX2	O1D	CGD	O2D	123.930	1.820
HX2	C1B	C2B	CMB	126.661	1.500
HX2	C1B	C2B	C3B	106.802	1.500
HX2	CMB	C2B	C3B	126.537	3.000
HX2	C2B	CMB	H27	109.573	1.500
HX2	C2B	CMB	H28	109.573	1.500
HX2	C2B	CMB	H29	109.573	1.500
HX2	H27	CMB	H28	109.306	2.100
HX2	H27	CMB	H29	109.306	2.100
HX2	H28	CMB	H29	109.306	2.100
HX2	C4B	C3B	C2B	106.802	1.500
HX2	C4B	C3B	CAB	126.599	3.000
HX2	C2B	C3B	CAB	126.599	3.000
HX2	C3B	CAB	CBB	111.467	2.280
HX2	C3B	CAB	S2	112.915	1.840
HX2	C3B	CAB	H30	108.198	2.620
HX2	CBB	CAB	S2	112.610	3.000
HX2	CBB	CAB	H30	108.549	2.040
HX2	S2	CAB	H30	108.757	3.000
HX2	CAB	CBB	H311	109.518	1.500
HX2	CAB	CBB	H33	109.518	1.500
HX2	CAB	CBB	H32	109.518	1.500
HX2	H311	CBB	H33	109.466	1.500
HX2	H311	CBB	H32	109.466	1.500
HX2	H32	CBB	H33	109.466	1.500
HX2	CAB	S2	H34	99.186	3.000
HX2	C3C	C2C	C1C	107.721	1.500
HX2	C3C	C2C	CMC	126.856	3.000
HX2	C1C	C2C	CMC	125.423	1.500
HX2	C2C	CMC	H35	109.572	1.500
HX2	C2C	CMC	H36	109.572	1.500
HX2	C2C	CMC	H37	109.572	1.500
HX2	H35	CMC	H36	109.322	1.870
HX2	H35	CMC	H37	109.322	1.870
HX2	H36	CMC	H37	109.322	1.870
HX2	CA	N	H	109.990	3.000

```
HX2       CA      N       H2       109.990    3.000
HX2       CA      N       H3       109.990    3.000
HX2       H       N       H2       109.032    3.000
HX2       H       N       H3       109.032    3.000
HX2       H2      N       H3       109.032    3.000
HX2       N       CA      C        109.258    1.500
HX2       N       CA      CB       110.314    2.210
HX2       N       CA      HA       108.387    1.580
HX2       C       CA      CB       110.876    3.000
HX2       C       CA      HA       108.774    1.790
HX2       CB      CA      HA       109.208    1.870
HX2       CA      C       O        117.148    1.600
HX2       CA      C       OXT      117.148    1.600
HX2       O       C       OXT      125.704    1.500
HX2       CA      CB      CG       113.420    2.400
HX2       CA      CB      HB2      108.559    1.500
HX2       CA      CB      HB3      108.559    1.500
HX2       CG      CB      HB2      108.800    1.500
HX2       CG      CB      HB3      108.800    1.500
HX2       HB3     CB      HB2      107.693    2.030
HX2       CD      CG      CB       113.967    3.000
HX2       CB      CG      HG2      108.780    1.500
HX2       CB      CG      HG3      108.780    1.500
HX2       CD      CG      HG2      108.753    1.500
HX2       CD      CG      HG3      108.753    1.500
HX2       HG3     CG      HG2      107.681    2.990

loop_
_chem_comp_tor.comp_id
_chem_comp_tor.id
_chem_comp_tor.atom_id_1
_chem_comp_tor.atom_id_2
_chem_comp_tor.atom_id_3
_chem_comp_tor.atom_id_4
_chem_comp_tor.value_angle
_chem_comp_tor.value_angle_esd
_chem_comp_tor.period
HX2       sp3_sp3_29 S1      CAC     CBC     H1        -60.000   10.000   3
HX2       sp2_sp2_81 C4A     CHB     C1B     C2B       180.000   20.000   2
HX2       sp2_sp2_84 NB      C1B     CHB     H8        180.000   20.000   2
HX2       const_45 NB      C1B     C2B     C3B         0.000    0.000   1
HX2       const_48 CHB     C1B     C2B     CMB         0.000    0.000   1
HX2       sp2_sp2_85 C1B     CHB     C4A     NA        180.000   20.000   2
HX2       sp2_sp2_88 C3A     C4A     CHB     H8        180.000   20.000   2
HX2       const_sp2_sp2_1 C1A     NA      C4A     C3A         0.000    0.000   1
HX2       const_sp2_sp2_4 CHB     C4A     NA      H9          0.000    0.000   1
HX2       const_89 NA      C4A     C3A     C2A         0.000    0.000   1
HX2       const_92 CHB     C4A     C3A     CMA         0.000    0.000   1
HX2       const_sp2_sp2_5 C4A     NA      C1A     C2A         0.000    0.000   1
HX2       const_sp2_sp2_8 CHA     C1A     NA      H9          0.000    0.000   1
HX2       const_sp2_sp2_9 NZ      CHA     C1A     C2A         0.000    0.000   1
HX2       const_12 NA      C1A     CHA     C4D         0.000    0.000   1
HX2       sp2_sp3_2 NA      C1A     C2A     CAA       120.000   10.000   6
HX2       const_13 C1A     CHA     NZ      CE          0.000    0.000   1
HX2       const_16 C4D     CHA     NZ      H10         0.000    0.000   1
HX2       sp2_sp2_97 C1A     CHA     C4D     C3D       180.000   20.000   2
HX2       sp2_sp2_100 NZ      CHA     C4D     ND        180.000   20.000   2
HX2       const_17 CHA     NZ      CE      CD          0.000    0.000   1
HX2       const_20 H10     NZ      CE      H11         0.000    0.000   1
HX2       const_21 NZ      CE      CD      C2A         0.000    0.000   1
HX2       const_24 H11     CE      CD      CG          0.000    0.000   1
```

```
HX2      sp2_sp3_17 CAA    C2A    CD     CG        -60.000   10.000   6
HX2      sp2_sp3_26 CE     CD     CG     CB        -90.000   10.000   6
HX2      sp3_sp3_40 C1A    C2A    CAA    CBA       180.000   10.000   3
HX2      sp2_sp3_12 CAA    C2A    C3A    CMA        60.000   10.000   6
HX2      sp3_sp3_37 CBC    CAC    S1     H5        180.000   10.000   3
HX2      sp2_sp3_32 CBC    CAC    C3C    C4C       -90.000   10.000   6
HX2      sp3_sp3_49 C2A    CAA    CBA    CGA       180.000   10.000   3
HX2      sp2_sp3_38 CAA    CBA    CGA    O2A       120.000   10.000   6
HX2      sp2_sp2_93 C2A    C3A    CMA    H18       180.000   20.000   2
HX2      sp2_sp2_96 C4A    C3A    CMA    H17       180.000   20.000   2
HX2      const_25 C1D    ND     C4D    C3D       0.000    0.000   1
HX2      const_109 ND     C4D    C3D    C2D       0.000    0.000   1
HX2      const_112 CHA    C4D    C3D    CAD       0.000    0.000   1
HX2      const_27 C4D    ND     C1D    C2D       0.000    0.000   1
HX2      sp2_sp2_105 C4C    CHD    C1D    C2D       180.000   20.000   2
HX2      sp2_sp2_108 ND     C1D    CHD    H19       180.000   20.000   2
HX2      const_29 ND     C1D    C2D    C3D       0.000    0.000   1
HX2      const_32 CHD    C1D    C2D    CMD       0.000    0.000   1
HX2      sp2_sp3_43 C1D    C2D    CMD    H20       150.000   10.000   6
HX2      const_33 C4D    C3D    C2D    C1D       0.000    0.000   1
HX2      const_36 CMD    C2D    C3D    CAD       0.000    0.000   1
HX2      sp2_sp3_50 C4D    C3D    CAD    CBD       -90.000   10.000   6
HX2      sp3_sp3_58 C3D    CAD    CBD    CGD       180.000   10.000   3
HX2      sp2_sp3_56 CAD    CBD    CGD    O2D       120.000   10.000   6
HX2      sp2_sp3_61 C1B    C2B    CMB    H27       150.000   10.000   6
HX2      const_41 C4B    C3B    C2B    C1B       0.000    0.000   1
HX2      const_44 CMB    C2B    C3B    CAB       0.000    0.000   1
HX2      const_51 NC     C4C    C3C    C2C       0.000    0.000   1
HX2      const_54 CAC    C3C    C4C    CHD       0.000    0.000   1
HX2      const_67 C4C    C3C    C2C    C1C       0.000    0.000   1
HX2      const_70 CAC    C3C    C2C    CMC       0.000    0.000   1
HX2      sp2_sp3_68 C4B    C3B    CAB    CBB       -90.000   10.000   6
HX2      sp3_sp3_70 S2     CAB    CBB    H311       60.000   10.000   3
HX2      sp3_sp3_76 CBB    CAB    S2     H34       180.000   10.000   3
HX2      sp2_sp3_73 C3C    C2C    CMC    H35       150.000   10.000   6
HX2      sp3_sp3_4 C      CA     N      H         60.000   10.000   3
HX2      sp2_sp3_19 N      CA     C      O          0.000   10.000   6
HX2      sp3_sp3_13 N      CA     CB     CG        60.000   10.000   3
HX2      sp2_sp2_101 C3C    C4C    CHD    C1D       180.000   20.000   2
HX2      sp2_sp2_104 NC     C4C    CHD    H19       180.000   20.000   2
HX2      const_55 C3C    C4C    NC     C1C       0.000    0.000   1
HX2      const_58 CHD    C4C    NC     H6        0.000    0.000   1
HX2      sp3_sp3_19 CD     CG     CB     CA        180.000   10.000   3
HX2      const_59 C4C    NC     C1C    C2C       0.000    0.000   1
HX2      const_62 CHC    C1C    NC     H6        0.000    0.000   1
HX2      const_63 C3C    C2C    C1C    NC        0.000    0.000   1
HX2      const_66 CHC    C1C    C2C    CMC       0.000    0.000   1
HX2      sp2_sp2_71 NC     C1C    CHC    C4B       180.000   20.000   2
HX2      sp2_sp2_74 C2C    C1C    CHC    H7        180.000   20.000   2
HX2      sp2_sp2_75 C1C    CHC    C4B    C3B       180.000   20.000   2
HX2      sp2_sp2_78 NB     C4B    CHC    H7        180.000   20.000   2
HX2      const_37 NB     C4B    C3B    C2B       0.000    0.000   1
HX2      const_40 CHC    C4B    C3B    CAB       0.000    0.000   1
HX2      const_79 C1B    NB     C4B    C3B       0.000    0.000   1
HX2      const_49 C4B    NB     C1B    C2B       0.000    0.000   1

loop_
_chem_comp_chir.comp_id
_chem_comp_chir.id
_chem_comp_chir.atom_id_centre
_chem_comp_chir.atom_id_1
```

```
_chem_comp_chir.atom_id_2
_chem_comp_chir.atom_id_3
_chem_comp_chir.volume_sign
HX2      chir_1   CA     N      C      CB        positiv
HX2      chir_1   CAC    CBC    S1     C3C       positiv
HX2      chir_2   C2A    C1A    C3A    CD        positiv
HX2      chir_3   CAB    C3B    CBB    S2        positiv

loop_
_chem_comp_plane_atom.comp_id
_chem_comp_plane_atom.plane_id
_chem_comp_plane_atom.atom_id
_chem_comp_plane_atom.dist_esd
HX2      plan-1    CAC       0.020
HX2      plan-1    C3C       0.020
HX2      plan-1    C4C       0.020
HX2      plan-1    NC        0.020
HX2      plan-1    C1C       0.020
HX2      plan-1    CHC       0.020
HX2      plan-1    CHD       0.020
HX2      plan-1    C2C       0.020
HX2      plan-1    CMC       0.020
HX2      plan-1    H6        0.020
HX2      plan-2    C1C       0.020
HX2      plan-2    CHC       0.020
HX2      plan-2    C4B       0.020
HX2      plan-2    H7        0.020
HX2      plan-3    CHC       0.020
HX2      plan-3    C4B       0.020
HX2      plan-3    NB        0.020
HX2      plan-3    C3B       0.020
HX2      plan-4    NB        0.020
HX2      plan-4    C1B       0.020
HX2      plan-4    CHB       0.020
HX2      plan-4    C2B       0.020
HX2      plan-5    C1B       0.020
HX2      plan-5    CHB       0.020
HX2      plan-5    C4A       0.020
HX2      plan-5    H8        0.020
HX2      plan-6    CHB       0.020
HX2      plan-6    C4A       0.020
HX2      plan-6    NA        0.020
HX2      plan-6    C3A       0.020
HX2      plan-7    C4A       0.020
HX2      plan-7    NA        0.020
HX2      plan-7    C1A       0.020
HX2      plan-7    H9        0.020
HX2      plan-8    NA        0.020
HX2      plan-8    C1A       0.020
HX2      plan-8    CHA       0.020
HX2      plan-8    C2A       0.020
HX2      plan-9    C1A       0.020
HX2      plan-9    CHA       0.020
HX2      plan-9    NZ        0.020
HX2      plan-9    C4D       0.020
HX2      plan-10   CHA       0.020
HX2      plan-10   NZ        0.020
HX2      plan-10   CE        0.020
HX2      plan-10   H10       0.020
HX2      plan-11   NZ        0.020
HX2      plan-11   CE        0.020
```

```
HX2      plan-11   CD         0.020
HX2      plan-11   H11        0.020
HX2      plan-12   CE         0.020
HX2      plan-12   CD         0.020
HX2      plan-12   C2A        0.020
HX2      plan-12   CG         0.020
HX2      plan-13   CBA        0.020
HX2      plan-13   CGA        0.020
HX2      plan-13   O2A        0.020
HX2      plan-13   O1A        0.020
HX2      plan-14   C4A        0.020
HX2      plan-14   C2A        0.020
HX2      plan-14   C3A        0.020
HX2      plan-14   CMA        0.020
HX2      plan-15   C3A        0.020
HX2      plan-15   CMA        0.020
HX2      plan-15   H17        0.020
HX2      plan-15   H18        0.020
HX2      plan-16   CHA        0.020
HX2      plan-16   C4D        0.020
HX2      plan-16   ND         0.020
HX2      plan-16   C3D        0.020
HX2      plan-17   ND         0.020
HX2      plan-17   C1D        0.020
HX2      plan-17   CHD        0.020
HX2      plan-17   C2D        0.020
HX2      plan-18   C4C        0.020
HX2      plan-18   C1D        0.020
HX2      plan-18   CHD        0.020
HX2      plan-18   H19        0.020
HX2      plan-19   C1D        0.020
HX2      plan-19   C2D        0.020
HX2      plan-19   CMD        0.020
HX2      plan-19   C3D        0.020
HX2      plan-20   C4D        0.020
HX2      plan-20   C2D        0.020
HX2      plan-20   C3D        0.020
HX2      plan-20   CAD        0.020
HX2      plan-21   CBD        0.020
HX2      plan-21   CGD        0.020
HX2      plan-21   O1D        0.020
HX2      plan-21   O2D        0.020
HX2      plan-22   C1B        0.020
HX2      plan-22   C2B        0.020
HX2      plan-22   CMB        0.020
HX2      plan-22   C3B        0.020
HX2      plan-23   C4B        0.020
HX2      plan-23   C2B        0.020
HX2      plan-23   C3B        0.020
HX2      plan-23   CAB        0.020
HX2      plan-24   CA         0.020
HX2      plan-24   C          0.020
HX2      plan-24   O          0.020
HX2      plan-24   OXT        0.020

data_mod_HX2mod1

loop_
_chem_mod_atom.mod_id
_chem_mod_atom.function
_chem_mod_atom.atom_id
```

```
_chem_mod_atom.new_atom_id
_chem_mod_atom.new_type_symbol
_chem_mod_atom.new_type_energy
_chem_mod_atom.new_charge
HX2mod1  delete H5     .      .    .        .
HX2mod1  delete S1     .      .    .        .

loop_
_chem_mod_bond.mod_id
_chem_mod_bond.function
_chem_mod_bond.atom_id_1
_chem_mod_bond.atom_id_2
_chem_mod_bond.new_type
_chem_mod_bond.new_aromatic
_chem_mod_bond.new_value_dist
_chem_mod_bond.new_value_dist_esd
HX2mod1  change CBC    CAC       .        .    1.525    0.015
HX2mod1  delete CAC    S1        single   n        .        .
HX2mod1  delete S1     H5        single   n        .        .

loop_
_chem_mod_angle.mod_id
_chem_mod_angle.function
_chem_mod_angle.atom_id_1
_chem_mod_angle.atom_id_2
_chem_mod_angle.atom_id_3
_chem_mod_angle.new_value_angle
_chem_mod_angle.new_value_angle_esd
HX2mod1  delete CBC    CAC    S1            .        .
HX2mod1  delete S1     CAC    C3C           .        .
HX2mod1  delete S1     CAC    H4            .        .
HX2mod1  delete CAC    S1     H5            .        .
HX2mod1  change CAC    C3C    C2C     127.355    1.500

loop_
_chem_mod_chir.mod_id
_chem_mod_chir.function
_chem_mod_chir.id
_chem_mod_chir.atom_id_centre
_chem_mod_chir.atom_id_1
_chem_mod_chir.atom_id_2
_chem_mod_chir.atom_id_3
_chem_mod_chir.new_volume_sign
HX2mod1  delete chir_1   CAC    CBC    S1     C3C       positiv

data_mod_CYSmod1

loop_
_chem_mod_atom.mod_id
_chem_mod_atom.function
_chem_mod_atom.atom_id
_chem_mod_atom.new_atom_id
_chem_mod_atom.new_type_symbol
_chem_mod_atom.new_type_energy
_chem_mod_atom.new_charge
CYSmod1  change SG     .      .    S        .
CYSmod1  add    .      HSG1   H    HSH1     0

loop_
_chem_mod_bond.mod_id
```

```
_chem_mod_bond.function
_chem_mod_bond.atom_id_1
_chem_mod_bond.atom_id_2
_chem_mod_bond.new_type
_chem_mod_bond.new_aromatic
_chem_mod_bond.new_value_dist
_chem_mod_bond.new_value_dist_esd
CYSmod1  change CB     SG        .        .    1.778    0.010
CYSmod1  add    SG     HSG1      single   n    1.225    0.020

loop_
_chem_mod_angle.mod_id
_chem_mod_angle.function
_chem_mod_angle.atom_id_1
_chem_mod_angle.atom_id_2
_chem_mod_angle.atom_id_3
_chem_mod_angle.new_value_angle
_chem_mod_angle.new_value_angle_esd
CYSmod1  change N      CA     CB      112.343    1.500
CYSmod1  change CB     CA     HA      106.687    1.500
CYSmod1  change CA     CB     SG      110.778    3.000
CYSmod1  change CA     CB     HB3     108.012    1.500
CYSmod1  change CA     CB     HB2     108.012    1.500
CYSmod1  change CB     SG     HG      109.471    3.000
CYSmod1  add    CB     SG     HSG1    109.471    3.000
CYSmod1  add    HG     SG     HSG1    109.471    3.000

data_mod_CYSmod2

loop_
_chem_mod_atom.mod_id
_chem_mod_atom.function
_chem_mod_atom.atom_id
_chem_mod_atom.new_atom_id
_chem_mod_atom.new_type_symbol
_chem_mod_atom.new_type_energy
_chem_mod_atom.new_charge
CYSmod2  change SG     .      .    S        .
CYSmod2  add    .      HSG1   H    HSH1     0

loop_
_chem_mod_bond.mod_id
_chem_mod_bond.function
_chem_mod_bond.atom_id_1
_chem_mod_bond.atom_id_2
_chem_mod_bond.new_type
_chem_mod_bond.new_aromatic
_chem_mod_bond.new_value_dist
_chem_mod_bond.new_value_dist_esd
CYSmod2  change CB     SG        .        .    1.778    0.010
CYSmod2  add    SG     HSG1      single   n    1.225    0.020

loop_
_chem_mod_angle.mod_id
_chem_mod_angle.function
_chem_mod_angle.atom_id_1
_chem_mod_angle.atom_id_2
_chem_mod_angle.atom_id_3
_chem_mod_angle.new_value_angle
_chem_mod_angle.new_value_angle_esd
```

```
CYSmod2  change N      CA     CB      112.343    1.500
CYSmod2  change CB     CA     HA      106.687    1.500
CYSmod2  change CA     CB     SG      110.778    3.000
CYSmod2  change CA     CB     HB3     108.012    1.500
CYSmod2  change CA     CB     HB2     108.012    1.500
CYSmod2  change CB     SG     HG      109.471    3.000
CYSmod2  add    CB     SG     HSG1    109.471    3.000
CYSmod2  add    HG     SG     HSG1    109.471    3.000

data_mod_HX2mod2

loop_
_chem_mod_atom.mod_id
_chem_mod_atom.function
_chem_mod_atom.atom_id
_chem_mod_atom.new_atom_id
_chem_mod_atom.new_type_symbol
_chem_mod_atom.new_type_energy
_chem_mod_atom.new_charge
HX2mod2  delete H34    .      .    .        .
HX2mod2  delete S2     .      .    .        .

loop_
_chem_mod_bond.mod_id
_chem_mod_bond.function
_chem_mod_bond.atom_id_1
_chem_mod_bond.atom_id_2
_chem_mod_bond.new_type
_chem_mod_bond.new_aromatic
_chem_mod_bond.new_value_dist
_chem_mod_bond.new_value_dist_esd
HX2mod2  change CAB    CBB       .        .    1.525    0.015
HX2mod2  delete CAB    S2        single   n        .        .
HX2mod2  delete S2     H34       single   n        .        .

loop_
_chem_mod_angle.mod_id
_chem_mod_angle.function
_chem_mod_angle.atom_id_1
_chem_mod_angle.atom_id_2
_chem_mod_angle.atom_id_3
_chem_mod_angle.new_value_angle
_chem_mod_angle.new_value_angle_esd
HX2mod2  change C4B    C3B    CAB     124.022    3.000
HX2mod2  change C2B    C3B    CAB     129.176    1.500
HX2mod2  delete C3B    CAB    S2            .        .
HX2mod2  delete CBB    CAB    S2            .        .
HX2mod2  delete S2     CAB    H30           .        .
HX2mod2  delete CAB    S2     H34           .        .

loop_
_chem_mod_chir.mod_id
_chem_mod_chir.function
_chem_mod_chir.id
_chem_mod_chir.atom_id_centre
_chem_mod_chir.atom_id_1
_chem_mod_chir.atom_id_2
_chem_mod_chir.atom_id_3
_chem_mod_chir.new_volume_sign
HX2mod2  delete chir_3   CAB    C3B    CBB    S2        positiv
```

```
data_link_HX2-CYS

loop_
_chem_link_bond.link_id
_chem_link_bond.atom_1_comp_id
_chem_link_bond.atom_id_1
_chem_link_bond.atom_2_comp_id
_chem_link_bond.atom_id_2
_chem_link_bond.type
_chem_link_bond.aromatic
_chem_link_bond.value_dist
_chem_link_bond.value_dist_esd
HX2-CYS  1 CAC    2 SG        single   n    1.811    0.020

loop_
_chem_link_angle.link_id
_chem_link_angle.atom_1_comp_id
_chem_link_angle.atom_id_1
_chem_link_angle.atom_2_comp_id
_chem_link_angle.atom_id_2
_chem_link_angle.atom_3_comp_id
_chem_link_angle.atom_id_3
_chem_link_angle.value_angle
_chem_link_angle.value_angle_esd
HX2-CYS  1 CBC    1 CAC    2 SG      107.734    3.000
HX2-CYS  1 C3C    1 CAC    2 SG      112.507    3.000
HX2-CYS  1 H4     1 CAC    2 SG      107.260    1.500
HX2-CYS  1 CAC    2 SG     2 CB      104.305    3.000
HX2-CYS  1 CAC    2 SG     2 HG      109.471    3.000
HX2-CYS  1 CAC    2 SG     2 HSG1    109.471    3.000

loop_
_chem_link_chir.link_id
_chem_link_chir.id
_chem_link_chir.atom_centre_comp_id
_chem_link_chir.atom_id_centre
_chem_link_chir.atom_1_comp_id
_chem_link_chir.atom_id_1
_chem_link_chir.atom_2_comp_id
_chem_link_chir.atom_id_2
_chem_link_chir.atom_3_comp_id
_chem_link_chir.atom_id_3
_chem_link_chir.volume_sign
HX2-CYS  chir_5   1 CAC    1 CBC    2 SG     1 C3C       positiv

data_link_CYS-HX2

loop_
_chem_link_bond.link_id
_chem_link_bond.atom_1_comp_id
_chem_link_bond.atom_id_1
_chem_link_bond.atom_2_comp_id
_chem_link_bond.atom_id_2
_chem_link_bond.type
_chem_link_bond.aromatic
_chem_link_bond.value_dist
_chem_link_bond.value_dist_esd
CYS-HX2  1 SG     2 CAB       single   n    1.811    0.020
```

```
loop_
_chem_link_angle.link_id
_chem_link_angle.atom_1_comp_id
_chem_link_angle.atom_id_1
_chem_link_angle.atom_2_comp_id
_chem_link_angle.atom_id_2
_chem_link_angle.atom_3_comp_id
_chem_link_angle.atom_id_3
_chem_link_angle.value_angle
_chem_link_angle.value_angle_esd
CYS-HX2  1 SG     2 CAB    2 C3B     111.378    3.000
CYS-HX2  1 SG     2 CAB    2 CBB     107.734    3.000
CYS-HX2  1 SG     2 CAB    2 H30     107.260    1.500
CYS-HX2  1 CB     1 SG     2 CAB     104.305    3.000
CYS-HX2  1 HG     1 SG     2 CAB     109.471    3.000
CYS-HX2  1 HSG1   1 SG     2 CAB     109.471    3.000

loop_
_chem_link_chir.link_id
_chem_link_chir.id
_chem_link_chir.atom_centre_comp_id
_chem_link_chir.atom_id_centre
_chem_link_chir.atom_1_comp_id
_chem_link_chir.atom_id_1
_chem_link_chir.atom_2_comp_id
_chem_link_chir.atom_id_2
_chem_link_chir.atom_3_comp_id
_chem_link_chir.atom_id_3
_chem_link_chir.volume_sign
CYS-HX2  chir_5  2 CAB   1 SG    2 C3B   2 CBB      positiv
```

RADDOSE input

Used for calculating the dose of the ferrous structure from the first dataset. Dose multiplied by 50/20 for the second dataset.

```
##############################################################################
#                               Crystal Block                                #
##############################################################################

Crystal

Type Cylinder
       # Crystal shape can be Cuboid or Spherical

Dimensions 25 100
       # Diameter and Height

PixelsPerMicron 1
       # This defines the coarseness of the simulation
       # (i.e. how many voxels the crystal is divided into.)
       # Preferably set as high as possible, however for a higher
       # value the simulation will take longer to complete.
       # Recommended to try increasing between 0.5 and 5 and ensure
       # the reported dose value converges as PixelsPerMicron increases.
       # As a rule of thumb, this needs to be at least 10x the beam
       # FWHM for a Gaussian beam.
       # e.g. 20µm FWHM beam -> 2µm voxels -> 0.5 voxels/µm

# NOTE: Use AngleP/AngleL if your crystal is not face-on to the beam.
# See RD3D user guide for more details
ANGLEP 23
```

```
# Also need to specify the crystal composition below (Example case for insulin given):
AbsCoefCalc  RD3D
       # Absorption Coefficients calculated
       # using RADDOSE-3D (Zeldin et al. 2013).


UnitCell   46.380   80.441   84.780
       # unit cell size: a, b, c with alpha, beta and gamma angles default to 90°


NumMonomers  8
       # number of monomers in unit cell


NumResidues  144
       # number of residues per monomer


ProteinHeavyAtoms Fe 1 S 6
       # heavy atoms added to protein part of the
       # monomer, i.e. S, coordinated metals, Se in Se-Met


SolventHeavyConc S 1910 Na 2127
       # concentration of elements in the solvent
       # in mmol/l. Oxygen and lighter elements
       # should not be specified


SolventFraction 0.5247
       # fraction of the unit cell occupied by solvent

##############################################################################
#                                  Beam Block                                #
##############################################################################

Beam


Type Gaussian
       # beam profile can be Gaussian or TopHat
Flux 0.3e12
       # in photons per second (2e12 = 2 * 10^12)
FWHM 50 50
       # in µm, horizontal by vertical for a Gaussian beam
Energy 20
       # photon energy in keV


Collimation Rectangular 150 150
       # Horizontal/Vertical collimation of the beam
       # For 'uncollimated' Gaussians, 3xFWHM recommended



##############################################################################
#                                  Wedge Block                               #
##############################################################################


Wedge -90 90
       # Start and End rotational angle of the crystal with Start < End


ExposureTime 18
       # Total time for entire angular range (seconds)
```

References

1 R. B. Best, X. Zhu, J. Shim, P. E. M. Lopes, J. Mittal, M. Feig and A. D. Jr. MacKerell, *J. Chem. Theory Comput.*, 2012, **8**, 3257–3273.

2 J. Huang, S. Rauscher, G. Nawrocki, T. Ran, M. Feig, B. L. de Groot, H. Grubmüller and A. D. MacKerell, *Nat. Methods*, 2017, **14**, 71–73.

3 E. Aprà, E. J. Bylaska, W. A. de Jong, N. Govind, K. Kowalski, T. P. Straatsma, M. Valiev, H. J. J. van Dam, Y. Alexeev, J. Anchell, V. Anisimov, F. W. Aquino, R. Atta-Fynn, J. Autschbach, N. P. Bauman, J. C. Becca, D. E. Bernholdt, K. Bhaskaran-Nair, S. Bogatko, P. Borowski, J. Boschen, J. Brabec, A. Bruner, E. Cauët, Y. Chen, G. N. Chuev, C. J. Cramer, J. Daily, M. J. O. Deegan, T. H. Dunning, M. Dupuis, K. G. Dyall, G. I. Fann, S. A. Fischer, A. Fonari, H. Früchtl, L. Gagliardi, J. Garza, N. Gawande, S. Ghosh, K. Glaesemann, A. W. Götz, J. Hammond, V. Helms, E. D. Hermes, K. Hirao, S. Hirata, M. Jacquelin, L. Jensen, B. G. Johnson, H. Jónsson, R. A. Kendall, M. Klemm, R. Kobayashi, V. Konkov, S. Krishnamoorthy, M. Krishnan, Z. Lin, R. D. Lins, R. J. Littlefield, A. J. Logsdail, K. Lopata, W. Ma, A. V. Marenich, J. Martin del Campo, D. Mejia-Rodriguez, J. E. Moore, J. M. Mullin, T. Nakajima, D. R. Nascimento, J. A. Nichols, P. J. Nichols, J. Nieplocha, A. Otero-de-la-Roza, B. Palmer, A. Panyala, T. Pirojsirikul, B. Peng, R. Peverati, J. Pittner, L. Pollack, R. M. Richard, P. Sadayappan, G. C. Schatz, W. A. Shelton, D. W. Silverstein, D. M. A. Smith, T. A. Soares, D. Song, M. Swart, H. L. Taylor, G. S. Thomas, V. Tipparaju, D. G. Truhlar, K. Tsemekhman, T. Van Voorhis, Á. Vázquez-Mayagoitia, P. Verma, O. Villa, A. Vishnu, K. D. Vogiatzis, D. Wang, J. H. Weare, M. J. Williamson, T. L. Windus, K. Woliński, A. T. Wong, Q. Wu, C. Yang, Q. Yu, M. Zacharias, Z. Zhang, Y. Zhao and R. J. Harrison, *J. Chem. Phys.*, 2020, **152**, 184102.

4 A. D. Becke, *J. Chem. Phys.*, 1993, **98**, 5648–5652.

5 C. Lee, W. Yang and R. G. Parr, *Phys. Rev. B*, 1988, **37**, 785–789.

6 S. Grimme, J. Antony, S. Ehrlich and H. Krieg, *J. Chem. Phys.*, 2010, **132**, 154104.

7 R. Lonsdale, J. N. Harvey and A. J. Mulholland, *J. Phys. Chem. Lett.*, 2010, **1**, 3232–3237.

8 G. A. Petersson, A. Bennett, T. G. Tensfeldt, M. A. Al-Laham, W. A. Shirley and J. Mantzaris, *J. Chem. Phys.*, 1988, **89**, 2193–2218.

9 G. A. Petersson and M. A. Al-Laham, *J. Chem. Phys.*, 1991, **94**, 6081–6090.
